# The OGT–c-Myc–PDK2 axis rewires the TCA cycle and promotes colorectal tumor growth

**DOI:** 10.1038/s41418-024-01315-4

**Published:** 2024-05-22

**Authors:** Huijuan Wang, Jie Sun, Haofan Sun, Yifei Wang, Bingyi Lin, Liming Wu, Weijie Qin, Qiang Zhu, Wen Yi

**Affiliations:** 1 Department of Biochemistry, College of Life Sciences, Zhejiang University, Hangzhou, 310058 China; 2 National Center for Protein Sciences Beijing, State Key Laboratory of Proteomics, Beijing Proteome Research Center, Beijing Institute of Lifeomics, Beijing, 100026 China; 3 Department of Hepatobiliary and Pancreatic Surgery, The First Affiliated Hospital, Zhejiang Provincial Key Laboratory of Pancreatic Disease, School of Medicine, Zhejiang University, Hangzhou, 310003 China; 4 Cancer Center, Zhejiang University, Hangzhou, 310003 China

**Keywords:** Glycobiology, Metabolic pathways

## Abstract

Deregulated glucose metabolism termed the “Warburg effect” is a fundamental feature of cancers, including the colorectal cancer. This is typically characterized with an increased rate of glycolysis, and a concomitant reduced rate of the tricarboxylic acid (TCA) cycle metabolism as compared to the normal cells. How the TCA cycle is manipulated in cancer cells remains unknown. Here, we show that *O*-linked *N*-acetylglucosamine (O-GlcNAc) regulates the TCA cycle in colorectal cancer cells. Depletion of OGT, the sole transferase of O-GlcNAc, significantly increases the TCA cycle metabolism in colorectal cancer cells. Mechanistically, OGT-catalyzed O-GlcNAc modification of c-Myc at serine 415 (S415) increases c-Myc stability, which transcriptionally upregulates the expression of pyruvate dehydrogenase kinase 2 (PDK2). PDK2 phosphorylates pyruvate dehydrogenase (PDH) to inhibit the activity of mitochondrial pyruvate dehydrogenase complex, which reduces mitochondrial pyruvate metabolism, suppresses reactive oxygen species production, and promotes xenograft tumor growth. Furthermore, c-Myc S415 glycosylation levels positively correlate with PDK2 expression levels in clinical colorectal tumor tissues. This study highlights the OGT–c-Myc–PDK2 axis as a key mechanism linking oncoprotein activation with deregulated glucose metabolism in colorectal cancer.

## Introduction

Many cancer cells exhibit enhanced rates of glycolysis with concomitant reduction of tricarboxylic acid (TCA) cycle metabolism and subsequent oxidative phosphorylation (OXPHOS) [1–3]. This altered glucose metabolism, termed aerobic glycolysis, is considered as a critical means to provide biosynthetic intermediates to fuel cancer cell growth and proliferation [4, 5]. How cancer cells regulate glycolysis and the TCA cycle metabolism is an area of active scientific investigation. The pyruvate dehydrogenase complex (PDC) converts pyruvate to acetyl-CoA at the crossroads between glycolysis and the TCA cycle and plays a critical role in this metabolic transition [6, 7]. Pyruvate dehydrogenase kinases (PDKs) are the well-known regulators of PDC by phosphorylating pyruvate dehydrogenase (PDH) to inhibit the activity of PDC, thus blocking the TCA cycle and oxidative phosphorylation [6–9]. There are four PDK isoforms in mammals with differential tissue expressions [10–12]. Among them, PDK2 is highly expressed in a variety of tumors, and correlated with poor prognosis in patients with prostate cancer, acute myeloid leukemia, lung adenocarcinoma and head and neck cancer [13–17]. Although accumulating evidence has shown the pivotal roles of PDK2 in tumor cell proliferation, migration and drug resistance, how PDK2 is regulated in cancer cells is largely unknown.

*O*-linked *N*-acetylglucosamine (O-GlcNAc) is a dynamic monosaccharide modification of numerous nucleocytoplasmic proteins in mammals [18, 19]. The sugar donor of this modification, uridine 5-diphospho-N-acetylglucosamine (UDP-GlcNAc), is derived from the hexosamine biosynthetic pathway (HBP), a branch of glycolysis. O-GlcNAc transferase (OGT) is the principal enzyme catalyzing the conjugation of the GlcNAc residue onto proteins, while O-GlcNAcase (OGA) is responsible for hydrolysis of GlcNAc from proteins [20–22]. Highly responsive to fluctuating nutrient status in cells, O-GlcNAcylation is shown to regulate many important biological events including gene transcription, protein quality control, signal transduction, and immune responses [21, 23, 24]. Recently, accumulating studies have showed that O-GlcNAcylation is involved in cellular metabolic reprogramming through directly or indirectly modulating metabolic enzymes [25, 26]. For example, O-GlcNAcylation of glycolytic enzymes redirects glucose flux for anabolic biosynthesis and redox homeostasis [23]. However, how O-GlcNAcylation regulates the TCA cycle remains elusive.

MYC (also known as c-Myc) is a transcription factor overexpressed in over 70% of human cancers, which is associated with poor patient survival [27–29]. The c-Myc governs the expression of numerous genes involved in a variety of biological processes [30, 31]. The activation of c-Myc is closely linked to multiple hallmarks of cancer, such as uncontrolled proliferation, distant organ metastasis, immune evasion, genomic instability, and metabolic reprogramming [27, 32–34]. The upregulation of c-Myc in tumors is shown to be mediated by genomic alterations, such as amplification of gene, chromosomal translocations and aberration [27, 35]. Post-translational modifications also contribute to c-Myc expression levels by modulating the protein stability [36, 37]. For example, poly-ubiquitination of c-Myc by the E3 ligases FBXW7, HectH9, TRIM32 and MAGI3 promotes c-Myc degradation, while USP28 antagonizes the above process [38, 39]. Phosphorylation at S62 induced by ERK signaling increases, while phosphorylation at T58 reduces, c-Myc stability [39]. It was also reported that O-GlcNAcylation at Thr58 enhances c-Myc stability by competitively inhibiting phosphorylation at the same site [40]. Thus, c-Myc protein is subjected to complex regulation in cells, understanding of which is far from complete.

In this study, we report a novel mechanism by which O-GlcNAcylation regulates the TCA cycle in colorectal cancer. O-GlcNAcylation repressed the TCA cycle by enhancing the transcriptional expression of PDK2, which inhibited PDH-mediated mitochondrial pyruvate metabolism, thereby reducing reactive oxygen species (ROS) production and promoting cell proliferation and tumor growth. Mechanistically, O-GlcNAcylation dynamically modified c-Myc at Ser415 to block its association with the E3 ligase MAGI3, impeding the proteasome degradation of c-Myc in cells. The accumulated c-Myc could bind to the promoter region of PDK2 to activate PDK2 transcription. Blocking Ser415 O-GlcNAcylation on c-Myc suppressed PDK2 expression, enhanced ROS levels, and decreased colorectal cancer cell proliferation and tumor growth in nude mice. Our findings reveal that the OGT–c-Myc–PDK2 axis plays a key role in regulating glucose metabolism in colorectal cancer, and suggest new therapeutic strategies.

## Results

### OGT expression is critical for colorectal tumor growth and metabolism

**Fig. 1:**
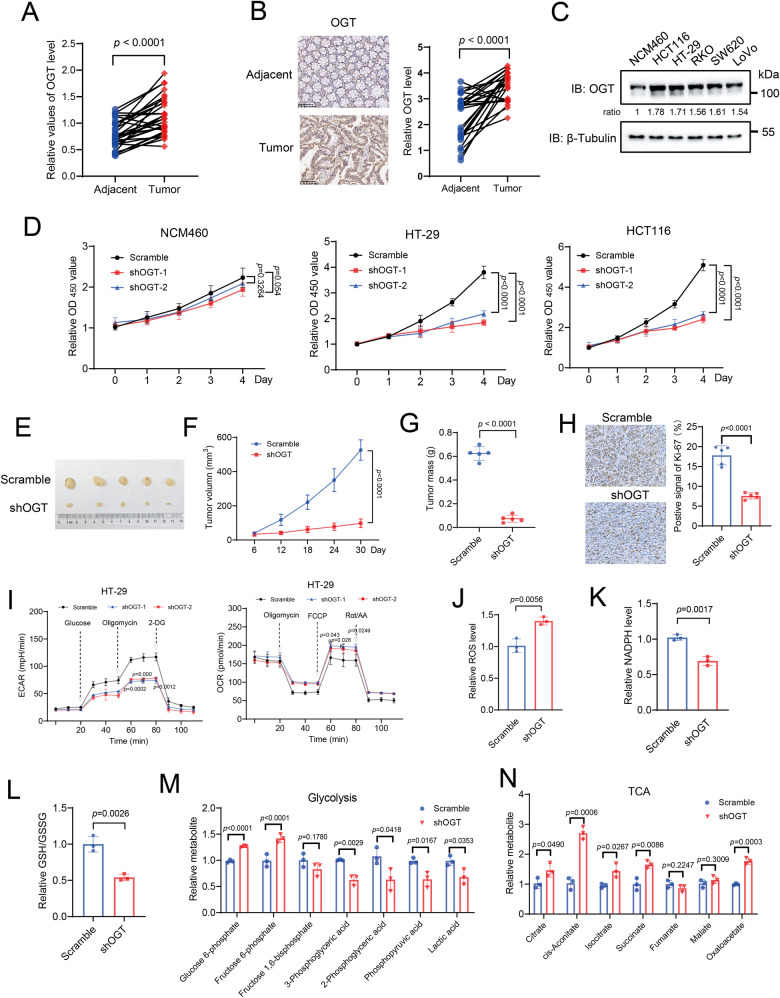
OGT expression is critical for colorectal tumor growth and metabolism. **A** Quantification analysis of OGT expression levels from human colon tumor (T) and the matching non-tumor (N) tissue samples (*n*  =  30). **B** Immunohistochemistry staining of human CRC samples with OGT antibody (Scale bar, 100 μm). The relative OGT expression was quantified based on intensity and area of the staining. **C** Immunoblotting analysis of OGT expression using colon cancer cell lines (HCT116, HT-29, RKO, SW620 and LoVo) and a normal colon cell line (NCM460). **D** Cell proliferation of NCM460, HT-29, HCT116 cells upon OGT knockdown with small hairpin RNAs (shRNA1 and 2). *n* = 5; Data are presented as means ± SD. *P* values were determined by unpaired two-tailed Student’s *t* tests. **E** HT-29 cells expressing scramble or shOGT were subcutaneously injected into 6-week-old male nude mice (*n* = 5 per group). Images of dissected tumors from mice. **F** Growth curves of tumors measured at indicated time points. *n* = 5; Data are presented as means ± SD. *P* values were determined by unpaired two-tailed Student’s *t* tests. **G** Tumor mass measured at the experimental endpoint. *n* = 5; Data are presented as means ± SD. *P* values were determined by unpaired two-tailed Student’s *t* tests. **H** Ki67 staining of tumors generated from HT-29 cells infected with scramble or shOGT (Scale bar, 100 μm). Quantification was shown. *n* = 5; Data are presented as means ± SD. *P* values were determined by unpaired two-tailed Student’s *t* tests. **I** Analysis of extracellular acidification rate (ECAR) and oxygen consumption rate (OCR) in HT-29 cells expressing scramble or shOGT. *n* = 3; Data are presented as means ± SD. *P* values were determined by unpaired two-tailed Student’s *t* tests. Analysis of ROS (**J**), NADPH (**K**), GSH/GSSG (**L**) in HT-29 cells expressing scramble or shOGT. *n* = 3; Data are presented as means ± SD. *P* values were determined by unpaired two-tailed Student’s *t* tests. Relative abundance of metabolites derived from glycolysis (**M**) and the TCA cycle (**N**) in HT-29 cells expressing scramble or shOGT. *n* = 3; Data are presented as means ± SD. *P* values were determined by unpaired two-tailed Student’s *t* tests.

OGT expression is elevated in various types of cancers [41–44]. To corroborate with these findings, we analyzed OGT mRNA expression in tumor tissues and the matching normal tissues based on The Cancer Genome Atlas (TCGA) gene expression datasets. OGT expression was significantly upregulated in colorectal cancer (CRC), stomach adenocarcinoma (STAD), lung adenocarcinoma (LUAD), liver hepatocellular carcinoma (LIHC) and prostate adenocarcinoma (PRED) tissues compared to the adjacent normal tissues (Fig. S1A). Kaplan-Meier analysis revealed that elevated OGT expression were associated with poor survival of patients with colorectal cancer and liver hepatocellular carcinoma (Fig. S1B). To further investigate the function of OGT in colorectal cancer, we performed western blotting and immunohistochemistry (IHC) analyses of OGT expression in 30 pairs of human colorectal cancer tissues and matching peritumoral tissues. The result showed that OGT expression levels were significantly higher in cancer tissues compared to adjacent normal tissues (Figs. 1A, B and S1C). Consistently, both mRNA and protein levels of OGT were significantly increased in a panel of colorectal cancer cell lines (HCT116, HT-29, RKO, SW620, LoVo) compared to a normal colon epithethial cell NCM460 (Fig. 1C and S1D). We then depleted OGT expression in HT-29, HCT116, and NCM460 cells using small hairpin RNAs (shRNAs), and analyzed cell proliferation. OGT depletion expectedly reduced cellular O-GlcNAcylation levels in all three cell lines, and caused a significant decrease of cell proliferation in both HT-29 and HCT116 cells, while only slightly affecting NCM460 cells (Fig. 1D and S2A). To further probe the effect of OGT deficiency on tumor growth, we subcutaneously injected control or OGT-depleted HT-29 cells into nude mice. In line with above findings, OGT-depleted HT-29 cells developed tumors more slowly, with smaller sizes and masses, and lower positive signals of Ki-67 compared to control cells (Fig. 1E–H). Together, these results demonstrated that OGT expression is crucial for colorectal cancer cell proliferation and tumor growth.

To evaluate whether OGT impacts glucose metabolism, we analyzed the extracellular acidification rate (ECAR) and oxygen consumption rate (OCR) in OGT-depleted or control NCM460 and HT-29 cells. The results showed that compared to the control, OGT depletion caused a significant reduction of ECAR, and a significant increase of OCR, indicating a reduced glycolytic activity, and an enhanced mitochondrial oxidation activity in HT-29 cells, but not in NCM460 cells (Fig. 1I and S2B). Similar observations were obtained when cells were incubated with OSMI4, a specific OGT inhibitor, suggesting that OGT regulated glucose metabolism dependent on its enzymatic activity (Fig. S2C, D). In addition, glucose uptake and the level of adenosine triphosphate (ATP) were significantly reduced upon OGT depletion in HT-29 cells compared to the control (Fig. S2E, F). In contrast, OGT depletion promoted reactive oxygen species (ROS) levels, and decreased the relative levels of nicotinamide adenine dinucleotide phosphate (NADPH) and reduced glutathione (GSH) in HT-29 cells (Fig. 1J–L). To further corroborate this, we quantified metabolite levels by liquid chromatography coupled with mass spectrometry (LC-MS) analysis. OGT depletion increased the levels of glucose-6-phosphate and fructose-6-phosphate, metabolites at the early phase of the glycolysis pathway, while decreasing the levels of phosphopyruvic acid and lactic acid, metabolites at the late phase of glycolysis pathway (Fig. 1M). The increased levels of glucose-6-phosphate likely shunt into the pentose phosphate pathway. Consistently, levels of key metabolites in the pentose phosphate pathway were increased significantly upon OGT depletion (Fig. S2G). In agreement with the increase of OCR, levels of metabolites in the TCA cycle were increased (Fig. 1N). Together, OGT depletion caused a downregulation of glycolysis, but upregulation of the TCA cycle in colorectal cancer cells.

### OGT depletion upregulates the TCA cycle by transcriptional repression of PDK2

**Fig. 2:**
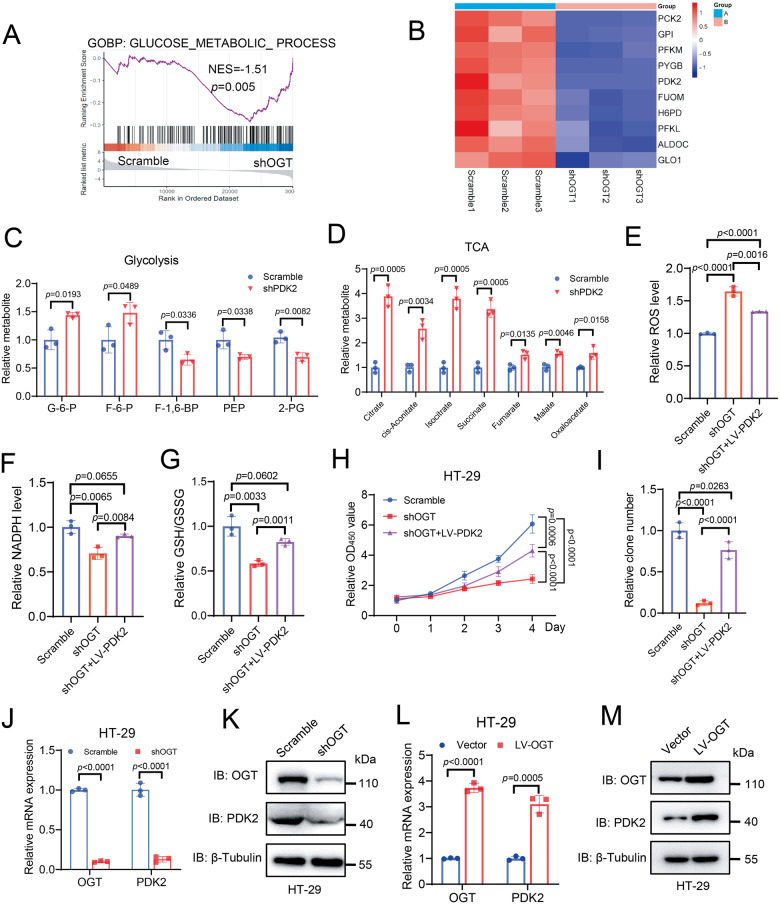
OGT depletion upregulates the TCA cycle by transcriptional repression of PDK2. **A** GSEA analysis of OGT-regulated gene signature versus glucose metabolic process. **B** Heatmap showing the top 10 downregulated genes of glucose metabolism from the RNA-seq analysis. Relative abundance of metabolites derived from glycolysis (**C**) and TCA cycle (**D**) HT-29 cells expressing scramble or shPDK2. *n* = 3; Data are presented as means ± SD. *P* values were determined by unpaired two-tailed Student’s *t* tests. Comparison of ROS (**E**), NADPH (**F**), GSH/GSSG (**G**) in HT-29 cells expressing scramble, shOGT or shOGT reconstituted with PDK2 expression. *n* = 3; Data are presented as means ± SD. *P* values were determined by unpaired two-tailed Student’s *t* tests. Cell proliferation (**H**) and clone formation (**I**) of HT-29 cells expressing scramble, shOGT or shOGT with reconstituted PDK2 expression. *n* = 5; Data are presented as means ± SD. *P* values were determined by unpaired two-tailed Student’s *t* tests. Quantitative PCR analysis (**J**) and immunoblotting analysis (**K**) of PDK2 expression in HT-29 cells expressing scramble or shOGT. *n* = 3; Data are presented as means ± SD. *P* values were determined by unpaired two-tailed Student’s *t* tests. Quantitative PCR analysis (**L**) and immunoblotting analysis (**M**) of PDK2 expression in HT-29 cells expressing control vector or LV-OGT. *n* = 3; Data are presented as means ± SD. *P* values were determined by unpaired two-tailed Student’s *t* tests.

To further investigate the mechanism by which OGT depletion upregulates the TCA cycle, we performed transcriptomic analysis by RNA sequencing (RNA-seq) of HT-29 cells with or without OGT depletion. There were 2987 and 2694 differentially expressed genes (DEGs) with fold change >2 and *p* value < 0.05 significantly upregulated or downregulated in OGT-depleted HT-29 cells, respectively, compared to control cells (Fig. S3A). Gene set enrichment analysis (GSEA) showed marked enrichment of glucose metabolic pathways, mainly composed of glycolysis, the pentose phosphate pathway and the TCA cycle (Fig. 2A). Among the top 10 downregulated genes in glucose metabolism, *PDK2* encoding pyruvate dehydrogenase kinase 2 is directly involved in the TCA cycle (Fig. 2B). GPI and PFK1 encode enzymes responsible for metabolizing glucose-6-phosphate and fructose-6-phosphate, respectively. The reduced mRNA expressions of GPI and PFK1 likely lead to accumulation of glucose-6-phosphate and fructose-6-phosphate as shown in Fig. 1M. Selected glucose metabolic genes were verified with real-time PCR (Fig. S3B). PDK2 is a mitochondrial enzyme that catalyzes phosphorylation of pyruvate dehydrogenase (PDH), resulting in PDH inactivation and the subsequent suppression of pyruvate conversion to acetyl-CoA [6, 9]. We confirmed that depletion of PDK2 in NCM460, HT-29, and HCT116 cells resulted in suppression of PDH Ser293 phosphorylation, a known marker of PDH inactivation (Fig. S3C) [7, 45]. The metabolic analysis consistently showed that PDK2 depletion significantly increased OCR, and reduced ECAR (Fig. S3D). The levels of key metabolites at the late phase of glycolysis pathway were reduced, while those in the TCA cycle were upregulated upon PDK2 depletion (Fig. 2C, D). In line with the this, ROS levels were significantly enhanced, while the relative levels of GSH and NADPH were decreased in cells with PDK2 depletion, which phenocopies OGT-depleted cells (Fig. S3E, G). Reconstituted expression of PDK2 in OGT-depleted HT-29 and HCT116 cells partially reduced ROS levels, increased the relative levels of GSH and NADPH, and rescued cell proliferation and colony formation (Fig. 2E–I and S3H–J). Consistently, depletion of PDK2 repressed cell proliferation in HT-29 and HCT116 cells, but not in NCM460 cells (Fig. S3K). To evaluate whether OGT regulates PDK2 expression, we depleted or ectopically expressed OGT in both HT-29 and HCT116 cells, and analyzed PDK2 expression. OGT depletion reduced, while OGT expression increased, both mRNA levels and protein levels of PDK2 (Fig. 2J–M and S3L–O). Together, these data indicated that OGT impacts the TCA cycle by regulating the transcriptional level of PDK2.

### c-Myc directly regulates PDK2 expression

**Fig. 3:**
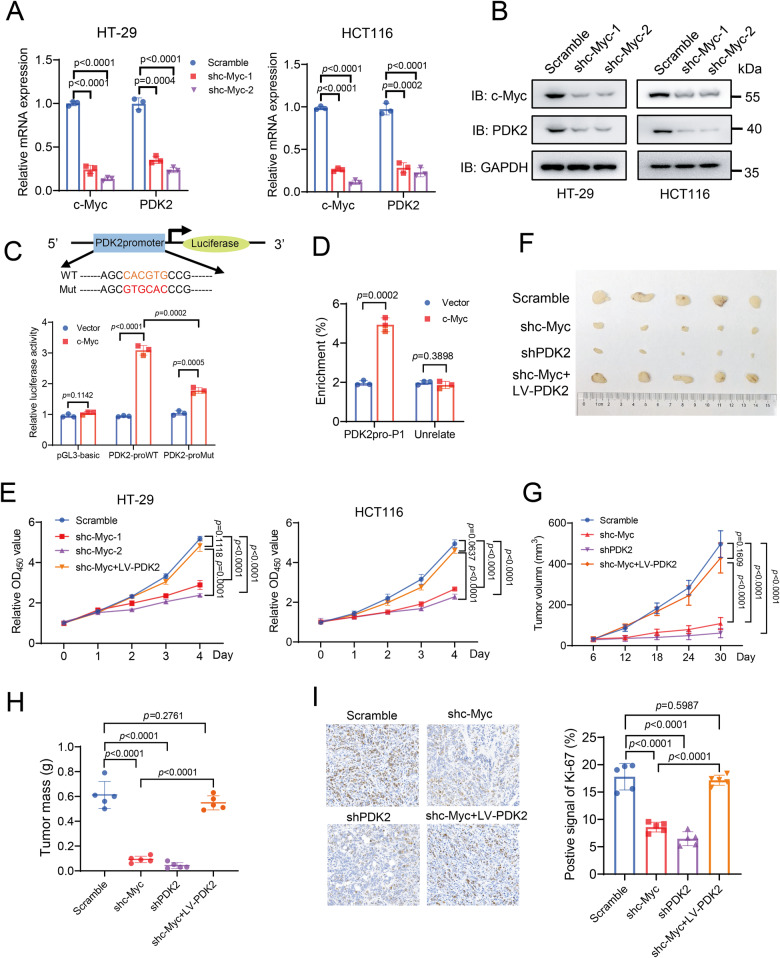
c-MYC directly regulates PDK2 expression. Quantitative PCR analysis (**A**) and immunoblotting analysis (**B**) of PDK2 expression in HT-29 and HCT116 cells infected with scramble or shc-Myc. *n* = 3; Data are presented as means ± SD. *P* values were determined by unpaired two-tailed Student’s *t* tests. **C** Dual-luciferase reporter assay showing the effects of c-Myc overexpression on relative PDK2-promoter activity in the 293 T cells. *n* = 3; Data are presented as means ± SD. *P* values were determined by unpaired two-tailed Student’s *t* tests. **D** ChIP-qPCR analysis of c-Myc binding to the predicted binding regions of PDK2 promoter in HT-29 cells. Ectopic c-Myc was pulled down by the anti-c-Myc antibody. *n* = 3; Data are presented as means ± SD. *P* values were determined by unpaired two-tailed Student’s *t* tests. **E** Cell proliferation of HT-29 and HCT116 infected with Scramble, shc-Myc or shc-Myc reconstituted with PDK2 expression. *n* = 5; Data are presented as means ± SD. *P* values were determined by unpaired two-tailed Student’s *t* tests. **F** Xenograft analysis of HT-29 cells expressing Scramble, shc-Myc, shPDK2 or shc-Myc reconstituted with PDK2 expression. Images of dissected tumors from mice. Analysis of tumor growth (**G**), tumor weights (**H**) and Ki67 staining (**I**) of tumors generated from HT-29 cells infected with scramble, shc-Myc, shPDK2 or shc-Myc reconstituted with PDK2 expression. Scale bars: 100 μm. Quantification was shown. *n* = 5; Data are presented as means ± SD. *P* values were determined by unpaired two-tailed Student’s *t* tests.

To investigate how OGT regulates PDK2 transcription, we firstly sought to identify the transcription factor that directly regulates PDK2 expression. Using the online bioinformatics tool JASPAR, we obtain potential transcription factors that likely bind to promoter regions of PDK2. The diagram reveals that there is one binding site of c-Myc at the promoter region of PDK2 ( − 201 to −212 bp), implicating c-Myc as a potential transcription factor of PDK2 (Fig. S4A). To verify that c-Myc transcriptionally regulates PDK2 expression, we depleted c-Myc in HT-29 and HCT116 cells, and observed that PDK2 expression was decreased in both mRNA and protein levels (Fig. 3A, B). In contrast, ectopic expression of c-Myc in cells led to upregulation of PDK2 mRNA and protein levels (Fig. S4B, C). The luciferase signal for *PDK2* promoter was increased significantly upon c-Myc expression, but the signal was significantly decreased when mutations were introduced in the *PDK2* promoter of the putative c-Myc-binding region (Fig. 3C). In addition, the chromatin immunoprecipitation (ChIP) assay demonstrated an enrichment of c-Myc at the PDK2 promoter, compared to the IgG control (Fig. 3D). Thus, these results suggest that c-Myc directly regulates PDK2 transcription in cells.

We next explored the effects of c-Myc-mediated PDK2 expression on cell proliferation and tumor growth. Depletion of c-Myc repressed cell proliferation in HT-29 and HCT116 cells, but had no impact on NCM460 cells (Fig. 3E and S4D). Reconstituted expression of PDK2 in c-Myc-depleted HT-29 and HCT116 cells rescued cell proliferation (Fig. 3E). To further evaluate the effect on tumor growth, we generated stable HT-29 cell lines with depletion of endogenous c-Myc or PDK2, and reconstituted expression of PDK2 in c-Myc-depleted cells (Fig. S4E). We subcutaneously injected these cell lines into nude mice. The c-Myc or PDK2 depletion similarly inhibited tumor growth and reduced Ki-67 expression in tumors (Fig. 3F–I). The inhibitory effect of c-Myc was abrogated by the reconstituted expression of PDK2 (Fig. 3F–I). Together, these results indicated that c-Myc impacts colorectal cancer cell proliferation and tumor growth via regulating PDK2.

### O-GlcNAcylation of c-Myc promotes its stability

**Fig. 4:**
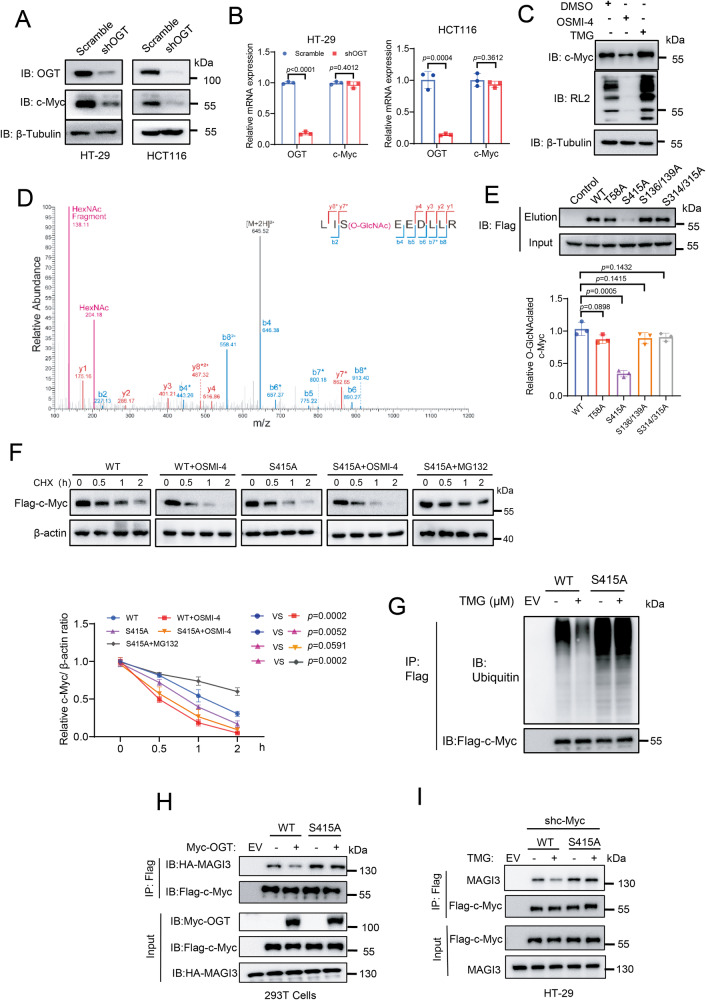
O-GlcNAcylation of c-Myc promotes its stability. Immunoblotting analysis (**A**) and quantitative PCR analysis (**B**) of c-Myc expression in HT-29 and HCT116 cells expressing scramble or shOGT. *n* = 3; Data are presented as means ± SD. *P* values were determined by unpaired two-tailed Student’s *t* tests. **C** Analysis of c-Myc O-GlcNAcylation upon OSMI-4 or TMG treatment. **D** Mapping the site of O-GlcNAcylation on c-Myc using mass spectrometry. **E** Probing the major site of glycosylation on c-Myc using various site-directed mutants. Quantification was shown. *n* = 3; Data are presented as means ± SD. Statistical analyses were performed by unpaired two-tailed Student’s *t* tests. **F** Immunoblotting of c-Myc levels in WT or S415A c-Myc-reconstituted HT-29 cells by CHX treatment in the presence of inhibitors for OGT (OSMI-4) or proteasome (MG132). Quantification was shown. *n* = 3; Data are presented as means ± SD. *P* values were determined by unpaired two-tailed Student’s *t* tests. **G** Immunoblotting of c-Myc ubiquitination levels in the presence or absence of TMG treatment in WT or S415A c-Myc-reconstituted HT-29 cells. EV, expression of vehicles as a negative control. **H** Analysis of c-Myc-MAGI3 interaction in 293 T cells overexpressing WT or S415A Flag-tagged c-Myc and HA tag MAGI3 in the presence or absence of OGT overexpression. Immunoblot analyses were performed with the indicated antibodies. **I** Analysis of c-Myc-MAGI3 interaction in WT or S415A c-Myc-reconstituted HT-29 cells. Immunoblot analyses were performed with the indicated antibodies.

Gene set enrichment analysis (GSEA) of the transcriptomic data of HT-29 cells showed that OGT depletion markedly reduced enrichment of c-Myc-targeted signaling pathways compared to the control (Fig. S5A). OGT depletion also decreased the expression levels of c-Myc-targeted glycolytic genes including HK2, PFKM, ENO1 and LDHB (Fig. S5B). In addition, loss of OGT reduced the production of lactate, NADPH and GSH, which were fully rescued by reconstituted expression of c-Myc, suggesting that OGT-mediated regulation of glucose metabolism in colorectal cancer cells is mainly dependent on c-Myc (Fig. S5C–E). We next asked whether OGT regulates c-Myc expression. To test this, we depleted OGT in both HT-29 and HCT116 cells, and observed that c-Myc protein expression, but not mRNA expression, was significantly decreased compared to the control, suggesting that OGT regulates c-Myc at the protein level (Fig. 4A, B). Overexpression of OGT in both HT-29 and HCT116 cells enhanced c-Myc protein expression, but not mRNA expression (Fig. S5F–H). In addition, inhibition of OGT with OSMI-4 decreased c-Myc protein expression in a dose-dependent manner (Fig. S5I). In contrast, inhibition of OGA (the enzyme to remove O-GlcNAc) with a small molecule ThiaMet G (TMG) to increase cellular O-GlcNAc levels in turn enhanced c-Myc protein expression (Fig. 4C). The mRNA level of c-Myc was not altered upon TMG or OSMI4 treatment (Fig. S5J). Thus, c-Myc protein levels are positively regulated by O-GlcNAc levels in cells.

The previous study showed that c-Myc was O-GlcNAc modified at Thr58 in lymphomas [40]. We speculate that c-Myc protein level is regulated by its O-GlcNAc modification. To investigate this, we firstly detected O-GlcNAc modification on c-Myc with a well-established chemoenzymatic labeling method. Flag-tagged c-Myc was ectopically expressed in HT-29 cells. Glycoproteins from cell lysates were enzymatically tagged with an azido-N-acetylgalatosamine (GalNAz), and subsequently reacted with alkynyl-biotin via Cu(I)-mediated azide-alkyne cycloaddition. After capture with streptavidin-coated beads, the proteins were eluted and further immunoblotted with the anti-Flag antibody. The blotting signal was clearly observed in the experimental group, but absent in the control group where the labeling enzyme was omitted. Treatment with TMG increased, while OSMI4 reduced, the blotting signal, confirming the presence of O-GlcNAc modification on c-Myc (Fig. S6A). However, the T58A (Thr to Ala mutation) mutant, which is glycosylation deficient, had a comparable blotting signal as the wild-type (WT) protein in the absence or presence of TMG treatment, indicating that T58 is not the glycosylation site on c-Myc (Fig. S6B). To further validate this result, we detected the effect of modulating O-GlcNAcylation on T58 phosphorylation, as T58 was known to be phosphorylated by GSK-3β and subjected to degradation mediated by the FBXW7 E3 ligase [39]. The data showed that treatment with TMG or OSMI4 had no apparent effect on T58 phosphorylation, nor the interaction of c-Myc with FBXW7 (Fig. S6C, D). The data suggest that O-GlcNAcylation on c-Myc is independent of T58 phosphorylation, thus further supporting the notion that T58 was not the glycosylation site in colorectal cancer cells. Thus, the absence of T58 glycosylation of c-Myc may reflect the cell-type specificity of O-GlcNAcylation. The underlying mechanisms for differential glycosylation await further investigation.

We next mapped the sites of O-GlcNAc on c-Myc in HT-29 cells with mass spectrometry. We identified five putative glycosylation sites (S316, S319, S314, S315 and S415) (Fig. 4D). Based on this result, we further generated different point mutations of c-Myc and detected their glycosylation levels. Notably, only S415A mutant, but not other mutants, exhibited a marked reduction of glycosylation signal (Fig. 4E). Consistently, ectopic expression of OGT in HT-29 cells increased the glycosylation level of WT, but not S415A c-Myc (Fig. S6E). Besides, S415 of c-Myc is evolutionarily conserved across species (Fig. S6F). Together, these results suggest that S415 is the major site of glycosylation of c-Myc.

We next explored the effect of S415 glycosylation on c-Myc expression. When incubated with cycloheximide (CHX) to repress new protein synthesis, c-Myc showed a faster degradation rate in HT-29 cells upon OGT inhibition compared with the control. The degradation was recovered by addition of the proteasomal inhibitor MG132, but not the lysosomal inhibitor chloroquine, suggesting that O-GlcNAcylation regulates c-Myc degradation via the proteosome-dependent pathway (Fig. S6G). Moreover, S415A c-Myc exhibited enhanced degradation rate than WT c-Myc, and this effect could not be further potentiated with OSMI-4 treatment (Fig. 4F). Similar results were observed in HCT116 cells (Fig. S6H). Thus, these results suggest that S415 glycosylation promotes c-Myc stability in colorectal cancer cells. As glycosylation regulates c-Myc degradation via the proteosome-dependent pathway, we further detected the ubiquitination level of c-Myc in HT-29 cells. As expected, TMG treatment significantly inhibited the ubiquitination of WT c-Myc, but showed little impact on S415A c-Myc (Fig. 4G).

MAGI3 was a principal E3 ubiquitin ligase for c-Myc in colorectal cancer cells [46]. Consistently, loss of MAGI3 in HT-29 cells increased c-Myc expression and concomitantly decreased ubiquitination of c-Myc (Fig. S7A, B). On the contrary, MAGI3 overexpression reduced the expression of c-Myc and increased the level of c-Myc ubiquitination, confirming that MAGI3 is a major E3 ubiquitin ligase for c-Myc degradation in colorectal cancer cells (Fig. S7C, D). As the PDZ domain binding motif (PBM) at the C-terminus of c-Myc is required for the association of c-Myc with MAGI3, we speculate that S415 glycosylation may affect the binding of c-Myc with MAGI3 [46]. To test this, we ectopically expressed Flag-tagged WT or S415A c-Myc and HA-tagged MAGI3 in 293 T cells with or without OGT overexpression. Co-immunoprecipitation (Co-IP) assays showed that OGT overexpression significantly reduced the interaction of WT c-Myc with MAGI3, but had no effect on the interaction of S415 A c-Myc and MAGI3 (Fig. 4H). Co-IP in the reverse order also showed consistent results (Fig. S7E). We then analyzed the effect of O-GlcNAcylation on the interaction of the endogenous MAGI3 in WT or S415A c-Myc reconstituted HT-29 cells. As shown in Fig. 4I, elevated O-GlcNAcylation by TMG treatment weakened the association of c-Myc with MAGI3 in cells expressing WT c-Myc, whereas no effect was observed in cells expressing S415A c-Myc. Together, these results suggest that S415 glycosylation promotes c-Myc expression by inhibiting its degradation via blocking the interaction with MAGI3.

### S415 glycosylation of c-Myc regulates PDK2 transcription and metabolism

**Fig. 5:**
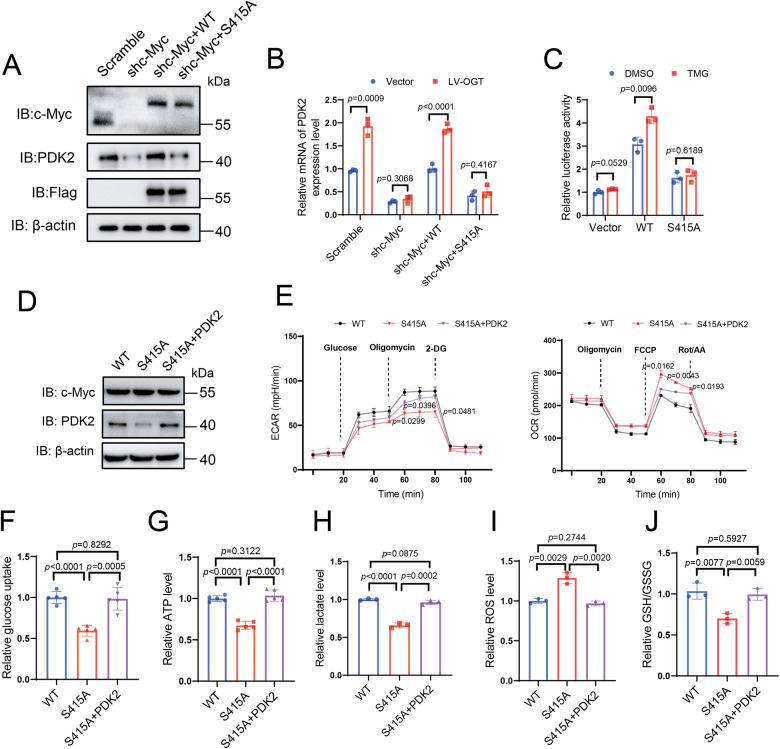
S415 glycosylation of c-Myc regulates PDK2 transcription and metabolism. **A** Generation of stable HT-29 cells with c-Myc knockdown and reconstituted expression of shRNA-resistant WT or S415A c-Myc. The c-Myc knockdown efficiency and re-expression were examined using immunoblotting. **B** Quantitative PCR analysis was performed to detect the expression of the PDK2 in HT-29 cells with c-Myc knockdown and reconstituted expression of shRNA-resistant WT and S415A c-Myc in the presence or absence of OGT overexpression. *n* = 3; Data are presented as means ± SD. *P* values were determined by unpaired two-tailed Student’s *t* tests. **C** Dual-luciferase reporter assay showing the effects of c-Myc WT and S415A on relative PDK2-promoter activity in the presence or absence of TMG in the 293 T cells. *n* = 3; Data are presented as means ± SD. *P* values were determined by unpaired two-tailed Student’s *t* tests. **D** Immunoblotting of c-Myc and PDK2 expression in stable HT-29 cells ectopically expressing Flag-tagged WT or S415A c-Myc with PDK2 overexpression, with a simultaneous depletion of the endogenous c-Myc. **E** ECAR and OCR in HT-29 cells stably expressing scramble shRNA, c-Myc-targeting shRNA and shRNA-resistant WT, S415A c-Myc or S415A c-Myc with PDK2 overexpression. *n* = 3; Data are presented as means ± SD. *P* values were determined by unpaired two-tailed Student’s *t* tests. Comparison of glucose uptake (**F**), ATP(**G**), lactate (**H**), GSH/GSSG (**I**), ROS (**J**) in HT-29 cells reconstituted with WT, S415A c-Myc or S415A c-Myc with PDK2 overexpression. *n* = 3; Data are presented as means ± SD. *P* values were determined by unpaired two-tailed Student’s *t* tests.

We further probed the O-GlcNAcylation levels of c-Myc in different cell lines. We observed that c-Myc glycosylation levels were significantly higher in RKO, SW620, HT-29 and HCT116 cells compared to NCM460 cells (Fig. S8A). To investigate the functional role of c-Myc glycosylation, we depleted endogenous c-Myc with shRNA and simultaneously expressed Flag-tagged WT or S415A c-Myc, which was resistant to the shRNA targeting (Fig. 5A). Depletion of c-Myc reduced PDK2 mRNA and protein expression, which was effectively rescued by the re-expression of WT, but not S415A c-Myc (Fig. S8B). Overexpression of OGT elevated PDK2 mRNA levels in WT rescued cells, but not in S415A rescued cells (Fig. 5B). Depletion of c-Myc abolished the upregulation of PDK2 mRNA mediated by OGT overexpression in both HT-29 and HCT116 cells (Fig. S8C). Moreover, ChIP assays showed that enrichment of c-Myc at the PDK2 promoter was significantly reduced for S415A c-Myc as compared to WT c-Myc (Fig. S8D). TMG treatment potentiated the luciferase signal in WT c-Myc reconstituted HT-29 cells, but not in S415A c-Myc reconstituted cells (Fig. 5C). Together, these results indicate that c-Myc regulates PDK2 mRNA expression via S415 glycosylation.

To further verify that the effects of c-Myc glycosylation were exerted through PDK2, we restored PDK2 expression in S415A c-Myc reconstituted HT-29 cells (Fig. 5D). Cells expressing S415A c-Myc showed reduced ECAR and increased OCR compared to cells expressing WT c-Myc. The effects were abolished by restoration of PDK2 expression in S415A c-Myc reconstituted cells, indicating that c-Myc S415 glycosylation regulated glucose metabolism via PDK2 (Fig. 5E). Consistently, re-expression of S415A c-Myc in HT-29 cells attenuated glucose uptake, lactate and ATP production, while elevating the level of ROS and reducing the ratio of GSH/ GSSG. Notably, these effects were reversed by restoring PDK2 expression in S415A c-Myc reconstituted cells (Fig. 5F–J). Collectively, these results indicate that O-GlcNAcylation of c-Myc on S415 regulates PDK2 transcription and the subsequent metabolic activity.

### c-Myc-mediated PDK2 expression is vital for cell proliferation and tumor growth

**Fig. 6:**
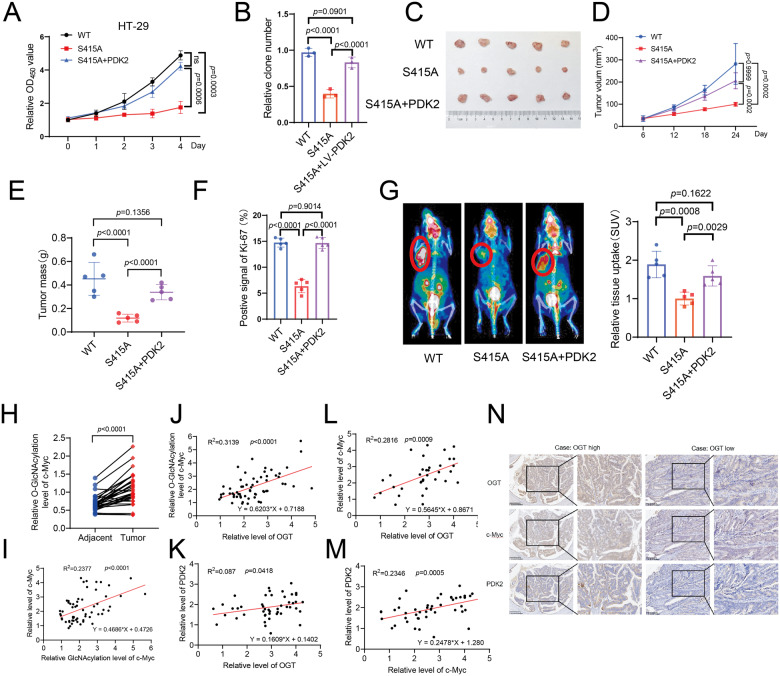
c-Myc-mediated PDK2 expression is vital for cell proliferation and tumor growth. Cell proliferation (**A**) and clone formation (**B**) of HT-29 cells stably expressing scramble shRNA, c-Myc-targeting shRNA, or reconstituted with WT, S415A c-Myc or S415A c-Myc with PDK2 overexpression. **C** Xenograft analysis of HT-29 cells stably expressing scramble, c-Myc-targeting shRNA, or reconstituted with WT, S415A c-Myc or S415A c-Myc with PDK2 overexpression. Images of dissected tumors from mice. Analysis of tumor growth (**D**), tumor weights (**E**) and Ki67 staining (**F**) of tumors generated from above HT-29 cells. Scale bars: 100 μm. Quantification was shown. *n* = 5; Data are presented as means ± SD. *P* values were determined by unpaired two-tailed Student’s *t* tests. **G** Representative ^18^F-FDG micro-PET/CT images of mice. Red circles indicate glucose uptake in tumors. *n* = 5 mice per group. Data are presented as means ± SD. *P* values were determined by unpaired two-tailed Student’s *t* tests. **H** The statistical analysis of c-Myc glycosylation in paired tumor and matched adjacent tissues. *n* = 30; Data are presented as means ± SD. *P* values were determined by unpaired two-tailed Student’s *t* tests. **I** Analysis of the correlation of c-Myc O-GlcNAcylation and c-Myc expression levels by western blotting. *n* = 30; Data are presented as means ± SD. *P* values were determined by unpaired two-tailed Student’s *t* tests. **J** Analysis of the correlation of OGT expression and c-Myc O-GlcNAcylation by western blotting. **K**–**M** Analysis of the correlation of OGT, c-Myc and PDK2 protein levels in clinical CRC specimens. Analysis of the correlation of OGT / PDK2 (**K**), OGT / c-Myc (**L**), and c-Myc / PDK2 (**M**) from CRC samples. Representative IHC staining of OGT and c-Myc in 30-paired cases of CRC samples (**N**). Scale bars: 200 μm. Right panels are ×4 magnification of the dashed areas on the left. *P* values were determined by unpaired two-tailed Student’s *t* tests.

We next examined the effect of c-Myc S415 glycosylation on cell proliferation. As expected, S415A c-Myc reconstituted HT-29 cells displayed a reduction in cell proliferation rate and colony formation ability as compared to WT c-Myc reconstituted cells. These effects were reversed by restoring PDK2 expression in S415A c-Myc reconstituted cells (Fig. 6A, B and S9A). To examine the effect of c-Myc glycosylation-regulated PDK2 expression in tumor development, we subcutaneously injected the above cell lines into nude mice. Mice bearing S415A c-Myc reconstituted cells showed a significantly reduced tumor volume, weight and Ki-67 expression compared to mice bearing WT c-Myc reconstituted cells (Fig. 6C–F). These inhibitory effects were abrogated by restoring PDK2 expression in S415A c-Myc reconstituted cells (Fig. 6C–F). To further verify the role of c-Myc glycosylation in glucose metabolism, we employed 18F-FDG PET/CT scans to analyze the glucose uptake in tumors. The results show that mice bearing S415A c-Myc reconstituted cells showed a marked decrease in 18F-FDG uptake compared to mice bearing WT c-Myc reconstituted cells (Fig. 6G). Restoring PDK2 expression in S415A c-Myc reconstituted cells fully rescued glucose uptake in tumors (Fig. 6G). Collectively, these results demonstrate that c-Myc glycosylation-regulated PDK2 expression promotes glucose metabolism and tumor growth.

To detect the clinical importance of c-Myc O-GlcNAcylation, we performed western blotting and immunohistochemistry (IHC) analyses of 30 pairs of colorectal cancer tissues and matching peritumoral tissues. We observed that the levels of c-Myc, PDK2 and c-Myc glycosylation were all significantly higher in tumor tissues compared to the matching peritumoral tissues (Fig. 6H, S9B–D). In addition, the levels of c-Myc glycosylation were positively correlated with protein levels of OGT and c-Myc (Figs. 6I, J). IHC analyses showed that OGT levels were positively correlated with c-Myc levels, both of which were positively correlated with PDK2 levels, in line with the analysis of the CRC dataset based on the TCGA database (Fig. 6K–N, S9E, F). Kaplan-Meier analysis in TCGA database revealed that PDK2 mRNA expression levels were positively correlated with poor survival of patients with colorectal cancer (Fig. S9G). Collectively, these results support the cellular data, and underscore the importance of the OGT–c-Myc–PDK2 axis in colorectal cancer development.

## Discussion

**Fig. 7:**
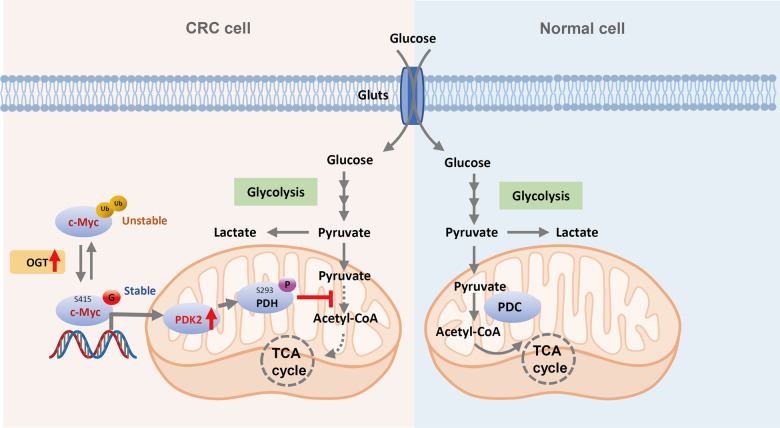
A schematic model showing c-Myc O-GlcNAcylation regulates the TCA cycle and tumor growth in CRC. S415 O-GlcNAcylation represses the degradation of c-Myc. Thus, c-Myc O-GlcNAcylation facilitates the Warburg effect and results in colorectal cancer tumorigenesis.

Colorectal cancer cells are highly glycolysis-dependent, with attenuated TCA cycle metabolism [1]. The altered glucose metabolism provides the metabolic requirements for rapid macromolecule synthesis, and coordinates cellular redox homeostasis, thereby fueling tumor growth [5]. However, despite the accumulated findings on the regulation of glycolysis, how the TCA cycle is modulated in cancer cells remains elusive. Here, we identified a novel regulatory mechanism of the TCA cycle in colorectal cancer cells. We demonstrate that O-GlcNAcylation represses the TCA cycle by enhancing PDK2 expression. Mechanistically, OGT-mediated O-GlcNAcylation directly modifies c-Myc on S415 to inhibit ubiquitination and proteasomal degradation of c-Myc by impeding the interaction between c-Myc and the E3 ligase MAGI3. Accumulated c-Myc can bind to the promoter region of PDK2 to upregulate the transcriptional expression of PDK2. c-Myc glycosylation-mediated expression of PDK2 dampens the mitochondrial pyruvate metabolism and ROS production, leading to enhanced cell proliferation and tumor growth in mice (Fig. 7). Thus, our study identifies OGT–c-Myc–PDK2 axis as a key mechanism to regulate colorectal cancer metabolism.

c-Myc is a well-studied transcription factor that modulates the expression of genes related to cell proliferation, apoptosis and metabolism [27, 29]. Given the growth-promoting function of c-Myc, the intricate regulation of c-Myc abundance plays critical roles in normal cellular physiology. Aberrant expression of c-Myc is implicated in tumorigenesis. The tactics to tumor treatment by targeting c-Myc should be carefully performed due to its indispensable role in cellular homeostasis. Elevated c-Myc O-GlcNAcylation in cancer cells emerges as a promising avenue for therapeutic intervention. While accumulated studies have revealed that upregulation of c-Myc elevated the expression of glycolysis-related genes, contributing to aerobic glycolysis and tumor progression, how c-Myc rewires the TCA cycle in tumors is largely unknown [30, 46]. Understanding of the metabolic vulnerabilities conferred by glycosylation-mediated c-Myc stabilization paves the way for combination therapies that target both oncogenic signaling and metabolic dependencies in cancer cells. Thus our study not only enhances our understanding of cancer biology but also suggests new therapeutic strategies in cancer.

O-GlcNAcylation is highly sensitive to fluctuations in nutrient levels and it is regarded as a nutrient sensor in cells [22, 47, 48]. Increasing evidence has revealed that alteration in nutrition metabolism can lead to aberrant O-GlcNAcylation, which directly modifies metabolic enzymes such as PGK1, PFK1, G6PD, PKM2 and MDH1 to reprogram metabolic pathways, thereby contributing to tumor growth [44, 49–52]. In this study, we identify a novel O-GlcNAcylation site on c-Myc in colorectal cancer cells, indicating that different glycosylation sites may be present in the same protein in different cells. This may reflect the existence of differential modes of regulation in different tissues of origin under unique nutrient and stress conditions.

### Supplementary information

Supporting Information Text and Figures

Supplementary Table

Supplement Material-uncropped western blots

## Data Availability

All study data are included in the article and Supplementary information. Data supporting the findings of this study are available on reasonable request from the corresponding author. The RNA sequencing data generated in this study have been submitted to the NCBI Gene Expression Omnibus (GEO) datasets with accession number GSE232258. The survival analyses of LIHC and CRC patients were analyzed by using GEPIA2 (http://gepia2.cancer-pku.cn). The OGT expression analyses of STAD, LUAD, LIHC, PRAD and CRC patients were performed based on cBioPortal for Cancer Genomics.
