# Supplementary material for: The OGT–c-Myc–PDK2 axis rewires the TCA cycle and promotes colorectal tumor growth
Source: Cell Death Differ. 2024 May 22;31(9):1157–69. doi: 10.1038/s41418-024-01315-4 (PMC11369260; doi:10.1038/s41418-024-01315-4)
Supplement: Supplementary file 1 — Supporting Information Text and Figures [file 41418_2024_1315_MOESM1_ESM.pdf]

Materials and Methods

Cell culture

Cells obtained from American Type Cell Culture (ATCC) included NCM460, HT-29, HCT116, RKO, SW620, LoVo, and 293T. All cells were cultured in complete medium (10% fetal bovine serum was added to Dulbecco's Modified Eagle's Medium) at 37°C with 5% CO_2. STR profiling was used to identify every cell line, and no mycoplasma contamination was found.

Western blot analysis

Protease inhibitor cocktail (Yeasen, 20124ES03) and 20 μM Thiamet-G (MedChemExpress, HY-12588) were added to RIPA lysis buffer (Beyotime, P0013C) to lyse the cells. After being resolved on a 10% SDS-PAGE gel, about 40 μg of protein lysate were transferred to PVDF membranes (Millipore, IPVH00010). The membrane was blocked with 5% skim milk for one hour at room temperature, and then the matching primary antibodies were incubated overnight at 4 °C. The proteins were visualized by chemiluminescence with the ECL reagent. **Supplementary Table 1** contains a list of the antibodies utilized in this investigation. The full and uncropped western blots were present in the **supplementary data**. The intensity of protein bands was measured with Image J.

Immunoprecipitation (IP) and Co-immunoprecipitation (Co-IP)

Protease and phosphatase inhibitors were added to IP buffer before cells were lysed

(Yeasen, 20124ES03). The lysates were mixed with protein A/G agarose (MedChemExpress, HY-K0202-1) following incubation with the designated antibodies. For proteins with a tag, we used immunomagnetic beads coupled with anti-Flag/anti-HA antibody. After IP, protein A/G agarose or magnetic beads were washed three times with TBST (0.1% Tween-20, 150 mM NaCl, 10 mM Tris-HCl pH7.5) and eluted in SDS lysis buffer (100 mM NaCl, 1%SDS, 50 mM Tris-HCl pH 7.5) for western blotting analyses.

Quantitative RT-PCR

Trizol reagent (Takara, 9109) was used to extract total RNA. The quantity and quality of RNA were measured using a Thermo Fisher Scientific NanoDrop 2000 spectrophotometer. Following the manufacturer's instructions, Superscript reverse transcriptase (TAKARA, DRR047S) was used to reverse transcribe RNA. cDNA, gene-specific primers, and SYRB Green Master Mix were used in quantitative real-time PCR (yesean, 11201ES08), which was detected using the Bio-Rad CFX96TM Real-Time System. The primers used in this work are presented in **Supplementary Table 2**. Fold changes were quantified using the 2-rCt method and normalized to β-actin.

Analysis of c-Myc glycosylation

Chemoenzymatic labeling was used to identify the O-GlcNAcylation of the protein. Cell lysate (500 μg) was conjugated with an alkyne-biotin compound utilizing the Click-iT Protein Analysis Detection Kit (Invitrogen) after being labeled with an azide

group using β-1,4-galactosyltransferase GalT (Y289L). Control experiments were conducted simultaneously in the absence of UDP-GalNAz or GalT. After neutralizing biotinylated lysates in a buffer (150 mM NaCl, 100 mM Na_2HPO_4, and 6% NP-40), the samples were treated with Pierce streptavidin resin for an overnight period at 4 °C. The resin was washed three times with low salt buffer (0.1 M Na_2HPO_4,0.15 M NaCl,0.1% SDS,1% Triton-X100,0.5% sodium deoxycholate, pH 7.5) and high salt buffer (0.1 M Na_2HPO_4, 0.5 M NaCl, 0.2% Triton-X100, pH 7.5), respectively. The loading buffer was used to elute the bound proteins for western blotting analyses.

Site mapping of c-Myc glycosylation

c-Myc was isolated from HT-29 cells that were coexpressing Flag-tagged c-Myc and HA-tagged OGT. The protein was separated using 10% SDS/PAGE and stained with Coomassie blue R250 (Bio-Rad). The c-Myc band was excised, rinsed with Milli-Q water, and destaining buffer was applied twice for 30 minutes. After being dehydrated in acetonitrile and rehydrated with 10 mM DTT in 50 mM IAM for 45 minutes at 56 °C, proteins in-gel were digested with Trypsin/GluC for 16 hours at 37 °C. As directed by the manufacturer, the resulting peptides were desalted using Millipore's C18 Zip-Tips. Following vaporation in a vacuum centrifuge, the samples were reconstituted in water and subjected to LC-MS/MS analysis. The materials were identified using the ETD fragmentation mode of the Thermo Fisher Scientific Orbitrap Elite mass spectrometer.

Analysis of metabolite by HPLC-MS/MS

Cells were collected and resuspended with prechilled 80% methanol by well vortex. Then samples were incubated on ice for 5 min and centrifuged at 15,000 g, 4 °C for 15 min. Some of supernatant was diluted to final concentration containing 53% methanol LC-MS grade water. Cellular metabolites were quantitated by LC-MS/MS with a triple quadrupole mass spectrometer (the QTRAP 6500+ System, ABSCIEX). Briefly, metabolites were separated chromatographically on a SeQuant Zic-pHILIC column (5 μm polymer 150 × 2.1 mm, Millipore Sigma) using a high-performance UHPLC system (Exion LC AD system) coupled to a triple quadrupole mass spectrometer (QTRAP 6500+ System, AB SCIEX). The eluents were eluent A (0.1% Formic acid-water) and eluent B (0.1% Formic acid-acetonitrile). The solvent gradient was set as follows: 2% B, 2 min; 2-100% B, 15.0 min; 100% B, 17.0 min; 100-2% B, 17.1 min; 2% B, 20 min. The following gradient was employed: 0.01 min 80% B, 20 min 20% B, 20.5 min 80% B, 34 min 80% B. Metabolites were detected by MRM transitions in positive or negative modes. The raw data were extracted with the software Analyst v1.7.2 and OS v1.7.

RNA-seq analysis

We used the following publicly available gene expression datasets: GSE232258. Novogene carried out RNA-seq. Briefly stated, Trizol Reagent (Takara, 9109) was used to extract the total RNA from HT-29 cells following Scramble or OGT depletion, respectively. Agilent Technologies, CA, USA's Bioanalyzer 2100 system's RNA Nano 6000 Assay Kit was utilized to assess the overall amounts and quality of RNA. The

RNA-Seq libraries were made with the Agilent SureSelect Strand-Specific RNA Library Preparation Kit. Next, AMPure XP Beads (Beckman Coulter, Brea, CA, USA) were used to select the library sizes. The libraries were then pooled based on the target amount of data off the machine and the effective concentration, and the Illumina NovaSeq 6000 was used to sequence the data.

ECAR and OCR analysis

The Glycolysis Stress Test Kit (Angilent, 103020-100) was used to calculate ECAR based on the manufacturer's instructions. In summary, 1.0×10^4 cells were seeded into an XF96 plate overnight. After switching to XF medium, the media were incubated for one hour. To detect the ECAR, more glucose (10 mM), oligomycin (1 μM), and 2-deoxy glucose (2-DG) (100 mM) were added. The Cell Mito Stress Test Kit (Angilent, 103015-100) was used to determine OCR. To detect the OCR, 1 μM rotenone, 0.5 μM FCCP, and 1 μM oligomycin were automatically added into the cartridge. The Seahorse Bioscience XF96 Extracellular Flux Analyzer was used to determine and normalize OCR and ECAR values to cell counts.

Measurement of Intracellular ROS Levels

Dichlorofluorescein diacetate (DCFH-DA), a fluorescent dye (Beyotime, S0033S), was used to detect the levels of ROS. DCFH-DA was diluted 1:1000 to a final concentration of 10 mmol/L in serum-free medium. The cells were collected and suspended in diluted DCFH-DA at a concentration of 1 to 20 million/ml and cultivated for 20 minutes at

37ºC in a cell incubator. Every three to five minutes, flip and stir to ensure the probe is in complete contact with the cells. To ensure that no DCFH-DA entered the cells, the cells were carefully washed three times using serum-free cell culture media. A Multi-Mode Plate Reader (BioTek) with an excitation wavelength of 488 nm and an emission wavelength of 525 nm was used to measure the fluorescence intensity.

Measurement of intracellular or tissue NADPH and $NADP^+$ levels

To measure NADPH and $NADP^+$ levels, the $NADP^+$/NADPPH Quantitation Kit (Biovision, K347-100) was utilized. Briefly, Cells were lysed in extraction buffer at 4°C for 20 minutes and centrifuged at 12,000 g for 10 minutes. To analyze total $NADP^+$/NADPH, 50 µL supernatant was added to 100 µL $NADP^+$ reaction mixture and incubated at room temperature for 30 minutes. After being recorded, the signal at 450 nm was adjusted for protein concentration.Following the elimination of $NADP^+$, NADPH levels were measured as previously mentioned, by heating for 30 minutes at 60°C.

Measurement of intracellular or tissue GSH and GSSG levels

Using a GSH/GSSG Quantitation Kit (Biovision, K264) and following the manufacturer's instructions, the levels of GSH and GSSG were determined. After adding the cell to the GSH Assay Mixture, it remained at room temperature for half an hour. A Multi-Mode Plate Reader (BioTek) was used to detect the absorbance at 412 nm and then normalized to the concentration of protein.

Generation of c-Myc reconstituted stable cell lines

To generate c-Myc reconstituted cell lines, pLenti-FlagN-shRNA vector that affords small hairpin RNA (shRNA)-mediated knockdown of endogenous c-Myc while co-expressing exogenous Flag-tagged c-Myc (WT, S415A) was employed. To deplete endogenous c-Myc, the 3′ UTR sequence of c-Myc gene was inserted into pLenti-FlagN-shRNA vector. The rescued c-Myc sequences (flag-tagged WT, S415A) were inserted into the vector. The sequences were present in **Supplementary Table 2**. Cells were infected with the lentiviruses and stable cell lines were selected with 4 μg/ml puromycin for 2 weeks.

Measurement of glucose uptake, ATP levels, lactate production,

Glucose uptake was assessed using the glucose uptake-GloTM test kit (Promega, J1341) following the manufacturer's instructions. Briefly, 2,000 cells were seeded per well in a 96-well plate. Cells were then washed with PBS. 50 μl of prepared 1mM 2DG was added to each well and incubated at room temperature for 10 minutes. 25 μl Stop Buffer was added to terminate the reaction. Followed by the addition of 25 μl Neutralization Buffer, 100 μl 2DG6P Detection Reagent was added and incubated at room temperature for 0.5-5 hours. The signal integration time was selected as 0.3-1 SEC on the luminescence detector (BioTek), and the luminescence signal was recorded.

ATP levels were determined by ATP assay kit (Beyotime, S0026). Briefly, cells were lysed and centrifuged at 12,000 g for 5 minutes at 4°C.The supernatant was with

incubated with100 microliters of the ATP test solution for five minutes at room temperature. the RLU values were measured using a luminometer (BioTek).

To determine the amount of lactate released by cells, the culture media was collected. A lactate test kit (Solarbio, BC2235) was used to measure lactate levels in accordance with the manufacturer's instructions.The extraction solution to volume (ml) ratio is 500~1000:1 (it is advised that 5 million cells be added to 1ml of extraction solution); After centrifuging for 10 minutes at 4°C and 12000g, supernatant was removed of 0.8 ml , then 0.15 mL of extract solution 2 was gradually added and mixed. The absorbance at 570 nm was recorded using a microplate reader (BioTek).

Cell proliferation assay

Cell proliferation rate was annlyzed by Cell Counting Kit-8 (Beyotime, C0039). In a 96-well plate, 2000 cells were planted and cultivated for the specified amount of time. After adding CCK-8 solution to each well and incubating for one hour at 37 °C, the Multi-Mode Plate Reader (BioTek) was used to measure the absorbance at 450 nm.

Colony formation assay

Lentivarus was used to infect HT-29, HCT116, and NCM460 cells. Then 1000 cells were seeded into each well of six-well plates, and the cells were grown for two weeks at 37 °C in new media. After 30 minutes of fixing in 4% paraformaldehyde (Beyotime, P0099), the cells were stained for 20 minutes with 1% crystal violet (Beyotime, C0121).

Determination of c-Myc half-life

To repress the synthesis of new proteins, 50 μM cycloheximide (CHX) (MedChemExpress, HY-13259) was added to c-Myc WT or S415A rescue HT-29 cells. The stated time points (0, 0.5, 1 or 2 hours) were used to harvest the cells after treatment. After that, c-Myc levels were analyzed by western blotting, and the relative half-life was calculated .

Dual luciferase reporter assays

Using the primers listed in **Supplementary Table1**, the upstream (-1498/+1) region of the PDK2 gene promoter was cloned into the pGL3-basic expression vector. Using Polyjet (Sinagen, SL100688), cells were transfected with pGL3 and pGL3-PDK2 expression vectors. Renilla luciferase expression vector (pRL) was also transfected into cells as a transfection control. After 48 h of transfection, luciferase assays were performed using a Dual Luciferase Reporter Assay System (Beyotime, RG088S) according to the manufacturer's instructions. The promoter activities were normalized to the corresponding values of Renilla luciferase.

Chromatin immunoprecipitation assay (ChIP)

Plvx-HA control or plvx-HA c-Myc WT/S415A vectors were transfected into HT-29 cells for a duration of 48 hours. The cells were collected and washed three times with PBS. 1% formaldehyde was added and incubated at room temperature for 10 minutes. The addition of 125 mM glycine then halted the process. The cells were collected by centrifugation at 300 × g at 4°C and then resuspended it in 1ml ChIP sonication buffer

(10 mM Tris-HCl pH 7.4, 25 mM KCl, 5 mM $MgCl_2$) and DNA was sheared by a sonicator to fragment size. Debris from pellet cells were removed by centrifuging at 14,000 × g for 15 minutes at 4 °C. 10% of the supernatant was set aside as input, and the remaining supernatant was incubated with an anti-c-Myc antibody overnight at 4 °C. The corresponding IgG was used as a negative control. The supernatant was mixed with Protein A/G Magnetic Beads and then incubated for 4 hours at 4 °C on a rotary homogenizer. Following that, the beads were washed using the following buffers: low salt buffer (0.1% SDS, 1.0% TritonX-100, 2 mM EDTA, 20 mM Tris-HCl pH 8.0, 150 mM Nacl), high salt buffer (0.1% SDS, 1.0% TritonX-100, 2 mM EDTA, 20 mM Tris-HCl pH 8.0, 500 mM Nacl), LiCl wash buffer. Using 300 μL of elution buffer, the sheared DNA was eluted. The products were treated with RNaseA and Proteinase K after the crosslinking was reversed by adding 5 M NaCl (final concentration, 0.2 M) and cubating at 65 °C for the entire night. Using a PCR purification kit (Qiagen, 28024), the immunoprecipitated DNA fragments were recovered and subjected to RT-PCR. The crosslinking was undone by adding 5 M NaCl (final concentration: 0.2 M) and letting it sit at 65 °C for an entire night. The products were then treated with RNaseA and Proteinase K. Quantitative RT-PCR was used to evaluate the immunoprecipitated DNA fragments after isolated using a PCR purification kit (Qiagen, 28024).

Gene set enrichment analysis

Based on log2-fold changes, we used the signal-to-noise measurement derived by GSEA to rank the genes according to their connection with the Scramble (n = 3) and shOGT (n = 3) groups. Gene sets related to the glucose metabolic process were gathered

from the following database: https://www.gsea-msigdb.org/gsea/index.jsp.

Transcription factors prediction

The putative transcription factors of PDK2 were scanned using the online bioinformatics tools TRANSFAC (http://gene-regulation.com/pub/databases.html) and Animal TFDBI (http://bioinfo.life.hust.edu.cn/ AnimalTFDB/). For every transcription factor, the sites that had the highest binding score were set aside. Two transcription factors that were chosen were evaluated for their binding scores using JASPAR (http://jaspar.genereg.net/).

Xenograft model in nude mice and PET/CT imaging

6-week-old male nude BALB/c mice (n = 5 per group) were randomly and blindly assigned to groups. 4×10^6 HT-29 cells were injected into mice flanks. Once a week, the tumors' volume was measured. When tumor diameter reached 400 mm^3, 0.15 mCi ^{18}F-FDG was injected intravenously after starvation for one night. After 1 hour, ^{18}F-FDG micro-PET-CT scanning (Siemens, Berlin, Germany) was conducted. A pseudocolor map was used to display the PET acquisition images, with red representing a significant absorption of ^{18}F-FDG. The ^{18}F-FDG-PET activity was measured using SUVmax and MTV. Tumor samples were gathered and weighed.

Immunohistochemistry staining

Tissue blocks impregnated in paraffin were placed on glass slides that were charged with polylysine. 30 minutes of exposure to 3.0% H_2O_2 inhibited endogenous peroxidase

activity. In a citrate buffer (pH 6.0), antigen retrieval was carried out for ten minutes at 100°C. After that, sections were incubated with anti-c-Myc (Servicebio), anti-OGT (Abcam), and anti-PDK2 (Abcam) for an entire night at 4 °C. Following washing, the sections were left at room temperature for two hours and incubated with the matching secondary antibodies. The avidin–biotin complex procedure was carried out using the Vecta-stain ABC kit (Vector labs) in accordance with the instructions. As isotype controls, sections were treated without primary antibodies with immunoglobulins that matched both isotype and concentration. The DAB Elite kit (K3465, DAKO) was used to visualize peroxidase activity, and brown tissue color indicated positive staining. Sections of tumor tissue fixed in paraffin (6 μm thick) were stained with a Ki-67 antibody. The Vecta-stain ABC kit (Vector Labs) was used to perform immunohistochemistry in accordance with the protocol.

Statistical analysis

Figure legends indicate that all measurements were made from separate samples, and no data were left out. The program GraphPad Prism 8.0 was used to analyze the data. The figure legend includes sample sizes. Data are presented as means ± SD. P-values were determined by unpaired two-tailed Student's t-tests. a value of $P < 0.05$ was considered statistically significant.

Lead contact

Further information and requests for reagents may be directed, and will be fulfilled by the Lead Contact, Wen Yi (wyi@zju.edu.cn).

Materials availability

This work did not produce any novel reagents.

Supplementary Figure

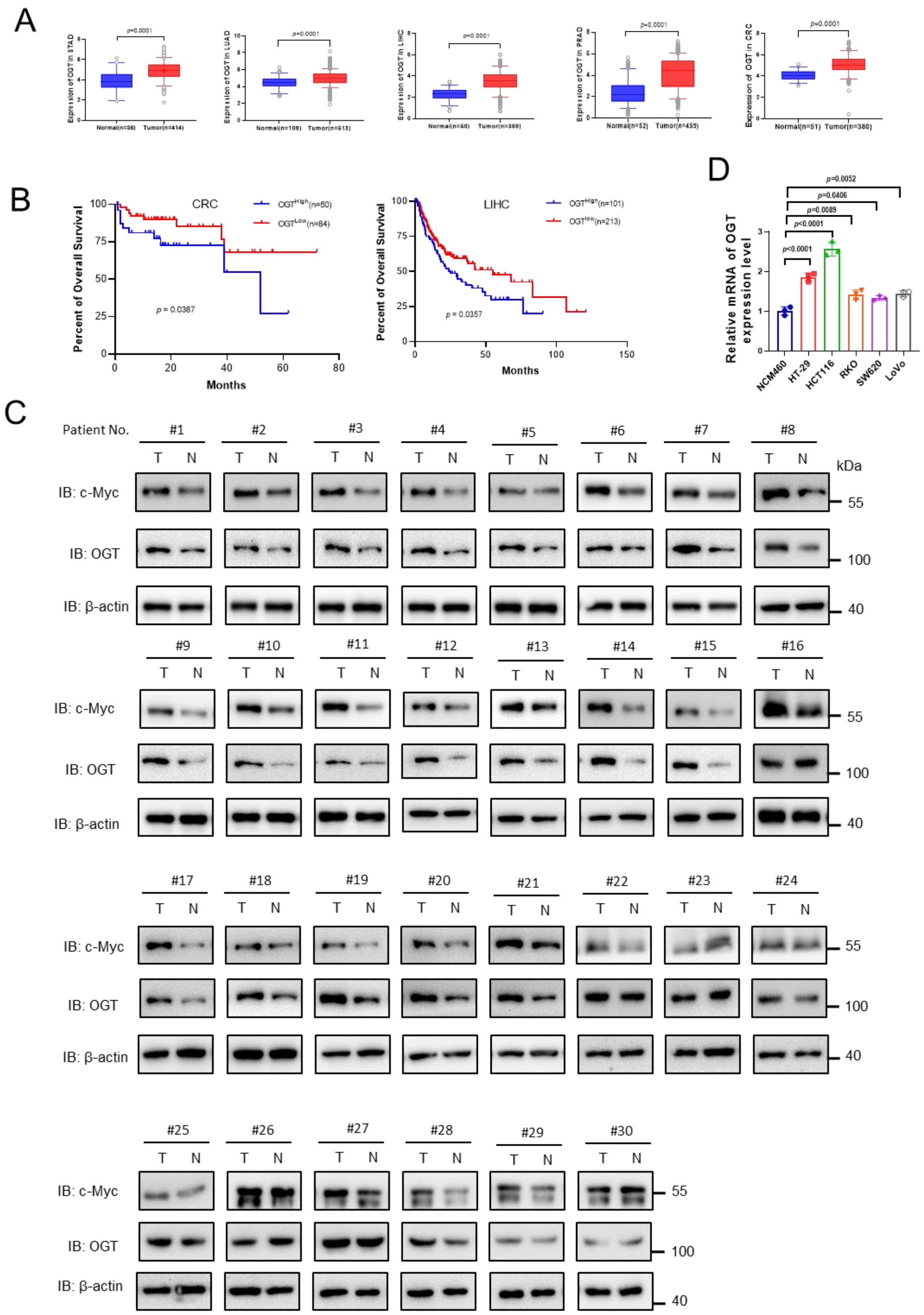

Figure S1

(A) The mRNA level of the OGT in the cancer tissues (CRC, STAD, LUAD, LIHC, PRAD) was higher than that matching peritumoral tissues. Gene expression data was downloaded from the TCGA database (https://www.cancer.gov/tcga/).

(B) Kaplan-Meier overall survival curves of patients with CRC of high (n = 50) and low (n = 84) OGT mRNA expressions (stratified by quartile) obtained from the TCGA database (log-rank test). Kaplan-Meier overall survival curves of patients with LIHC of high (n = 101) and low (n = 213) OGT mRNA expressions (stratified by quartile) obtained from the TCGA database (log-rank test).

(C) Immunoblotting analysis of OGT and c-Myc expressions in 30 pairs of human colorectal cancer tissues and matching peritumoral tissues. Data are presented as means ± SD. P-values were determined by unpaired two-tailed Student's t-tests..

(D) Quantitative PCR analysis was performed to detect the expression of the OGT in a panel of colon cancer cell lines (HT-29, HCT116, RKO, SW620, LoVo) and one normal colon cell lines (NCM460). n = 3; Data are presented as means ± SD. P-values were determined by unpaired two-tailed Student's t-tests.

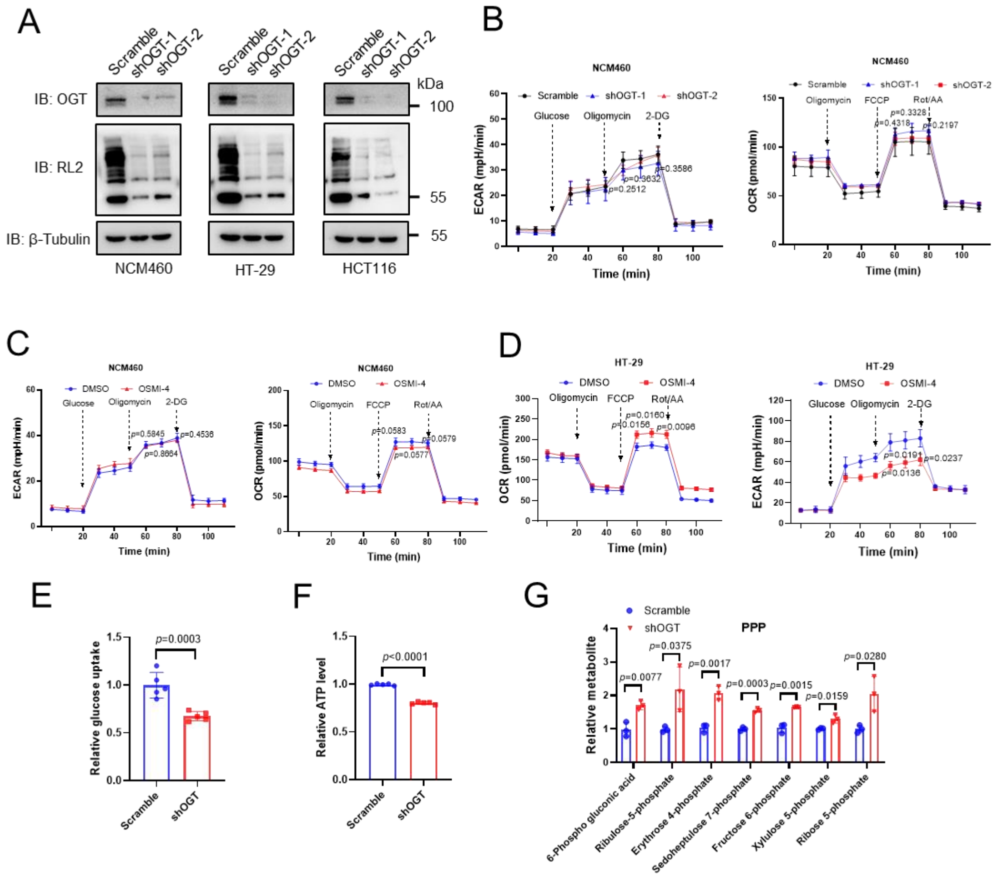

Figure S2

(A) Immunoblotting analysis of OGT and RL2 in NCM460, HT-29 and HCT116 cells expressing Scramble or shOGT.

(B) The extracellular acidification rate (ECAR) and oxygen consumption rate (OCR) in NCM460 cells expressing Scramble or shOGT. n = 3; Data are presented as means ± SD. P-values were determined by unpaired two-tailed Student's t-tests.

(C-D) The extracellular acidification rate (ECAR) and oxygen consumption rate (OCR) in NCM460 (C) or HT-29 cells (D) upon OSMI4 treatment. n = 3; Data are presented as means ± SD. P-values were determined by unpaired two-tailed Student's t-tests.

(E-F) Comparison of glucose uptake (E) and ATP (F) in HT-29 cells expressing Scramble or shOGT. n = 3; Data are presented as means ± SD. P-values were determined by unpaired two-tailed Student's t-tests.

(G) Relative abundance of metabolites derived from PPP HT-29 cells expressing Scramble or shOGT. n = 3; Data are presented as means ± SD. P-values were determined by unpaired two-tailed Student's t-tests.

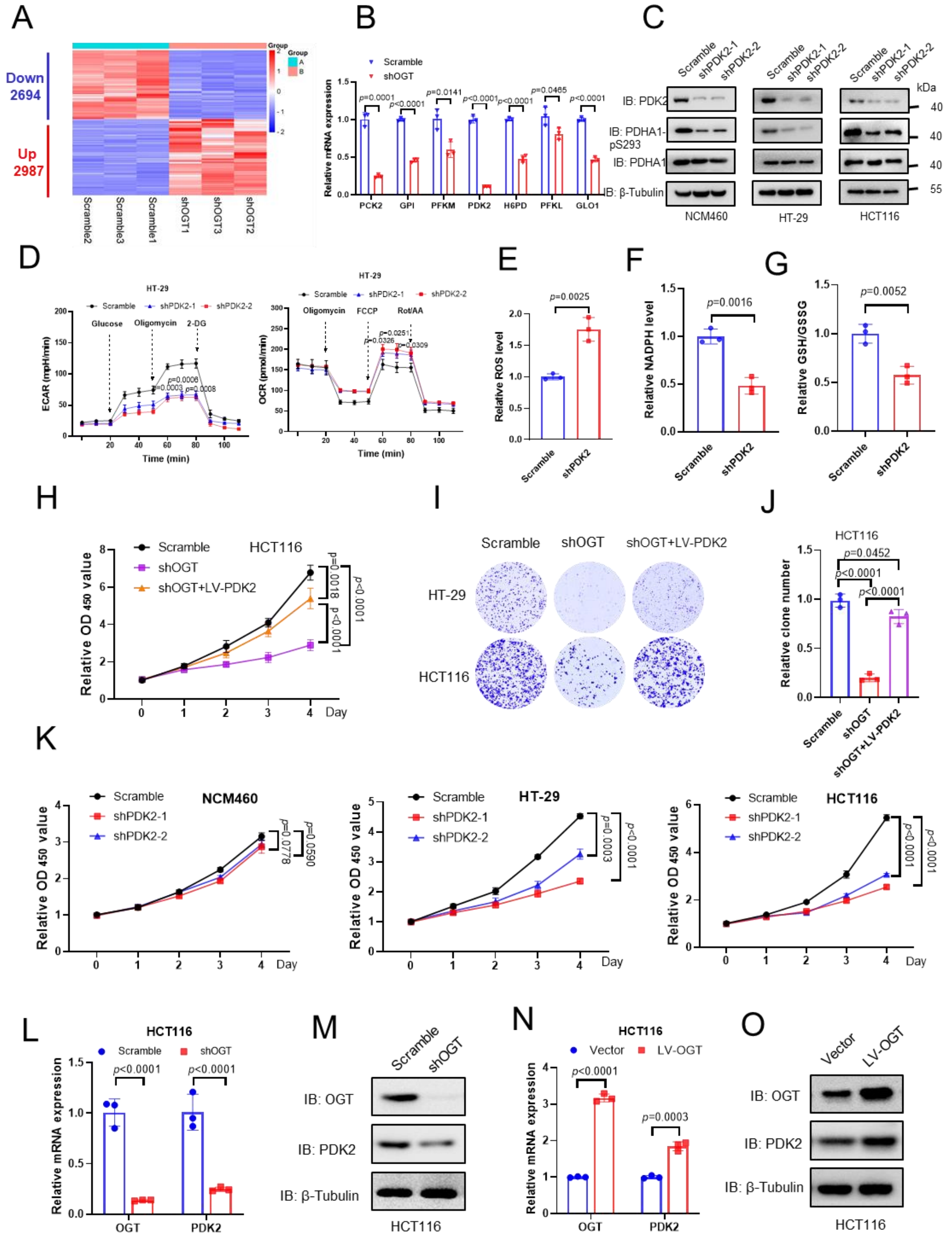

Figure S3

(A) The heatmap showing the genes that were differentially expressed in OGT-knockdown HT-29 cells and control cells.

(B) Quantitative PCR analysis was performed to detect the expression of the downregulated genes in glucose metabolism from the RNA-seq analysis in HT-29. n = 3; Data are presented as means ± SD. P-values were determined by unpaired two-tailed Student’s t-tests.

(C) Immunoblotting analysis of PDH phosphorylation in NCM460, HT-29 and HCT116 cells expressing Scramble or shPDK2.

(D) The extracellular acidification rate (ECAR) and oxygen consumption rate (OCR) in HT-29 cells infected with Scramble or shPDK2. n = 3; Data are presented as means ± SD. P-values were determined by unpaired two-tailed Student's t-tests.

(E-G) Analysis of ROS (E), NADPH (F), GSH/GSSG (G) in HT-29 cells expressing Scramble or shPDK2. n = 3; Data are presented as means ± SD. P-values were determined by unpaired two-tailed Student's t-tests.

(H) Cell proliferation of HCT116 cells expressing scramble, shOGT or shOGT with PDK2 overexpression. n = 5; Data are presented as means ± SD. P-values were determined by unpaired two-tailed Student's t-tests.

(I) Representative images of HT-29 and HCT116 cell clones staining with crystal violet.

(J) Statistical analyses of relative colon numbers in HCT116 cells expressing Scramble, shOGT or shOGT with PDK2 overexpression. n = 3; Data are presented as means ± SD. P-values were determined by unpaired two-tailed Student's t-tests.

(K) Cell proliferation of NCM460, HT-29, HCT116 cells upon PDK2 knockdown. n = 5; Data are presented as means ± SD. P-values were determined by unpaired two-tailed Student's t-tests.

(L-M) Quantitative PCR analysis (L) and immunoblotting analysis (M) of PDK2 expression in HCT116 cells expressing scramble or shOGT. n = 3; Data are presented as means ± SD. P-values were determined by unpaired two-tailed Student's t-tests.

(N-O) Quantitative PCR analysis (N) and immunoblotting analysis (O) of PDK2 expression in HCT116 cells expressing control vector or LV-OGT. n = 3; Data are presented as means ± SD. P-values were determined by unpaired two-tailed Student's t-tests.

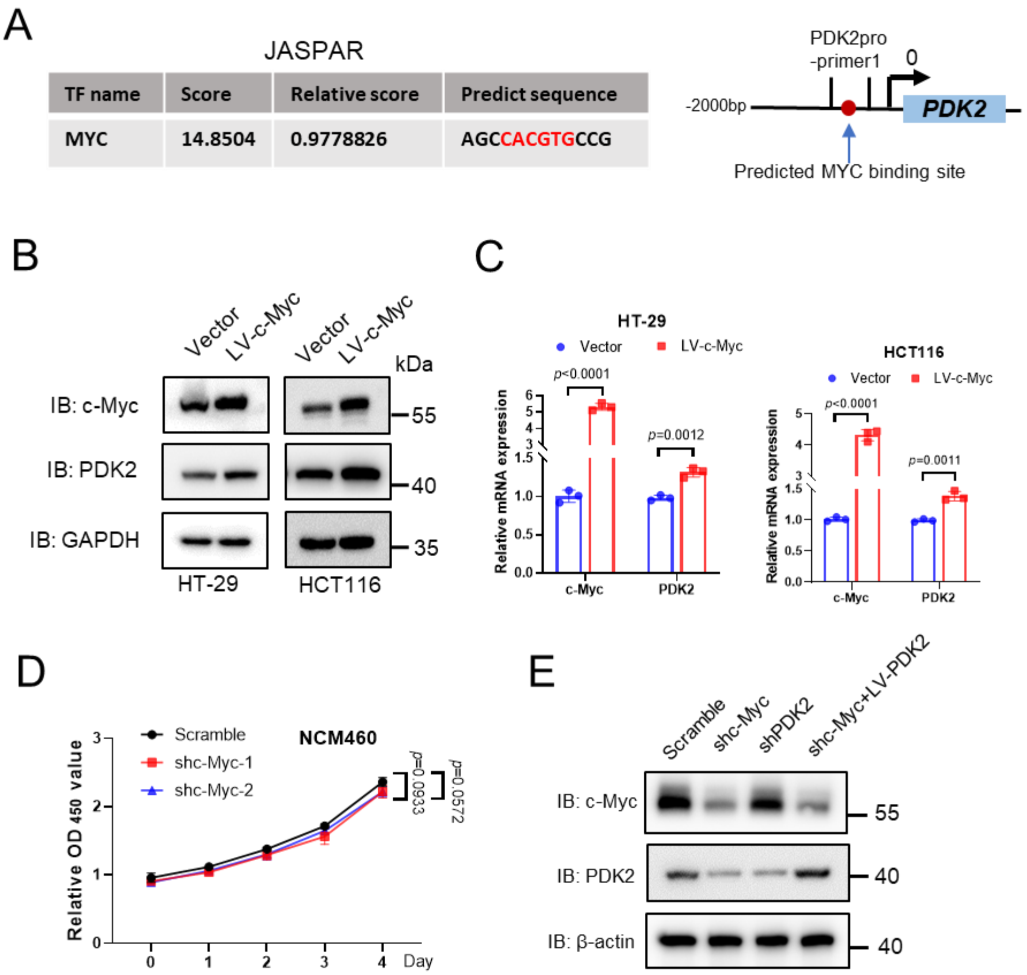

Figure S4

(A) Schematic illustration of the potential binding sites of c-Myc on the promoter of PDK2.

(B-C) Quantitative PCR analysis (B) and immunoblotting analysis (C) of PDK2 expression in HT-29 and HCT116 cells expressing control vector or LV-c-Myc. n = 3; Data are presented as means ± SD. P-values were determined by unpaired two-tailed Student's t-tests.

(D) Cell proliferation of NCM460 cells upon c-Myc knockdown with small hairpin RNAs (shRNA) (n = 5 independent assays). c-Myc was depleted in the indicated cells with two independent shRNAs (1 and 2). n = 5; Data are presented as means ± SD. P-values were determined by unpaired two-tailed Student's t-tests.

(E) Immunoblotting analysis of PDK2 and c-Myc expression in HT-29 infected with Scramble, shc-Myc, shPDK2 or shc-Myc with PDK2 overexpression.

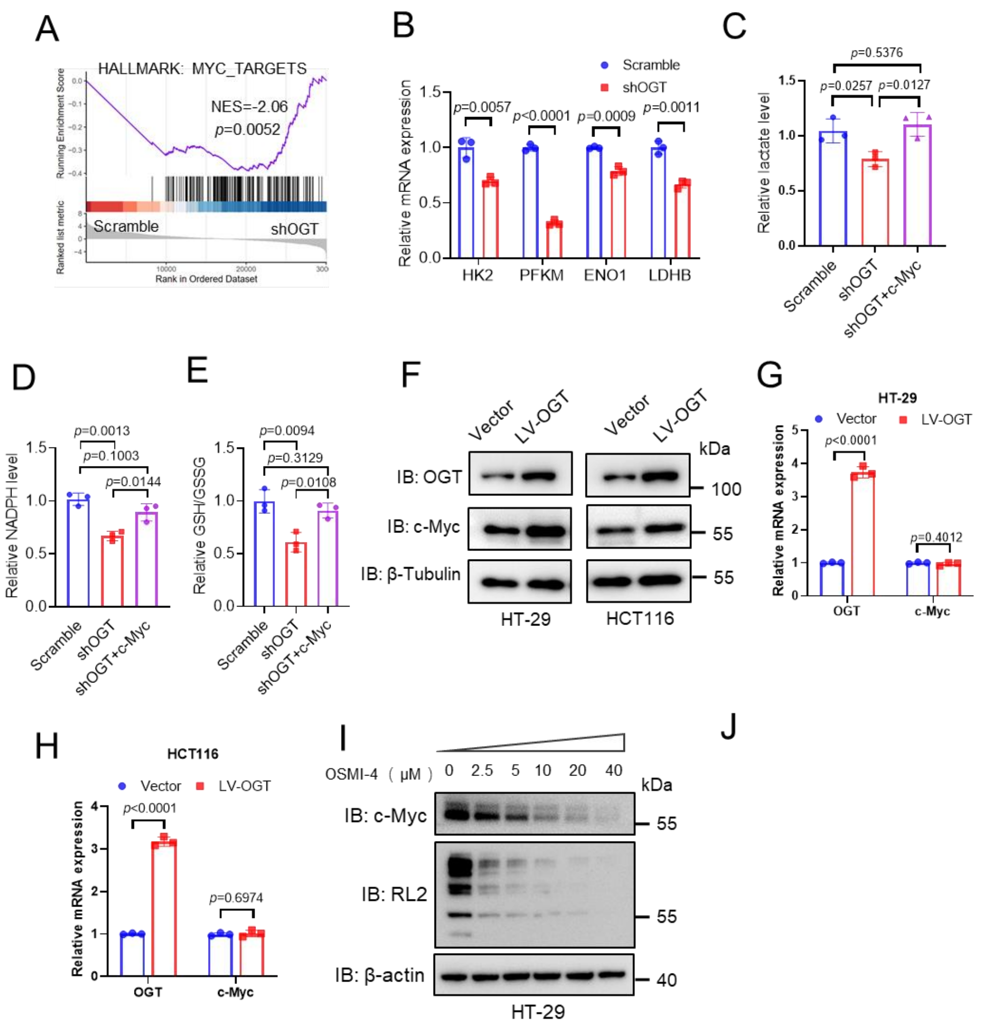

Figure S5

(A) GSEA analysis of OGT-regulated gene signature versus c-Myc-targeted pathway.

(B) Quantitative PCR analysis of HK2, PFKM, ENO1 and LDHB in HT-29 cells upon OGT knockdown. n = 3; Data are presented as means ± SD. P-values were determined by unpaired two-tailed Student's t-tests.

(C-E) Comparison of lactate (C), NADPH (D), GSH/GSSG (E) in HT-29 cells infected with scramble, shOGT or shOGT reconstituted with c-Myc expression. n = 3; Data are presented as means ± SD. P-values were determined by unpaired two-tailed Student's t-tests.

(F-H) Immunoblotting analysis (F) and quantitative PCR analysis (G and H) of c-Myc and OGT expression in HT-29 and HCT116 cells expressing control vector or LV-OGT.

n = 3; Data are presented as means ± SD. P-values were determined by unpaired two-tailed Student's t-tests.

(I) Immunoblotting analysis of c-Myc expression upon treatment with different concentrations of OSMI-4.

(J) Quantitative PCR analysis of c-Myc mRNA expression upon treatment with DMSO, OSMI-4 and TMG. n = 3; Data are presented as means ± SD. P-values were determined by unpaired two-tailed Student's t-tests.

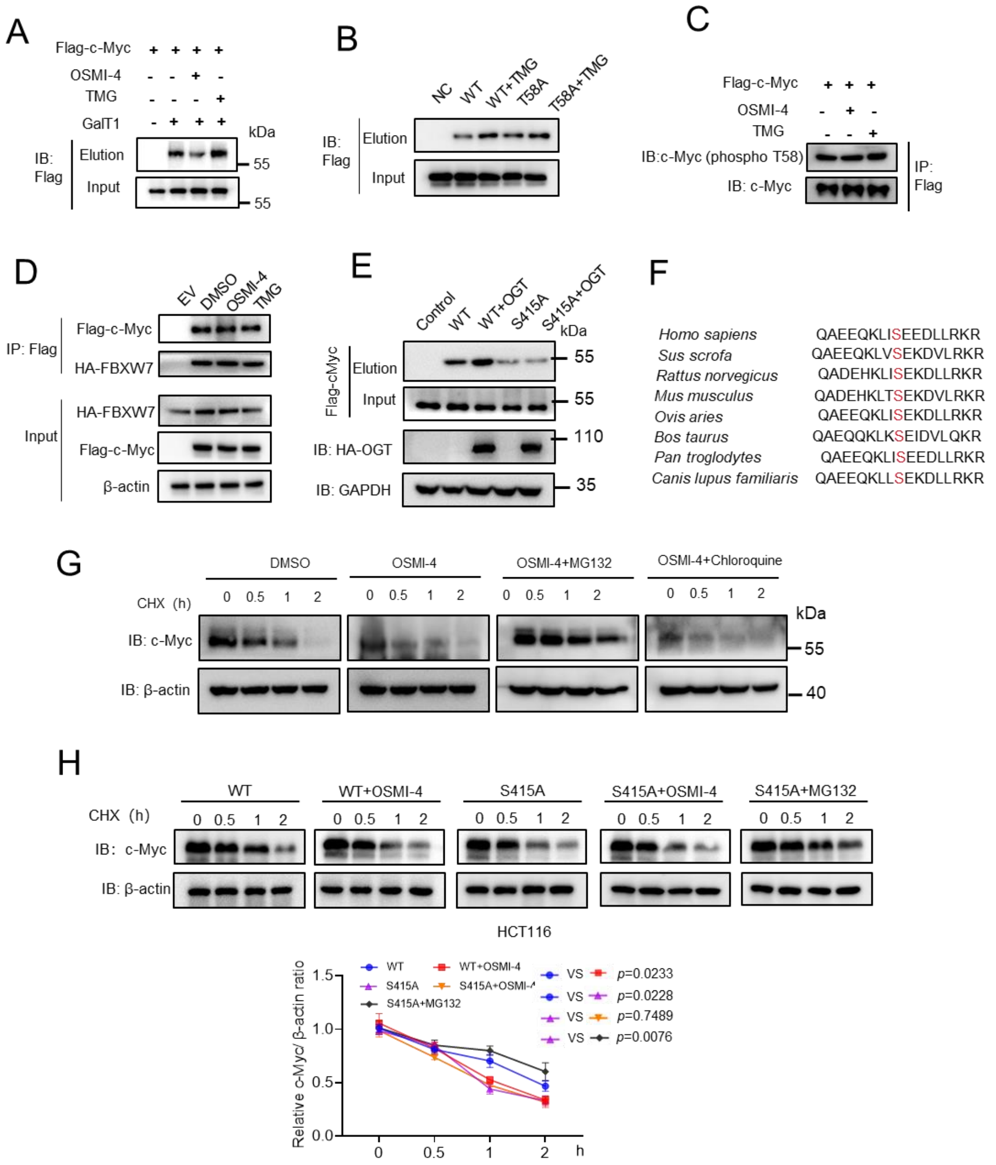

Figure S6

(A) Immunoblotting analysis of c-Myc O-GlcNAcylation upon treatment with DMSO, OSMI-4 and TMG.

(B) Immunoblotting analysis of WT and T58A c-Myc O-GlcNAcylation in HT29 cells in the presence or absence of TMG treatment.

(C) Immunoblotting analysis of T58 c-Myc phosphorylation in HT29 cells upon treatment with DMSO, OSMI-4 and TMG.

(D) Analysis of c-Myc-FBXW7 interaction in HT29 cells overexpressing WT Flag-tagged c-Myc and HA-tagged FBXW7 upon treatment with DMSO, OSMI-4 and TMG. Immunoprecipitation was performed using Flag-tagged antibody. Immunoblotting analyses were performed with the indicated antibodies.

(E) Immunoblotting analysis of WT and T415A c-Myc O-GlcNAcylation in HT29 cells with or without OGT expression.

(F) Comparison of amino acid sequences containing the glycosylation site among different species.

(G) Immunoblotting analysis of c-Myc levels in HT-29 cells by CHX treatment in the presence of inhibitors for proteasome (MG132) or lysosome (chloroquine).

(H) Immunoblotting analysis of c-Myc levels in HCT116 cells with c-Myc knockdown and reconstituted expression of shRNA-resistant WT, S415A c-Myc by CHX treatment in the presence of inhibitors for OGT (OSMI4) and proteasome (MG132).

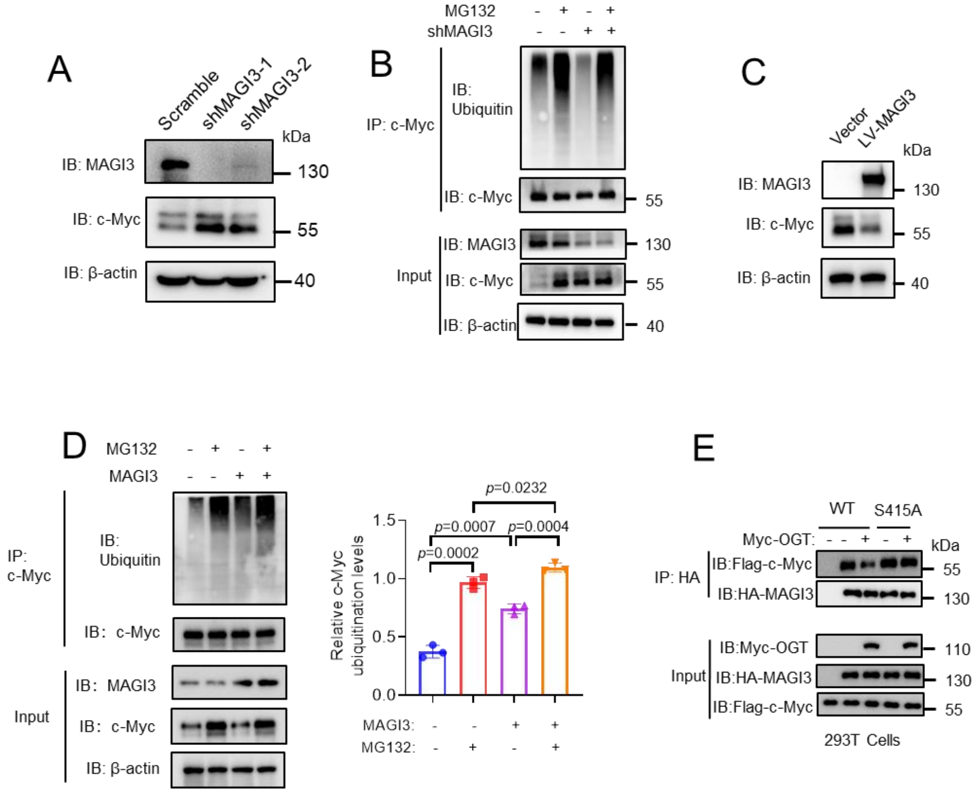

Figure S7

(A-D) Immunoblotting analysis of c-Myc protein expression and c-Myc ubiquitination in HT-29 cells knocked down MAGI3 (A and B) and over-expressed MAGI3 (C and D). Data are presented as means ± SD. P-values were determined by unpaired two-tailed Student's t-tests.

(E) Analysis of c-Myc-MAGI3 interaction in 293T cells overexpressing WT or S415A Flag-tagged c-Myc and HA-tagged MAGI3 with or without OGT overexpression. Immunoprecipitation was performed using HA-tagged antibody. Immunoblotting analyses were performed with the indicated antibodies.

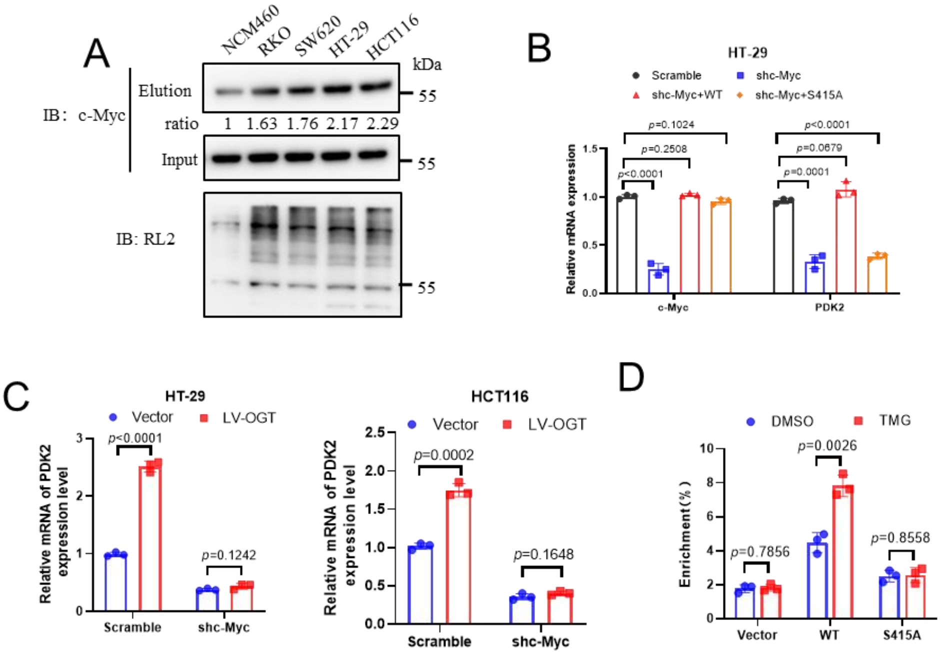

Figure S8

(A) Immunoblotting analysis of c-Myc glycosylation in NCM460 and CRC cells (RKO, SW620, HT-29 and HCT116).

(B) Quantitative PCR analysis of c-Myc and PDK2 expression in HT-29 cells with c-Myc knockdown and reconstituted expression of shRNA-resistant WT, S415A c-Myc in the presence or absence of OGT overexpression.

(C) Quantitative PCR analysis of PDK2 expression in HT-29 cells expressing Scramble or shc-Myc with or without OGT overexpression.

(D) ChIP-qPCR analysis of c-Myc binding to PDK2 promoter in HT-29 cells with c-Myc knockdown and reconstituted expression of shRNA-resistant WT, S415A c-Myc upon TMG treatment. Ectopic c-Myc was pulled down by the anti-c-Myc antibody. n = 3; Data are presented as means ± SD. P-values were determined by unpaired two-tailed Student's t-tests.

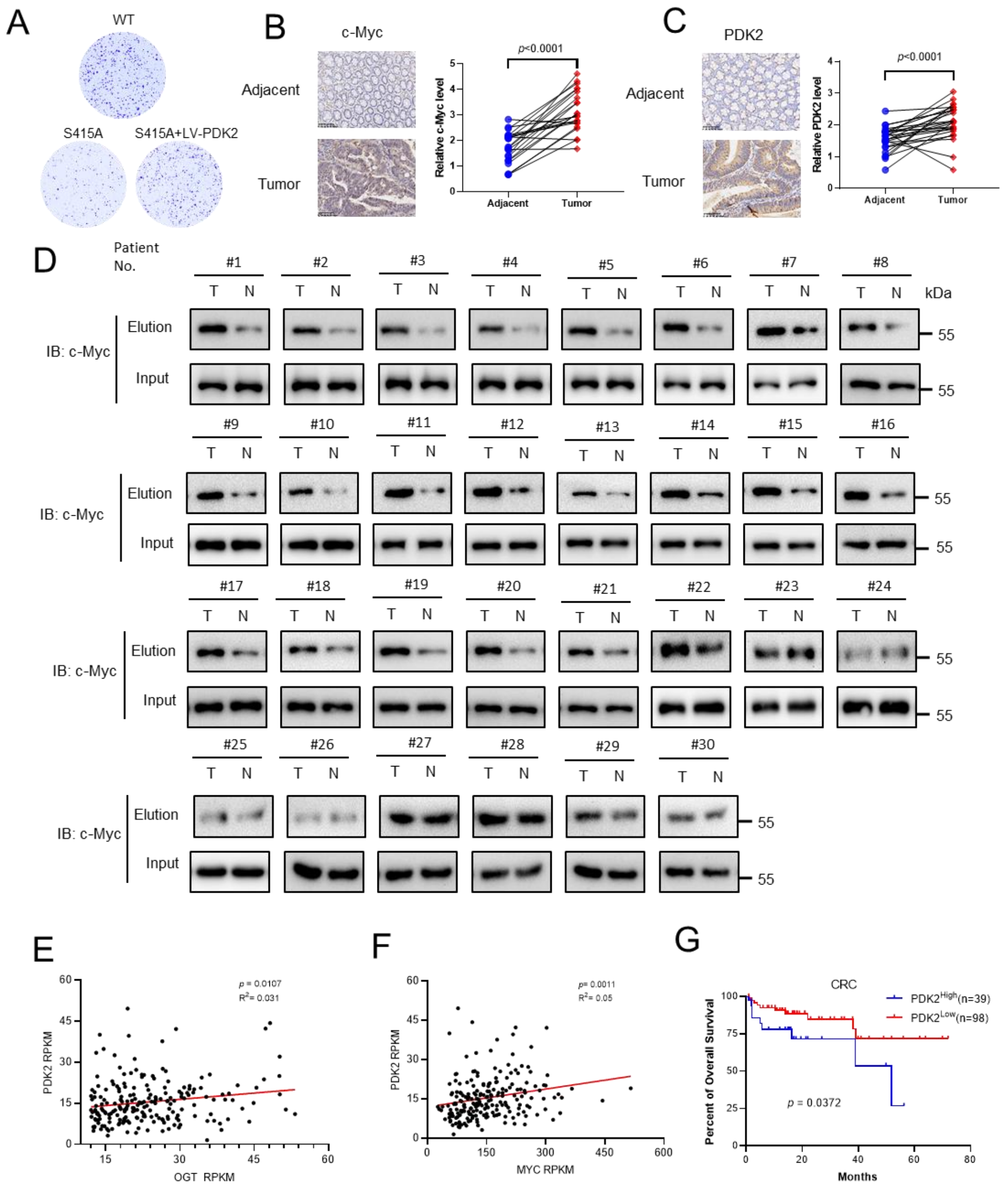

Figure S9

(A) Representative images of HT-29 cell clones staining with crystal violet.

(B-C) Immunohistochemical analysis of PDK2(B) and c-Myc (C) expression in CRC tissues and the matched peritumoral tissues (n = 30 pairs). Quantification of PDK2 and c-Myc levels were shown.

(D) Immunoblotting analysis of c-Myc glycosylation in CRC tissues and the matched peritumoral tissues (n = 30 pairs).

(E-F) Analysis of the correlation of mRNA expression of OGT- PDK2 (E) and c-Myc -PDK2 (F) in CRC patients from The Cancer Genome Atlas (TCGA) database.

(G) Kaplan–Meier survival curves were analyzed and compared between patients with low (n = 98) and high (n = 39) levels of PDK2 in CRC patients from The Cancer Genome Atlas (TCGA) database. Subgroups with high- or low-PDK2 expression were sorted according to the TPM gene expression standard values.
