# Supplementary material for: The OGT–c-Myc–PDK2 axis rewires the TCA cycle and promotes colorectal tumor growth
Source: Cell Death Differ. 2024 May 22;31(9):1157–69. doi: 10.1038/s41418-024-01315-4 (PMC11369260; doi:10.1038/s41418-024-01315-4)
Supplement: Supplementary file 2 — Supplementary Table [file 41418_2024_1315_MOESM2_ESM.pdf]

Supplementary Table 1: Information about antibodies used in this study.			
**Antibody**	**Brand**	**Cat#**	**Dilution**
anti-OGT	Abcam	ab177941	1:1000
anti-RL2	Abcam	ab2739	1:1000
c-Myc	Cell Signaling Technology	# 9402	1:1000
PDK2	Abcam	ab68164	1:1000
MAGI3	Santa Cruz	sc-136471	1:200
PDHA1	Abcam	ab168379	1:1000
PDHA1(phospho-Ser293)	Abcam	ab177461	1:1000
anti-HA	Cell Signaling Technology	#3724	1:1000
Anti-Ubiquitin	Abcam	ab134953	1:1000
anti-Flag	Abcam	ab125243	1:1000
anti-β-actin	Proteintech	HRP-60008	1:1000
anti-β-Tubulin	Abcam	ab6160	1:1000
GAPDH	Proteintech	HRP-60004	1:1000
Streptavidin	Proteintech	SA00001-0	1:1000
c-Myc(phospho-T58)	Abcam	ab185655	1:1000

Supplementary Table 2: Sequences of primers used in this study.	
Primers	Sequences(5' to 3')
β-actin-F	GAGAGCGCCCAGGCTATTT
β-actin-R	CTCCTTAATGTCACGCACGAT
OGT-F	TCCTGATTTGTACTGTGTTCGC
OGT-R	AAGCTACTGCAAAGTTCGGTT
PDK2-F	ATGAAAGAGATCAACCTGCTTCC
PDK2-R	GGCTCTGGACATACCAGCTC
c-Myc-F	GGCTCCTGGCAAAAGGTCA
c-Myc-R	CTGCGTAGTTGTGCTGATGT
PCK2-F	GCCATCATGCCGTAGCATC
PCK2-R	AGCCTCAGTTCCATCACAGAT
GPI-F	CCGCGTCTGGTATGTCTCC
GPI-R	CCTGGGTAGTAAAGGTCTTGGA
PFKM-F	AGCGTTTCGATGATGCTTCAG
PFKM-R	GGAGTCGTCCTTCTCGTTCC
H6PD-F	GCAGAGCACAAGGATCAGTTC
H6PD-R	GGCAGCTACTGTTGATGTTGC
PFKL-F	GGCTTCGACACCCGTGTAA
PFKL-R	CGTCAAACCTCTTGTCATCCA
GLO1-F	AGCAGACCATGCTACGAGTGA
GLO1-R	GAGAGCGCCCAGGCTATTT
shOGT-1F	CCGGTTTAGCACTCTGGCAATTAAACTCGAGTTTAATTGCCAGAGTGCTAAATTTTTG
shOGT-1R	AATTCAAAAATTTAGCACTCTGGCAATTAAACTCGAGTTTAATTGCCAGAGTGCTAAA
shOGT-2F	CCGGGCCCTAAGTTTGAGTCCAAATCTCGAGATTTGGACTCAAACTTAGGGCTTTTTG
shOGT-2R	AATTCAAAAAGCCCTAAGTTTGAGTCCAAATCTCGAGATTTGGACTCAAACTTAGGGC
shPDK2-1F	CCGGGCTCCTGTGTGACAAGTATTACTCGAGTAATACTTGTCACACAGGAGCTTTTTG
shPDK2-1R	AATTCAAAAAGCTCCTGTGTGACAAGTATTACTCGAGTAATACTTGTCACACAGGAGC
shPDK2-2F	CCGGACCTTGTTAGACCGAGAGCTTCTCGAGAAGCTCTCGGTCTAACAAGGTTTTTTG
shPDK2-2R	AATTCAAAAAACCTTGTTAGACCGAGAGCTTCTCGAGAAGCTCTCGGTCTAACAAGGT
shc-Myc-1F	CCGGCCATAATGTAAACTGCCTCAACTCGAGTTGAGGCAGTTTACATTATGGTTTTTG
shc-Myc-1R	AATTCAAAAACCATAATGTAAACTGCCTCAACTCGAGTTGAGGCAGTTTACATTATGG
shc-Myc-2F	CCGGACTGAAAGATTTAGCCATAATCTCGAGATTATGGCTAAATCTTTCAGTTTTTTG
shc-Myc-2R	AATTCAAAAAACTGAAAGATTTAGCCATAATCTCGAGATTATGGCTAAATCTTTCAGT
Scramble-F	CCGGGAATGTTTACTACACTCGGATCTCGAGATCCGAGTGTAGTA AACATTCTTTTTG
Scramble-R	AATTCAAAAAGAATGTTTACTACACTCGGATCTCGAGATCCGAGT GTAGTAAACATTC
c-Myc-T58A-F	TGCTGCCCGCCCCGCCCCTGTCCCCTA
c-Myc-T58A-R	GGGCGGGGCGGGCAGCAGCTCGAATTTCTTCCAG
c-Myc-S136/139A-F	ACTGTATGTGGGCCGGCTTCGCGGCCGCCGCCAAG
c-Myc-S136/139A-R	GGCGGCCGCGAAGCCGGCCCACATACAGTCCTGGAT

c-Myc-S159A-F	CGCAAAGACGCCGGCAGCCCGAACCCCG
c-Myc-S159A -R	GGGCTGCCGGCGTCTTTGCGCGCAGCCTG
c-Myc-S314/315A -F	CGCCTCCCGCCGCTCGGAAGGACTATCCTGCTGC
c-Myc-S314/315A -R	TCCTTCCGAGCGGCGGGAGGCGCTGCGTAGTTG
c-Myc-S415A-F	CAAAAGCTCATTGCTGAAGAGGACTTGTTGCGGAAACG
c-Myc-S415A-R	GTCCTCTTCAGCAATGAGCTTTTGCTCCTCTGCTTGGAC
PDK2pro-P1-F	CGGCAAAGCGTGGGTTAA
PDK2pro-P1-R	GCCAATCGGCTCGTCATG
