# Supplementary material for: The OGT–c-Myc–PDK2 axis rewires the TCA cycle and promotes colorectal tumor growth
Source: Cell Death Differ. 2024 May 22;31(9):1157–69. doi: 10.1038/s41418-024-01315-4 (PMC11369260; doi:10.1038/s41418-024-01315-4)
Supplement: Supplementary file 3 — Supplement Material-uncropped western blots [file 41418_2024_1315_MOESM3_ESM.pdf]

Figure1C

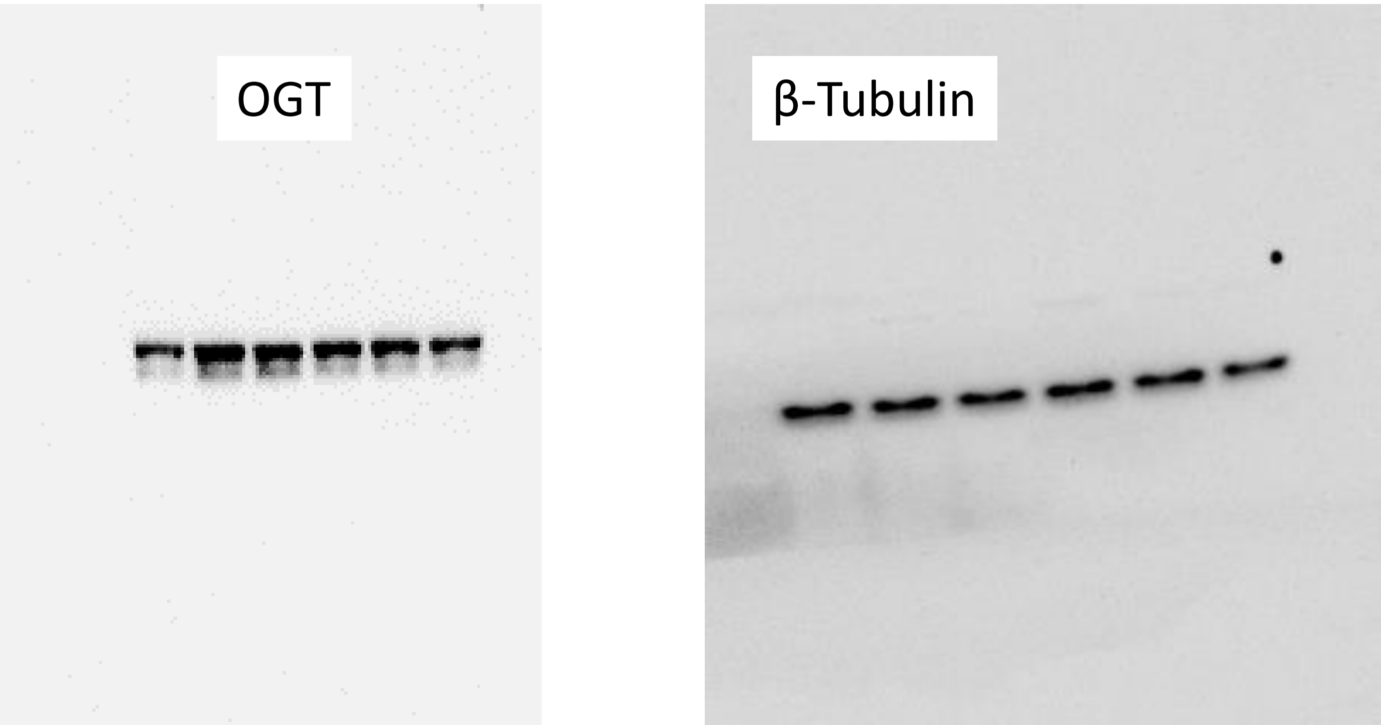

Figure2K

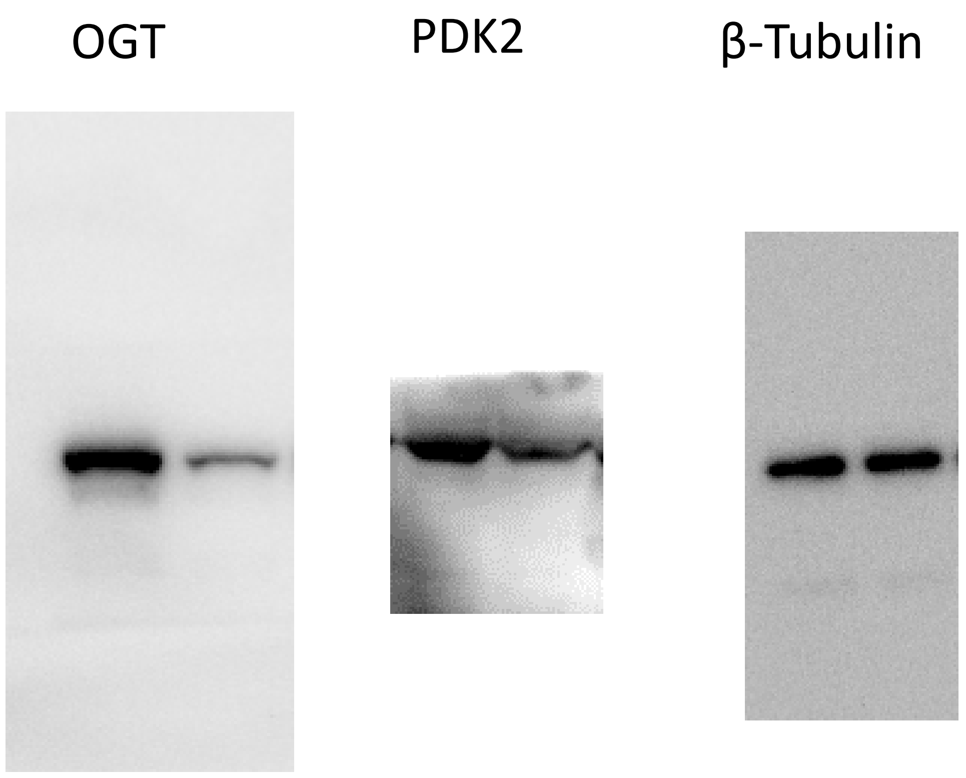

Figure2M

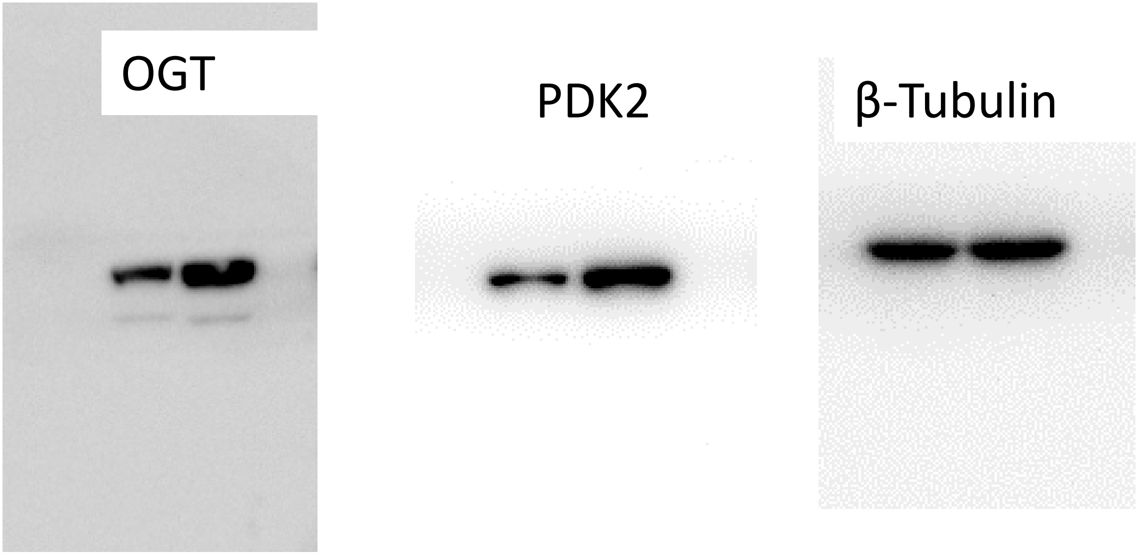

Figure3B

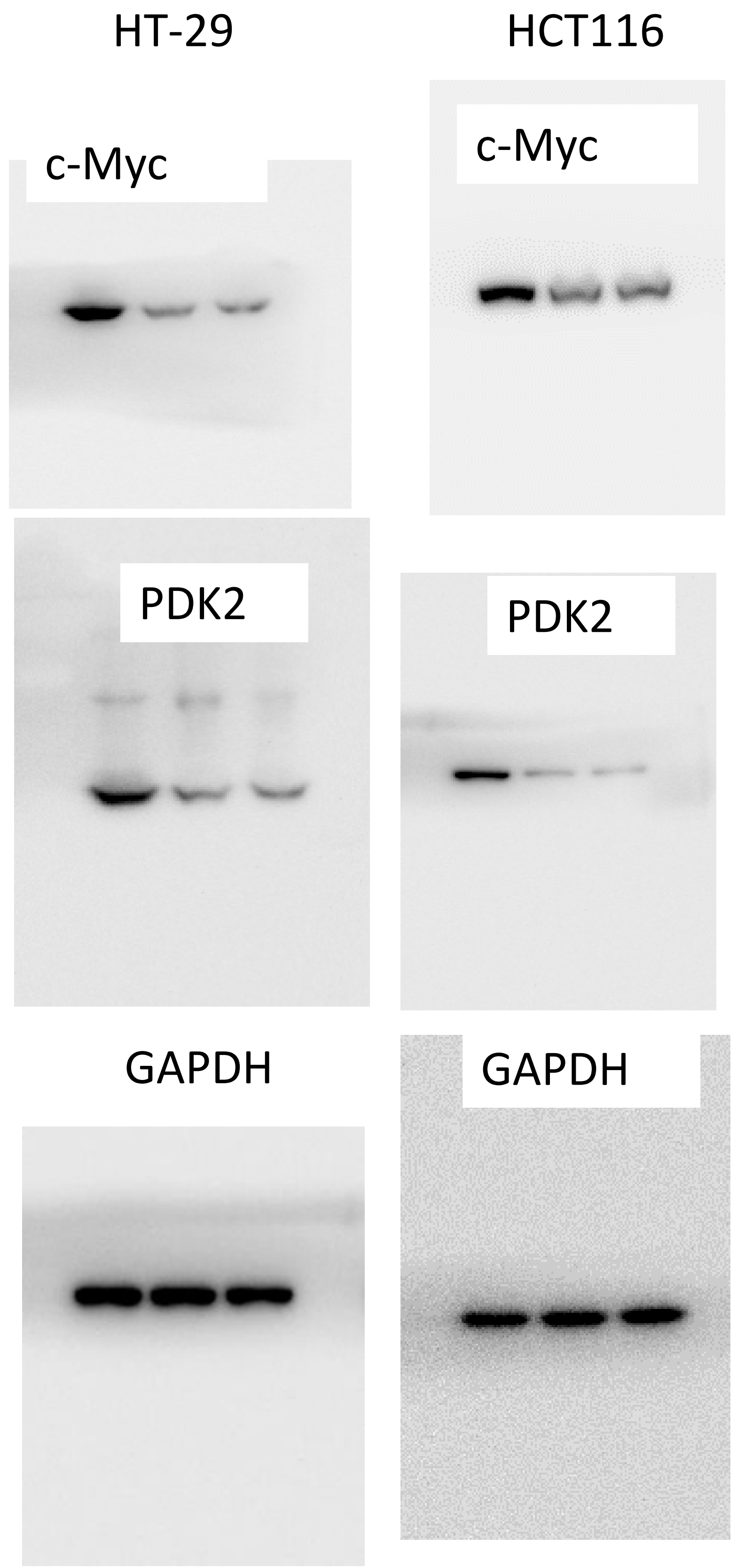

Figure4A

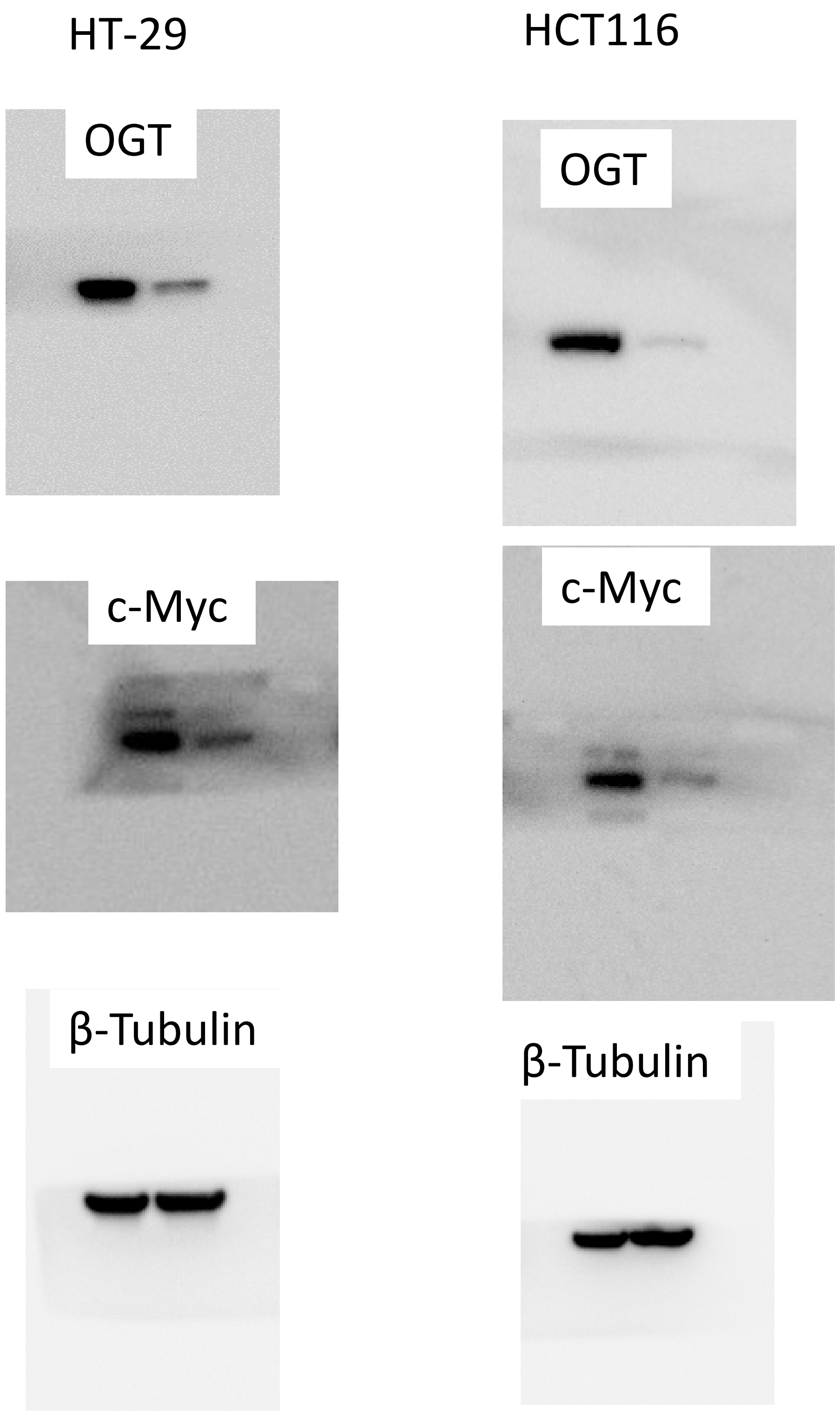

Figure4C

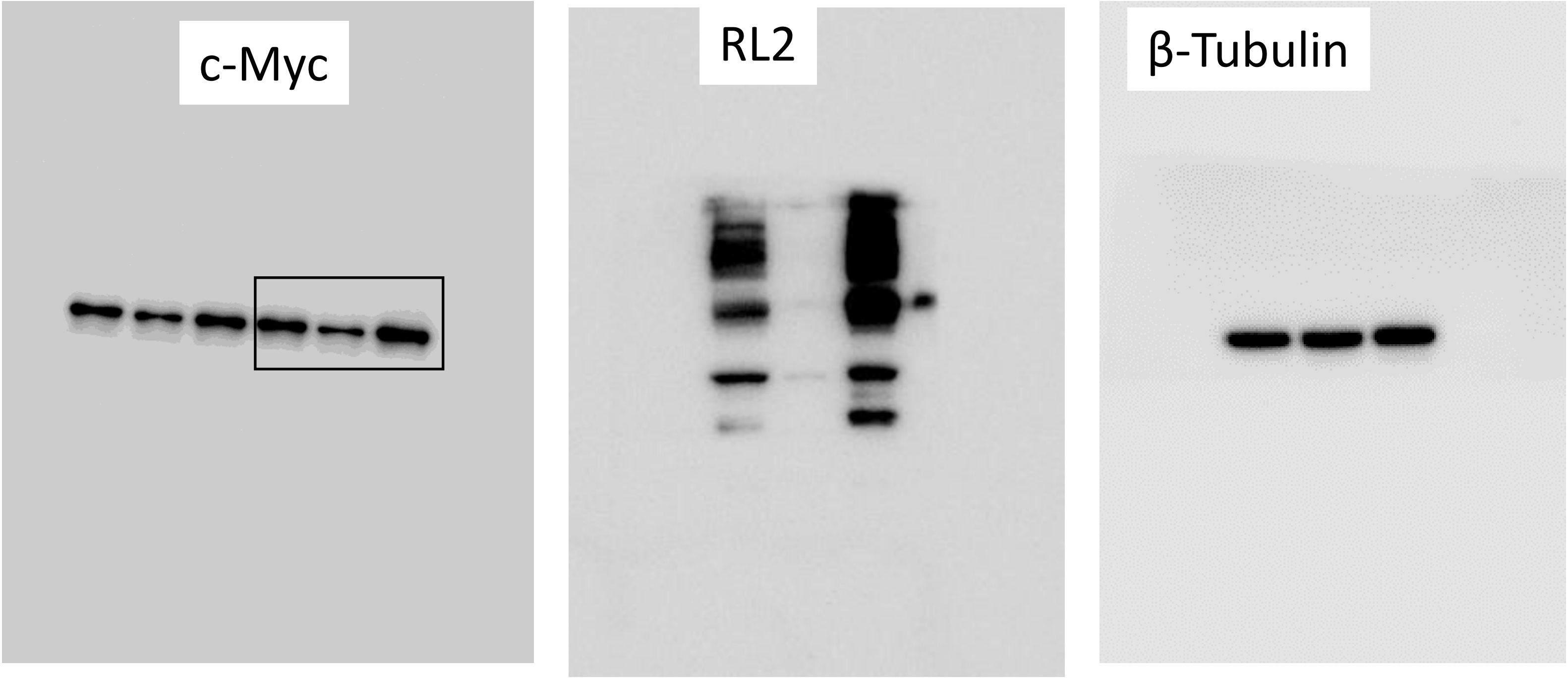

Figure4E

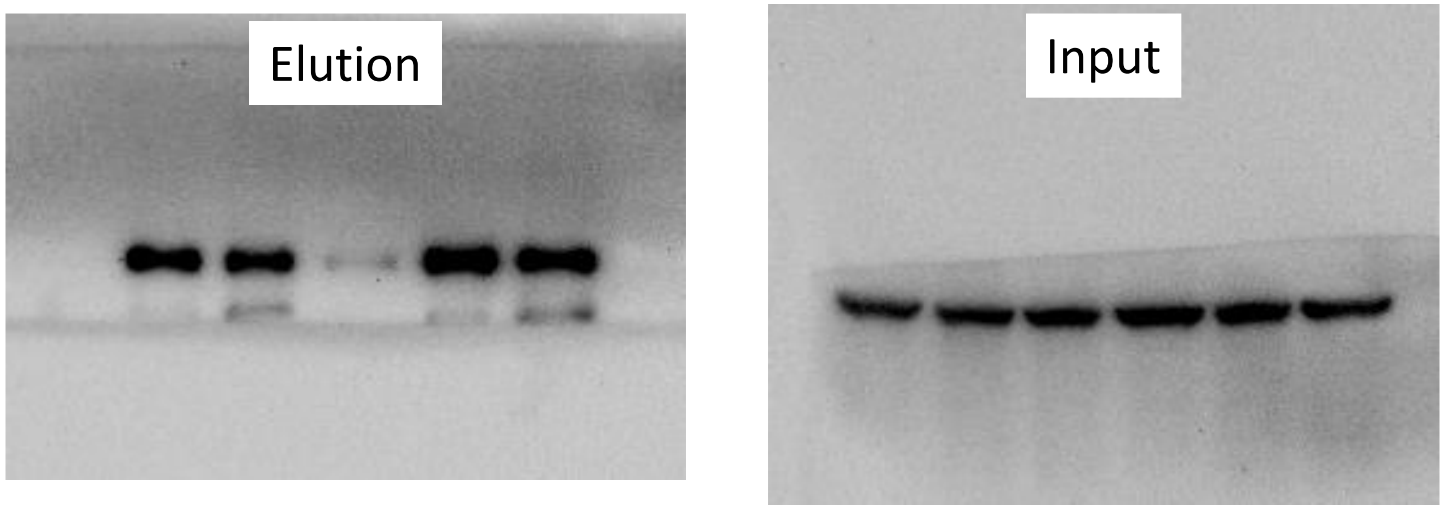

Figure4F

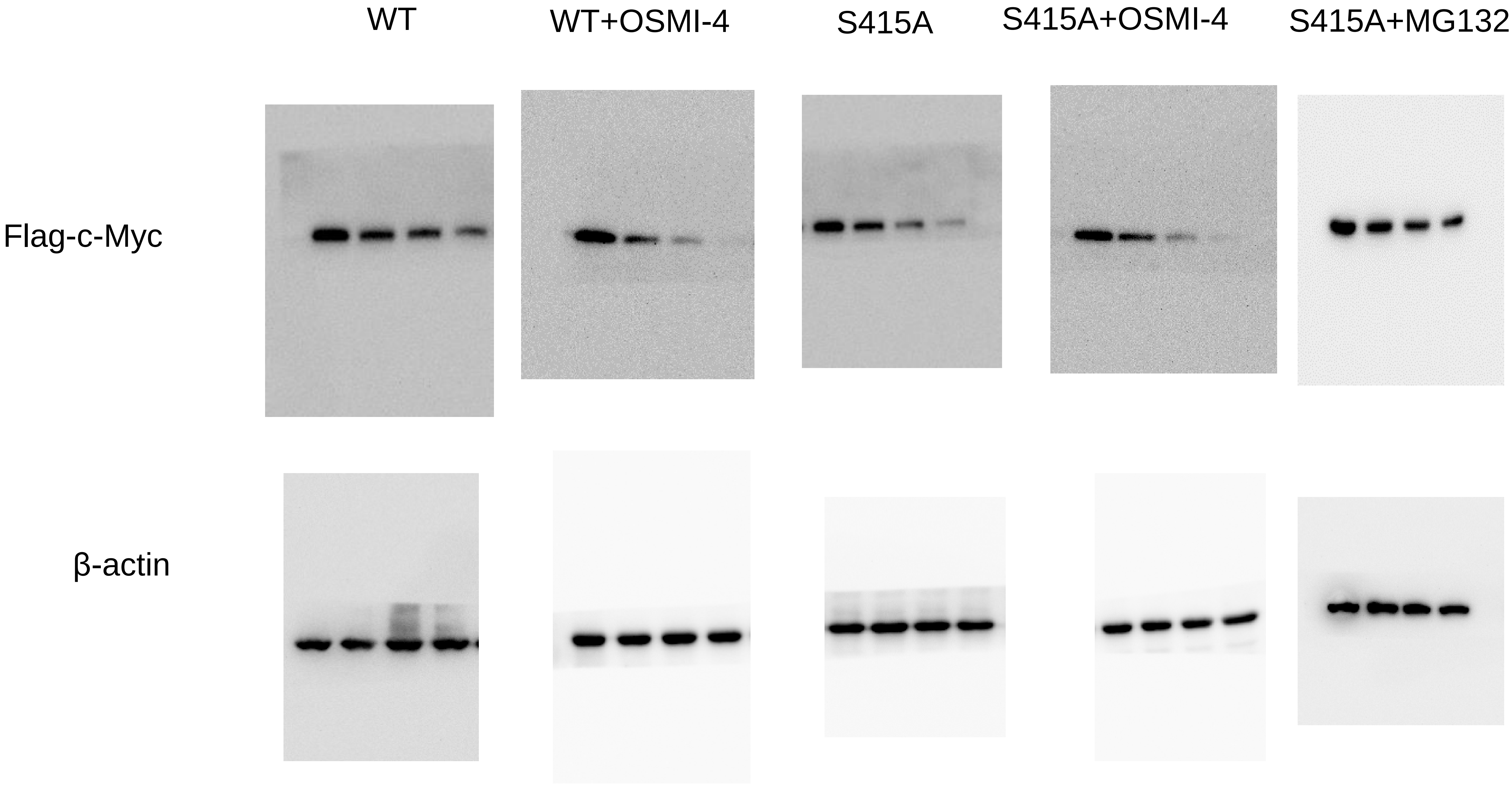

Figure4G

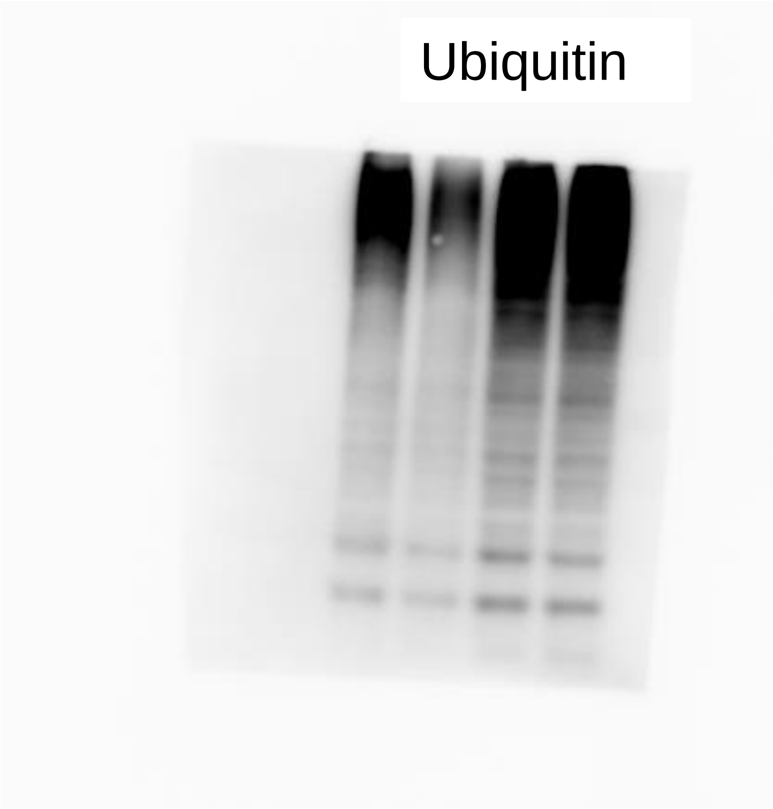

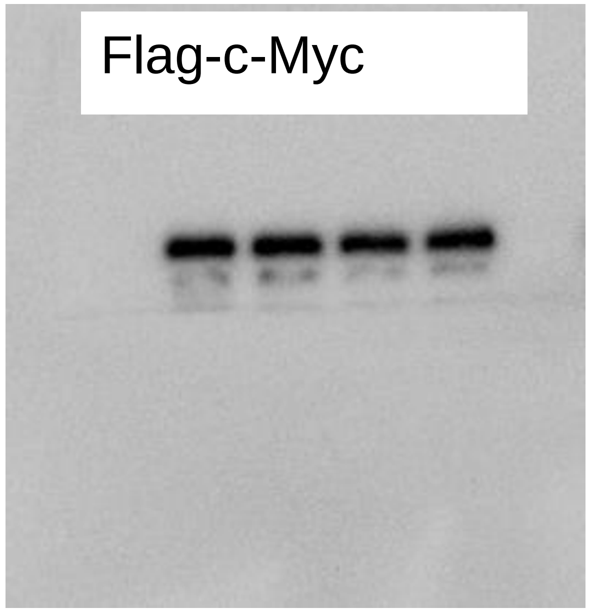

Figure4H

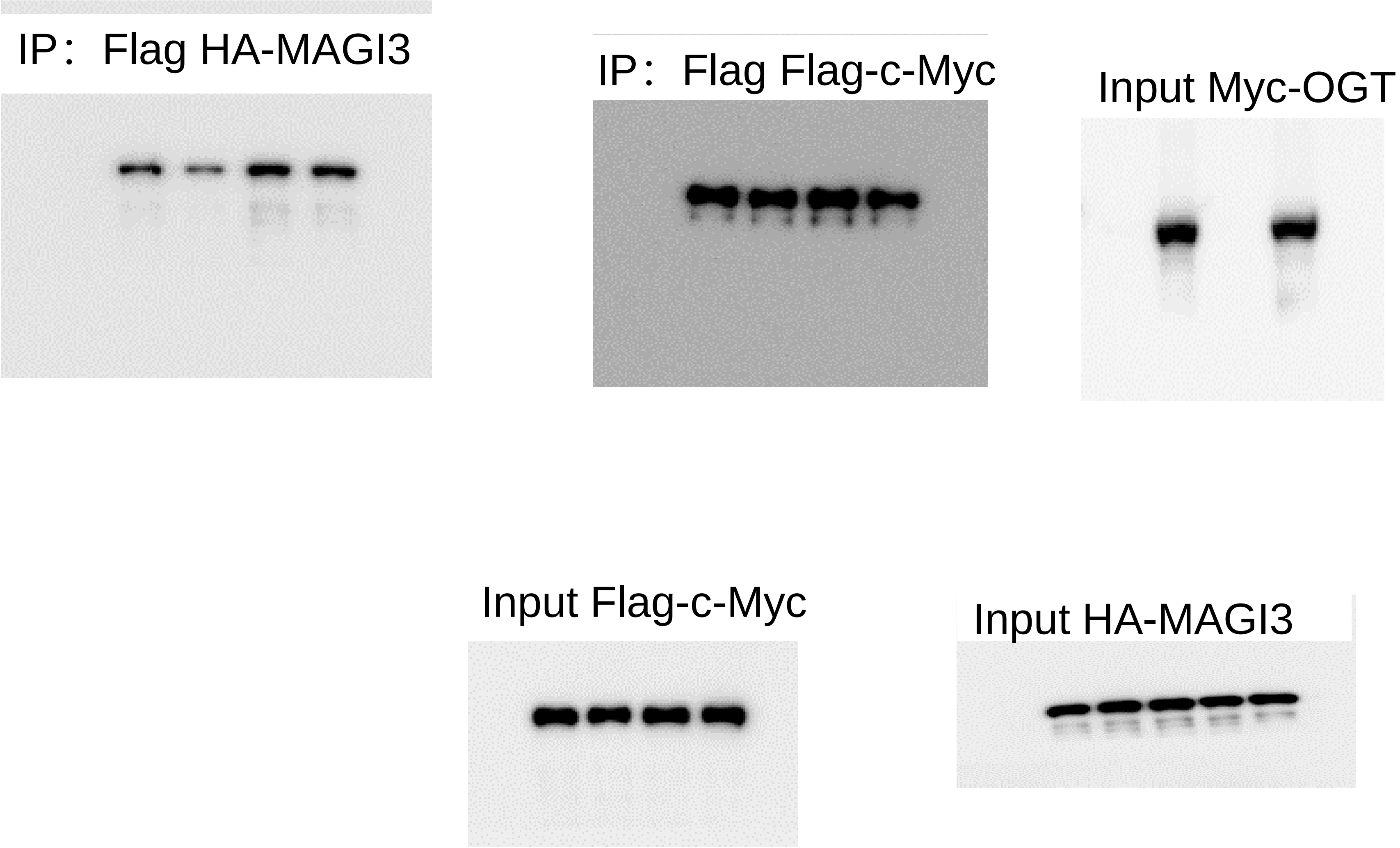

IP： Flag HA-MAGI3
IP： Flag Flag-c-Myc
Input Myc-OGT
Input Flag-c-Myc
Input HA-MAGI3

Figure4I

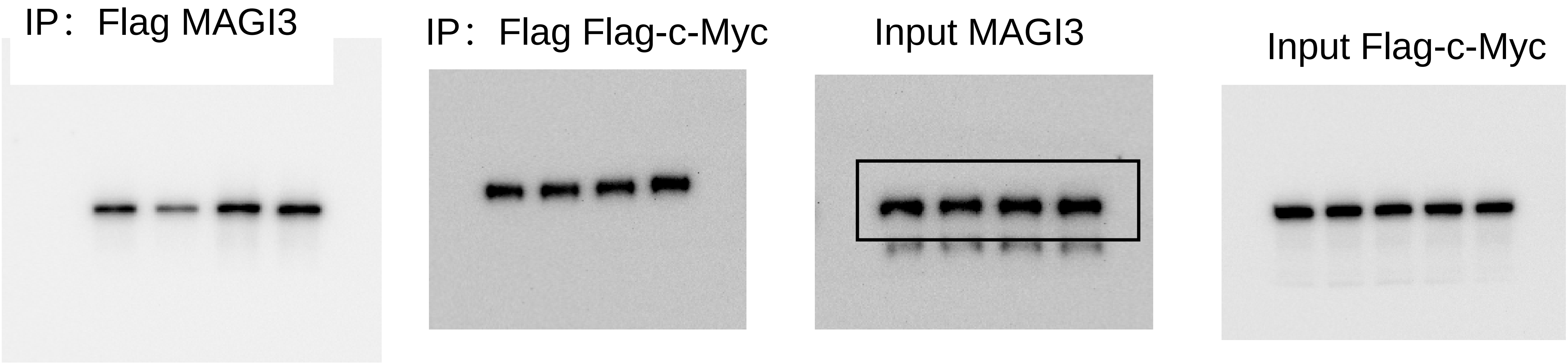

Figure5A

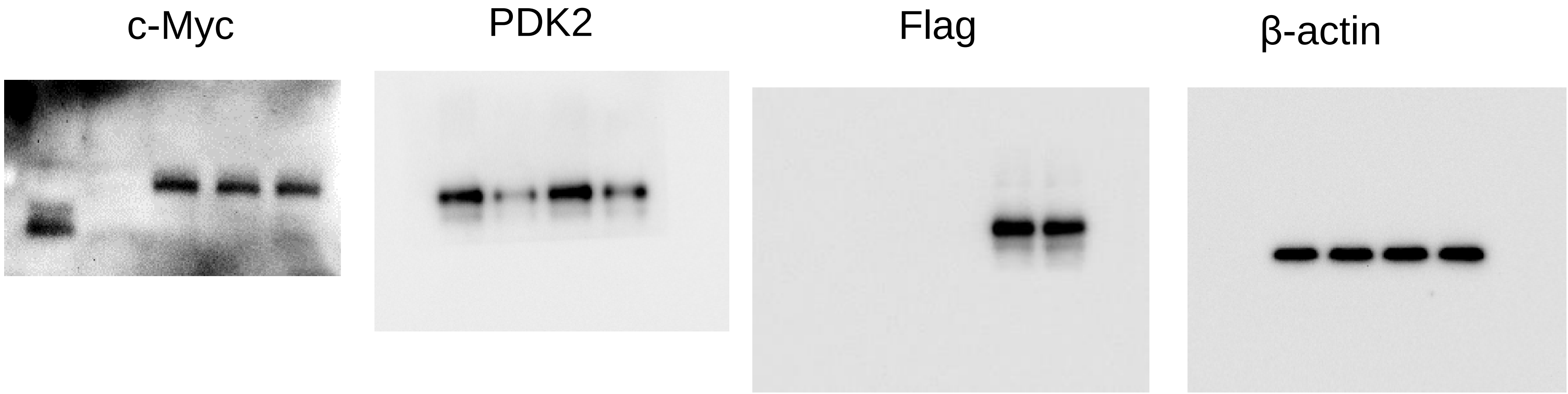

Figure5D

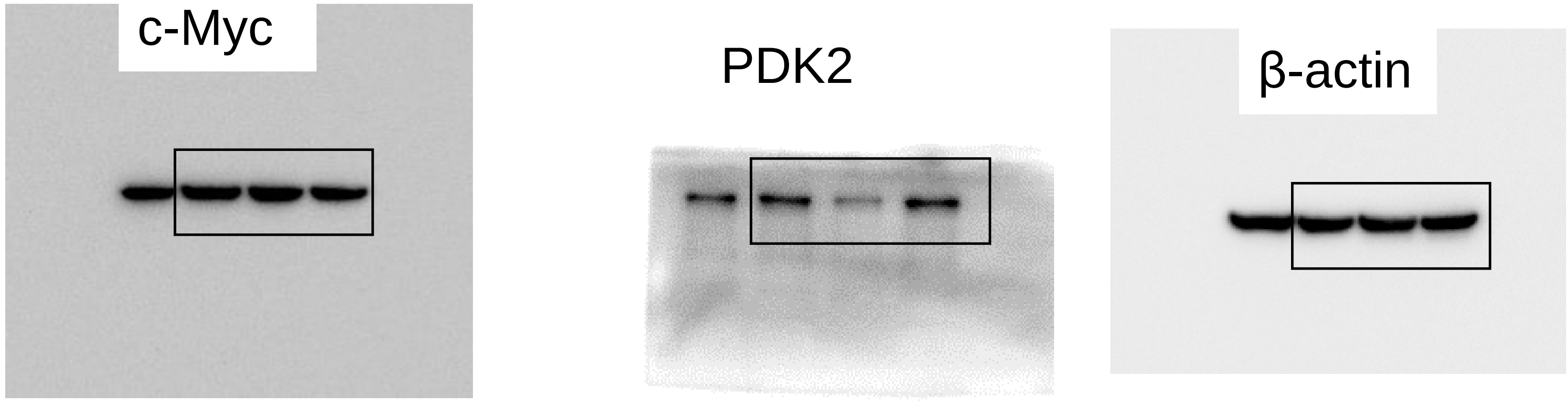

FigureS1C

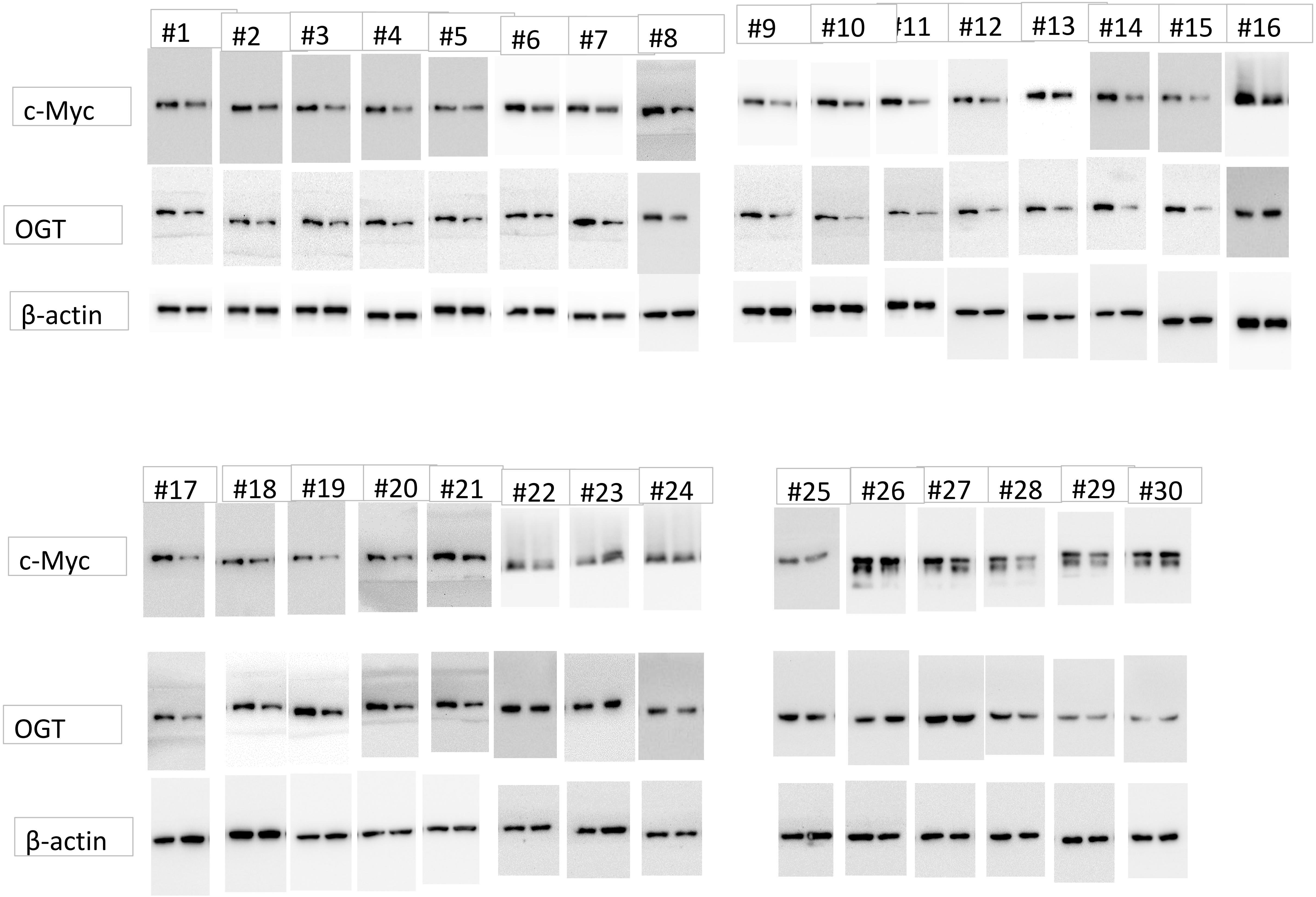
#1
#2
#3
#4
#5
#6
#7
#8
#9
#10
#11
#12
#13
#14
#15
#16
c-Myc
OGT
β-actin
#17
#18
#19
#20
#21
#22
#23
#24
#25
#26
#27
#28
#29
#30
c-Myc
OGT
β-actin

FigureS2A

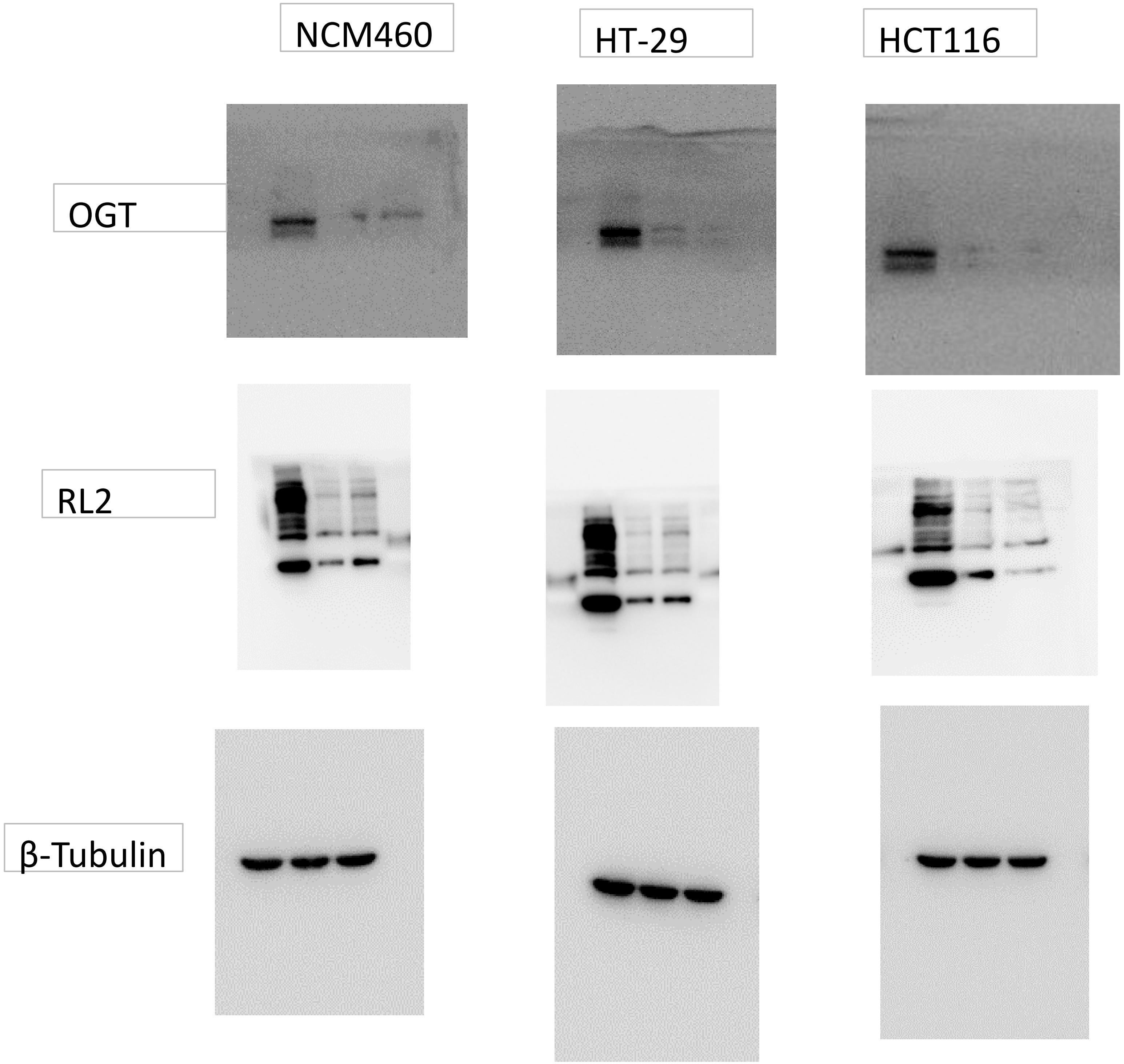

FigureS3C

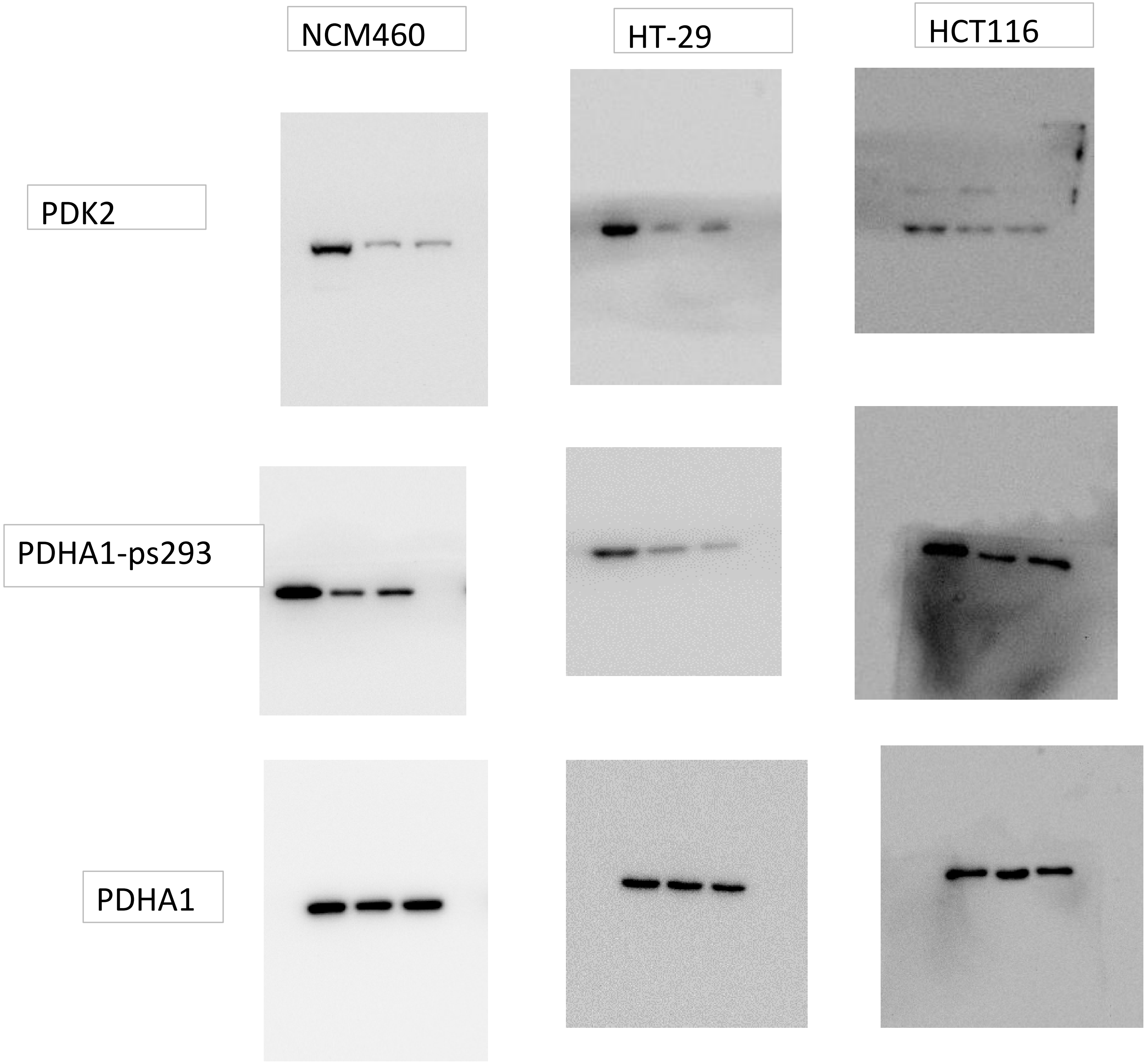

FigureS3M

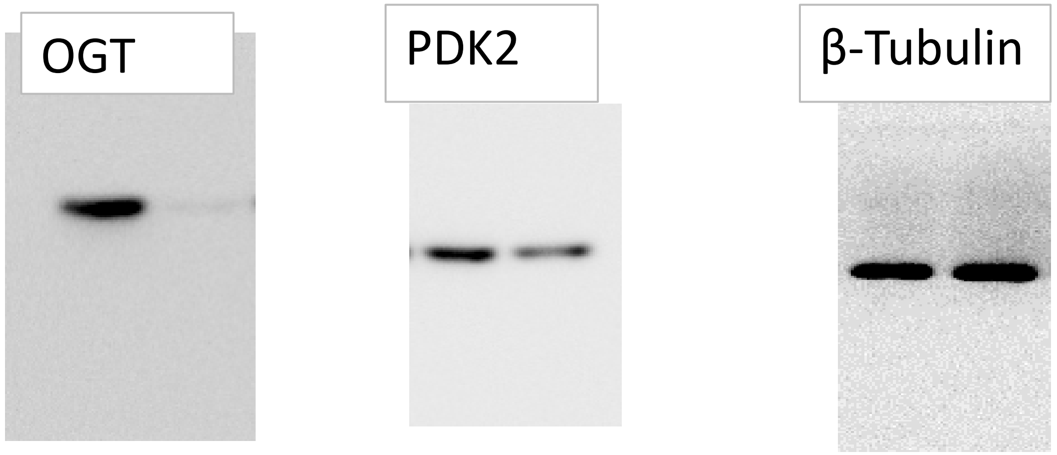

FigureS3O

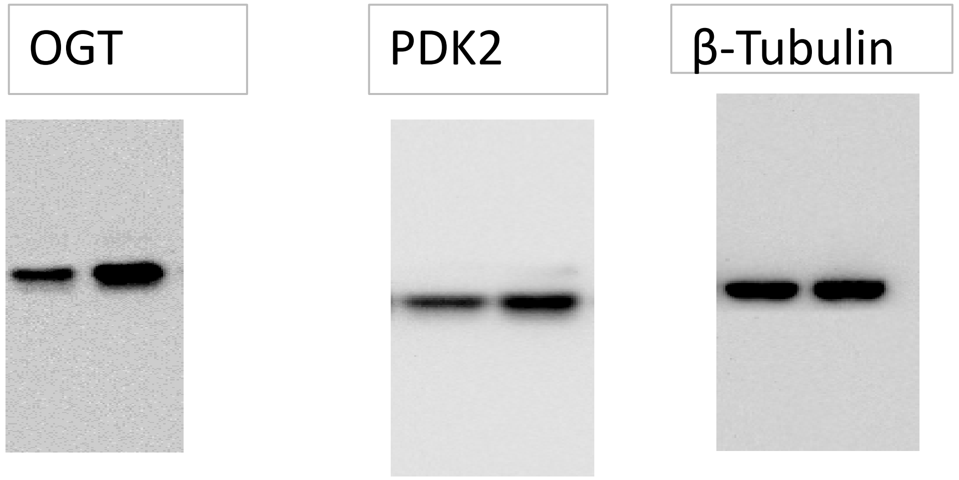

FigureS4B

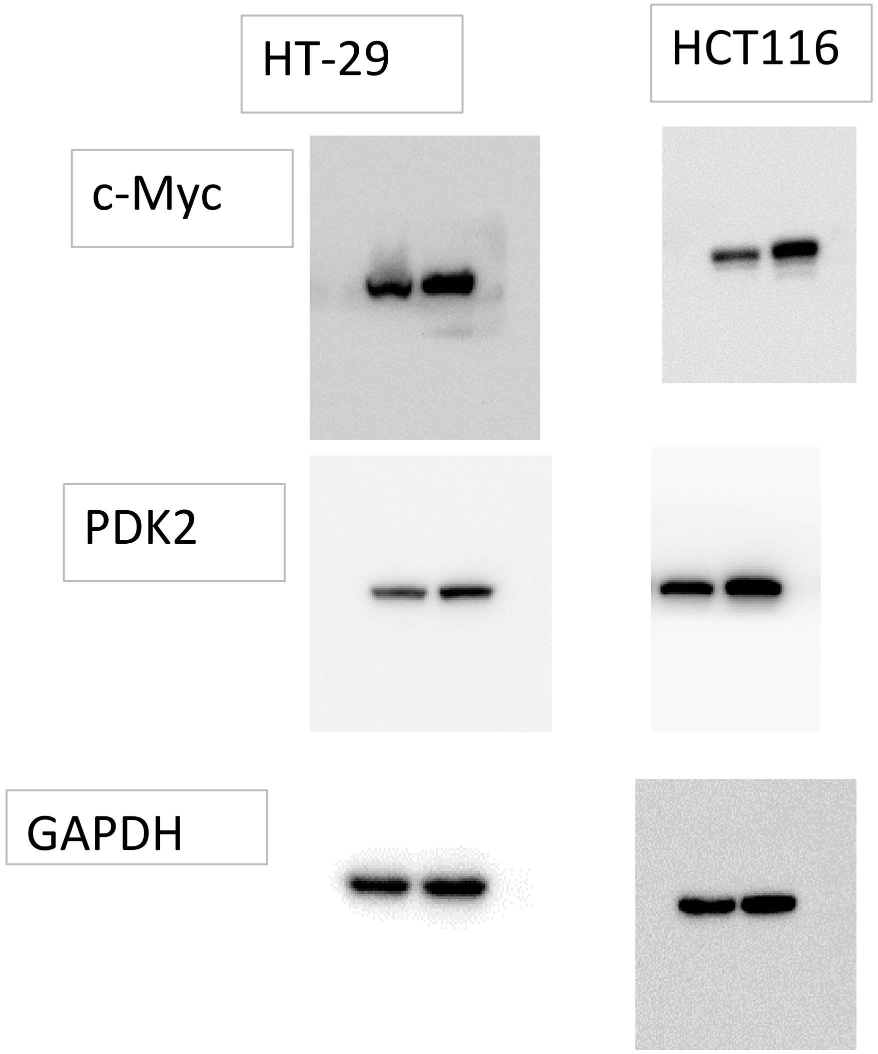

FigureS4E

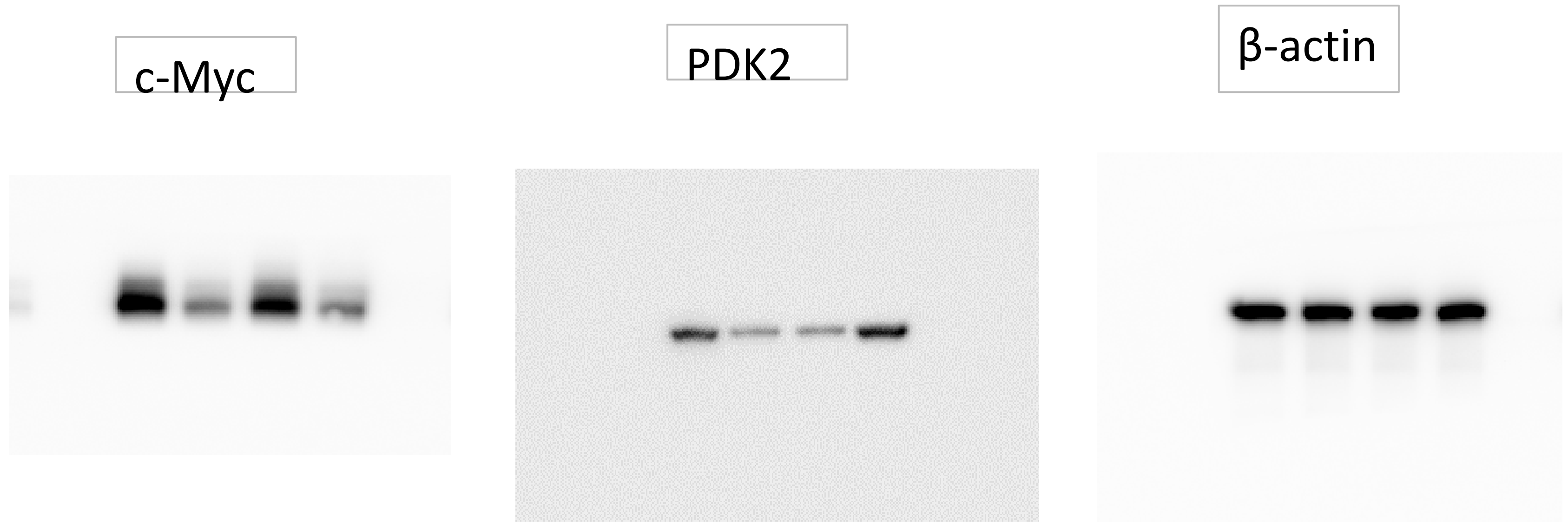

FigureS5F

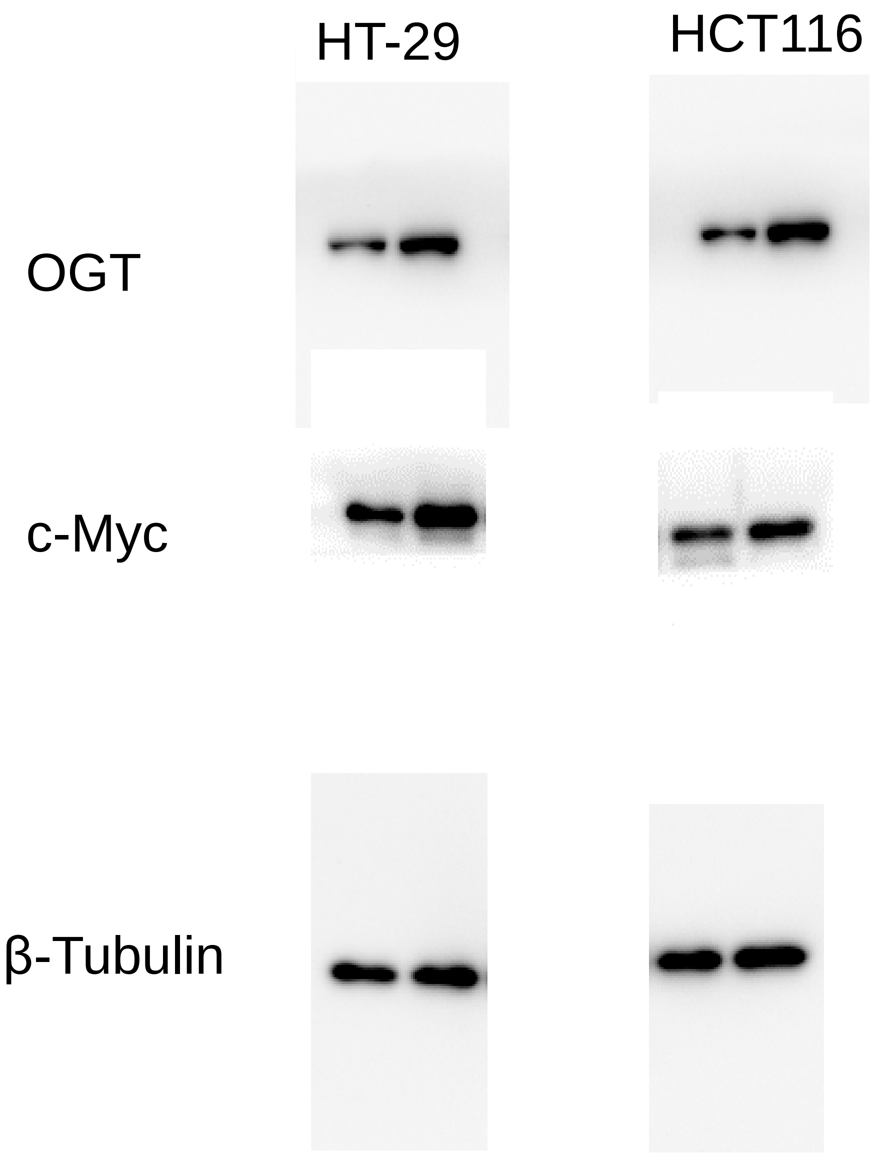

FigureS5I

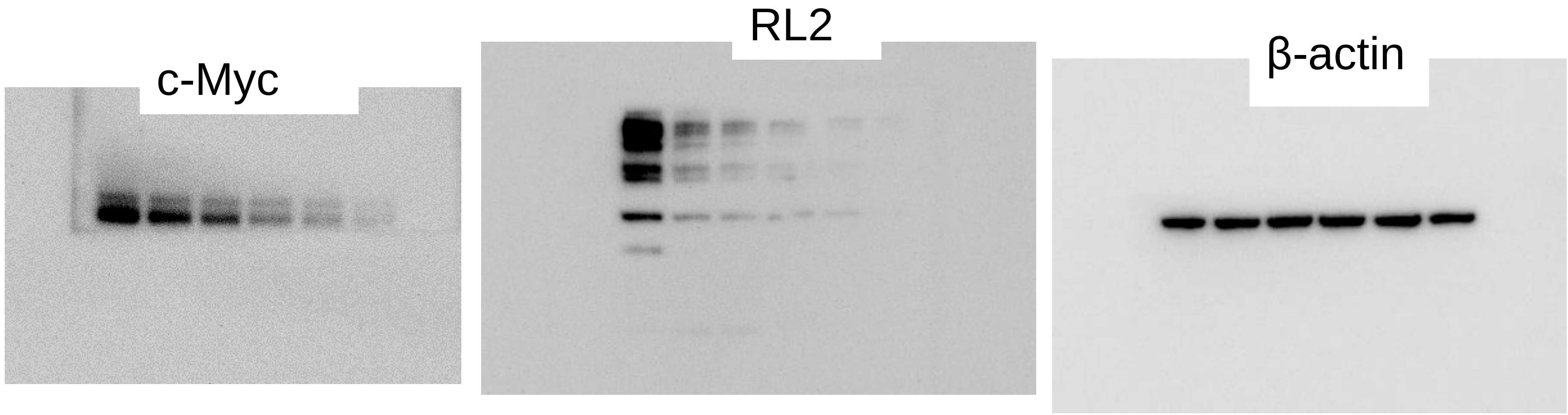

FigureS6A

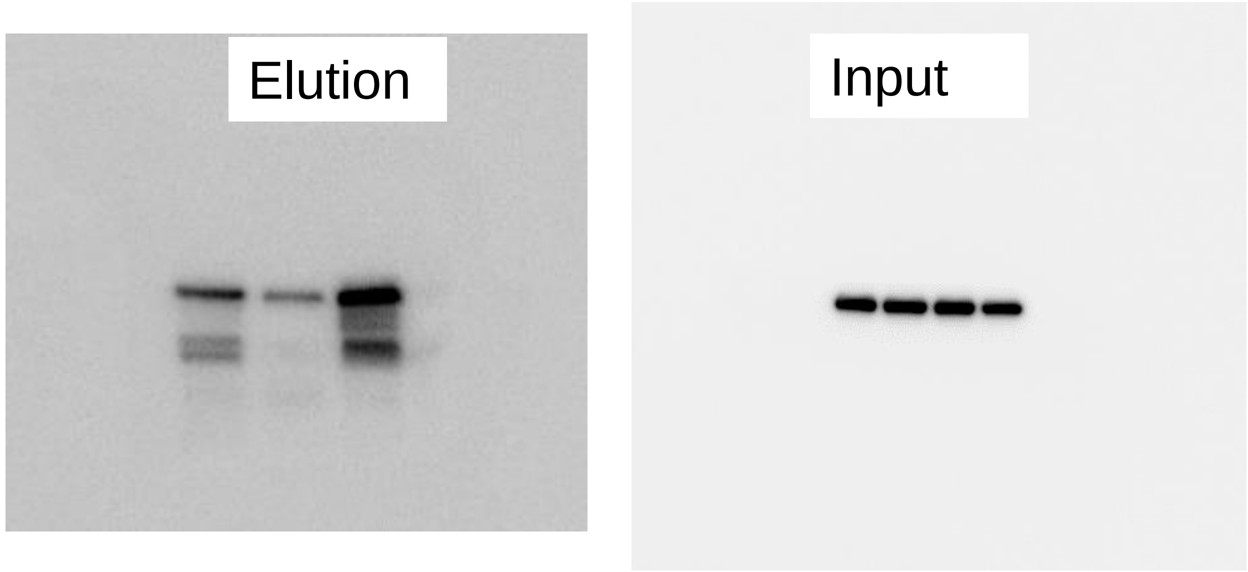

FigureS6B

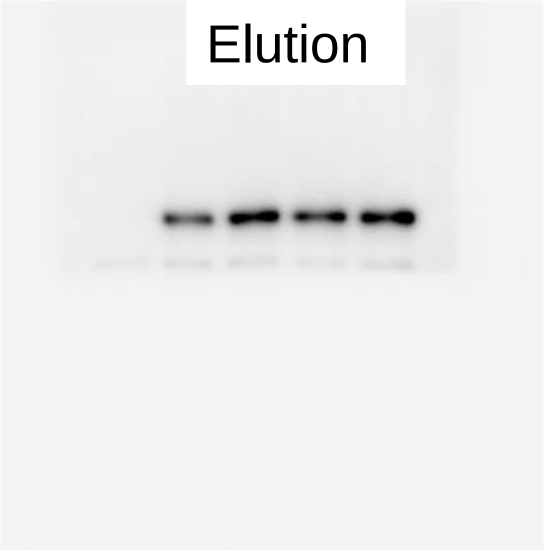

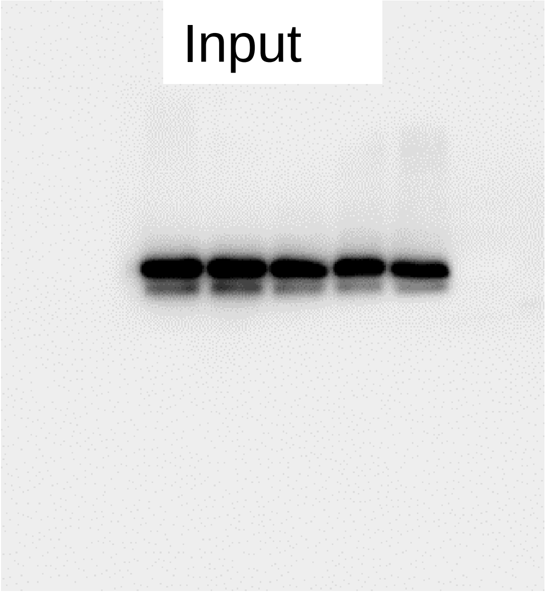

FigureS6C

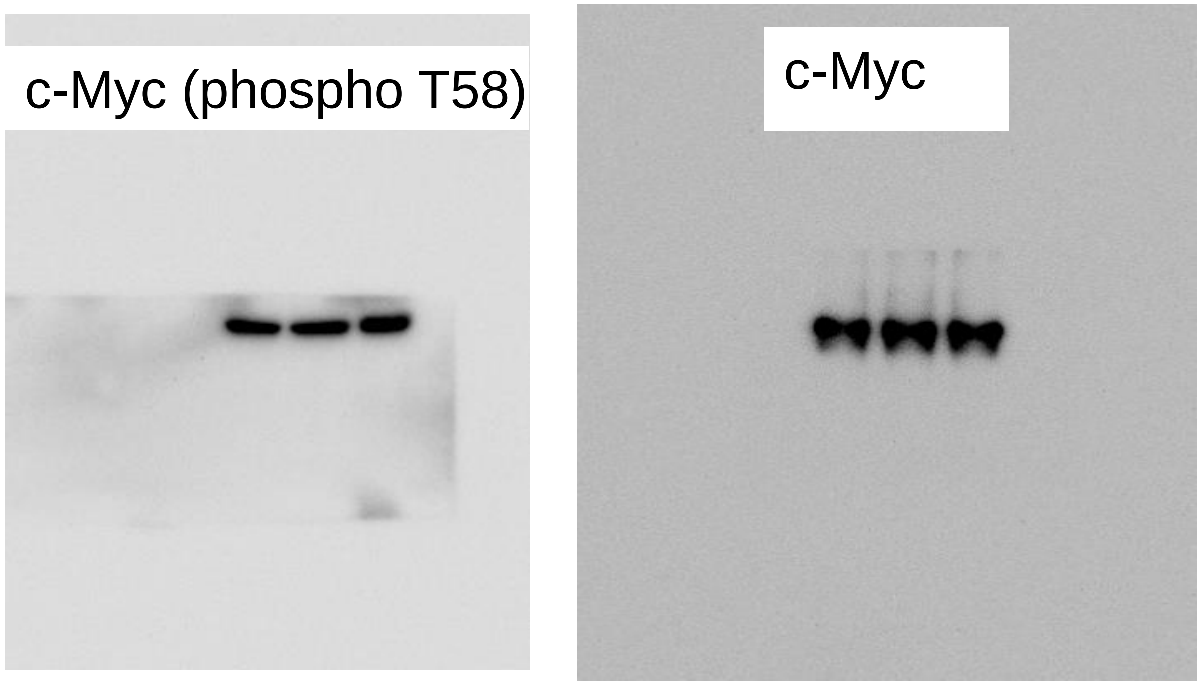

FigureS6D

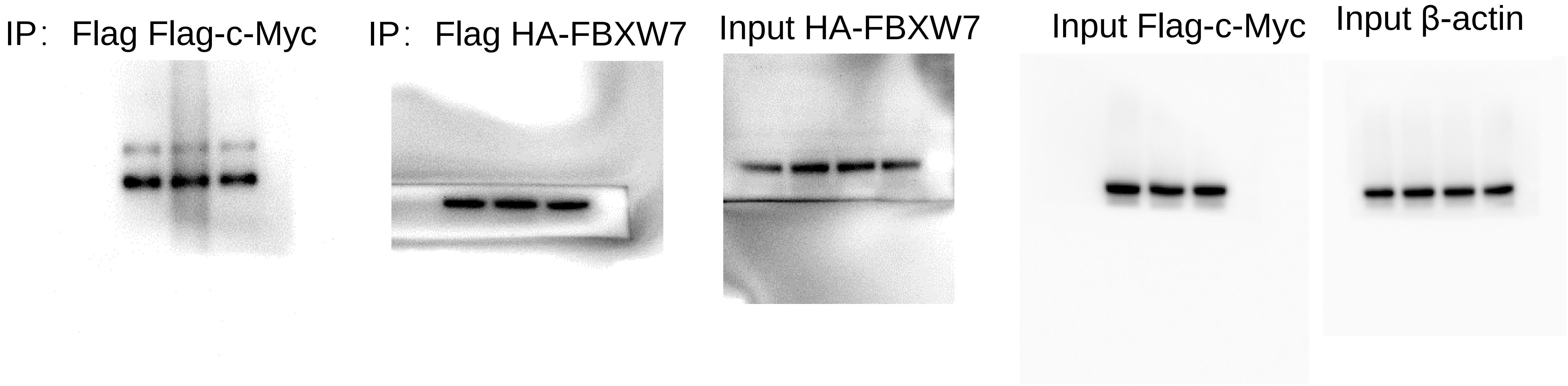
IP： Flag Flag-c-Myc
IP： Flag HA-FBXW7
Input HA-FBXW7
Input Flag-c-Myc
Input β-actin

FigureS6E

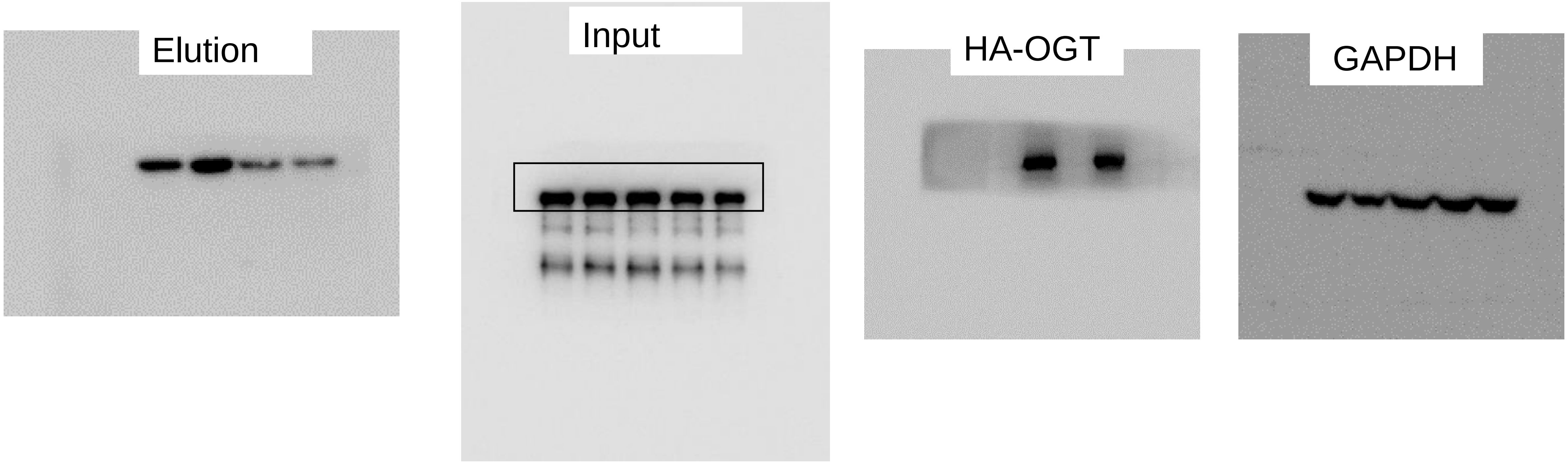

FigureS6G

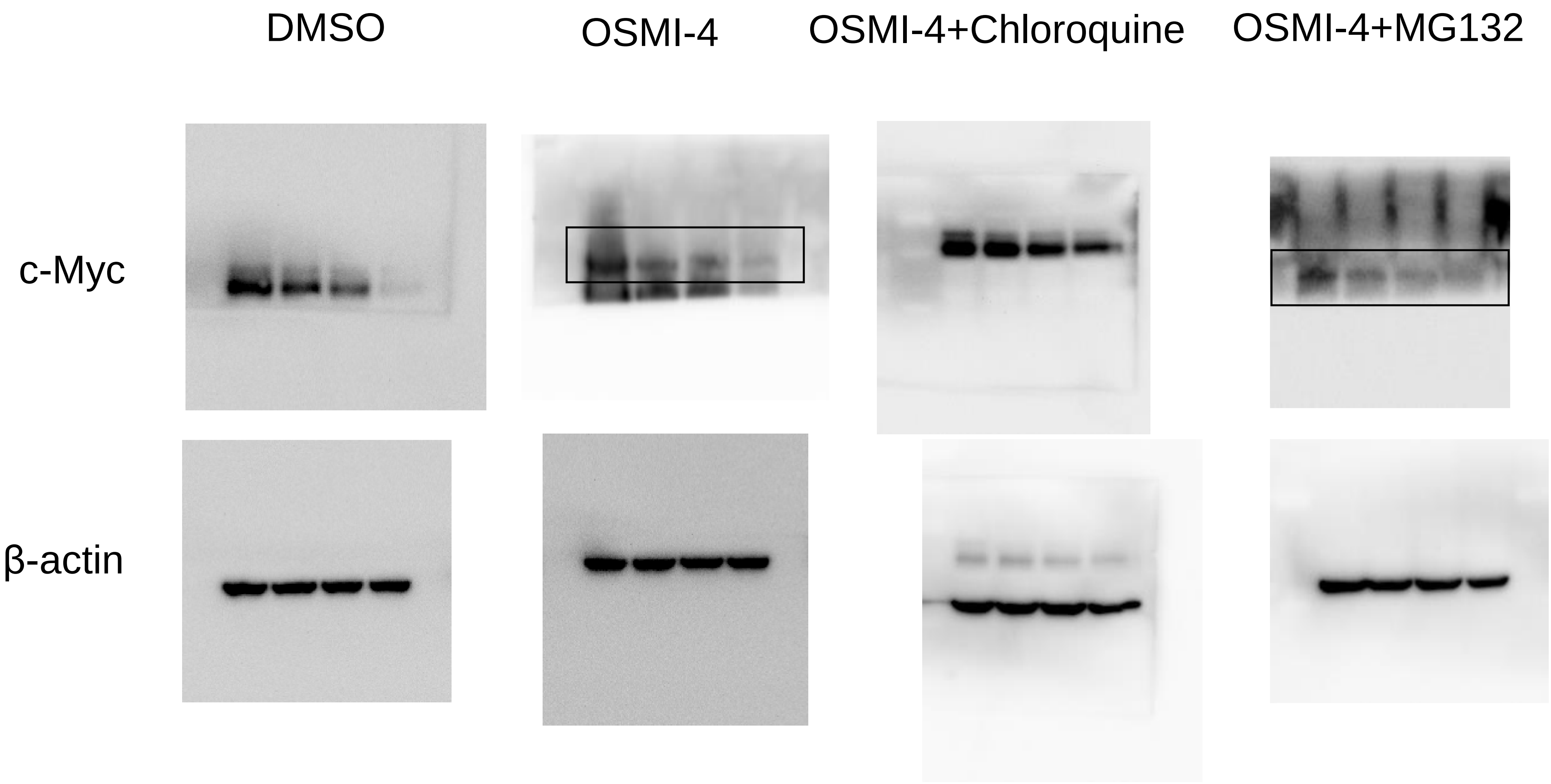

FigureS6H

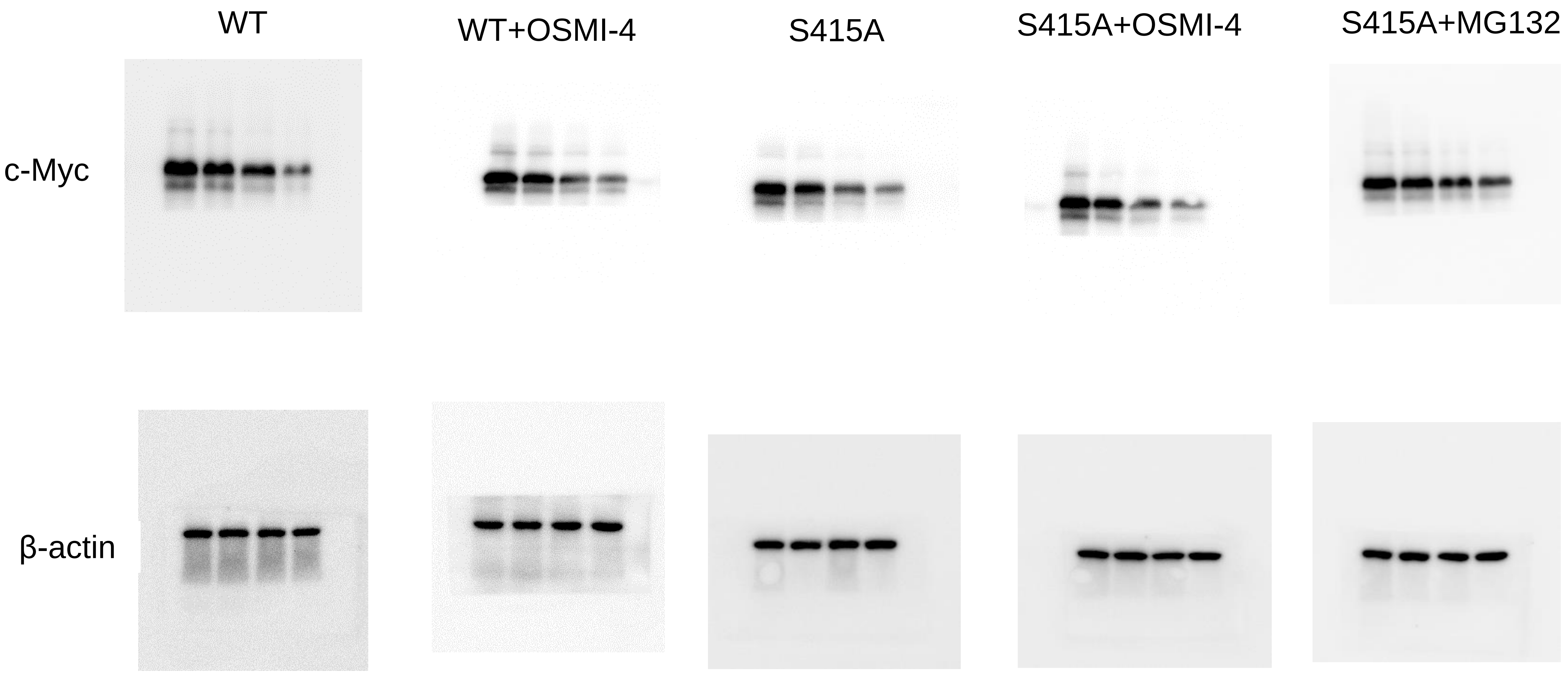

FigureS7A

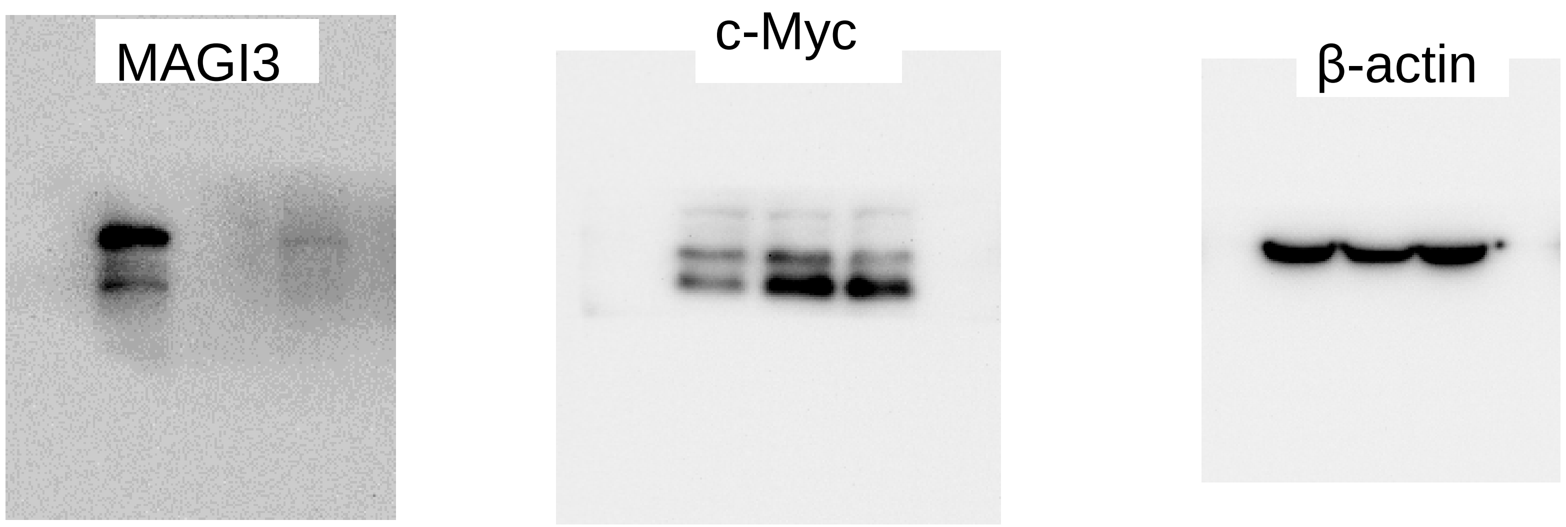

FigureS7B

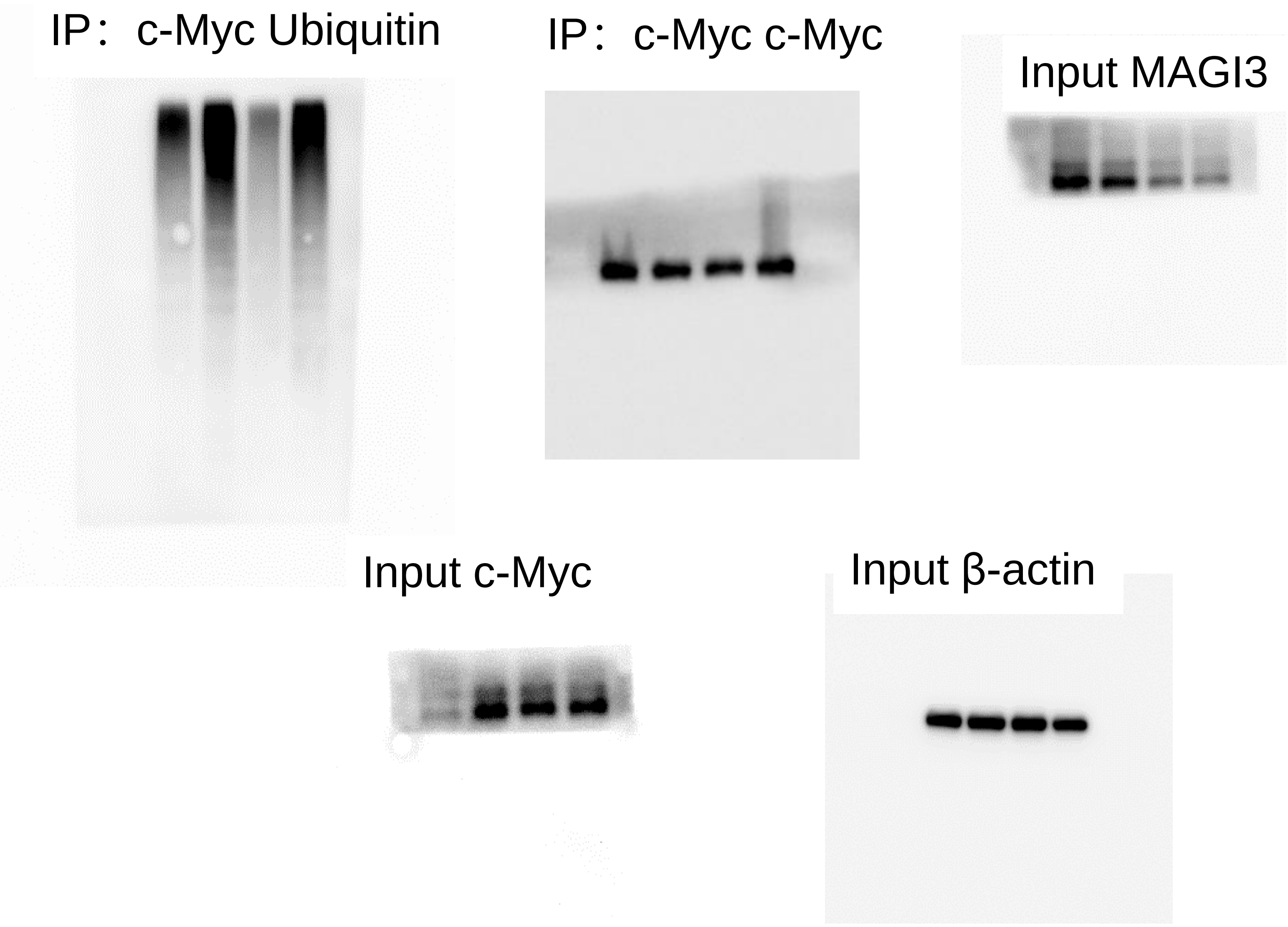
IP：c-Myc Ubiquitin
IP：c-Myc c-Myc
Input MAGI3
Input c-Myc
Input β-actin

FigureS7C

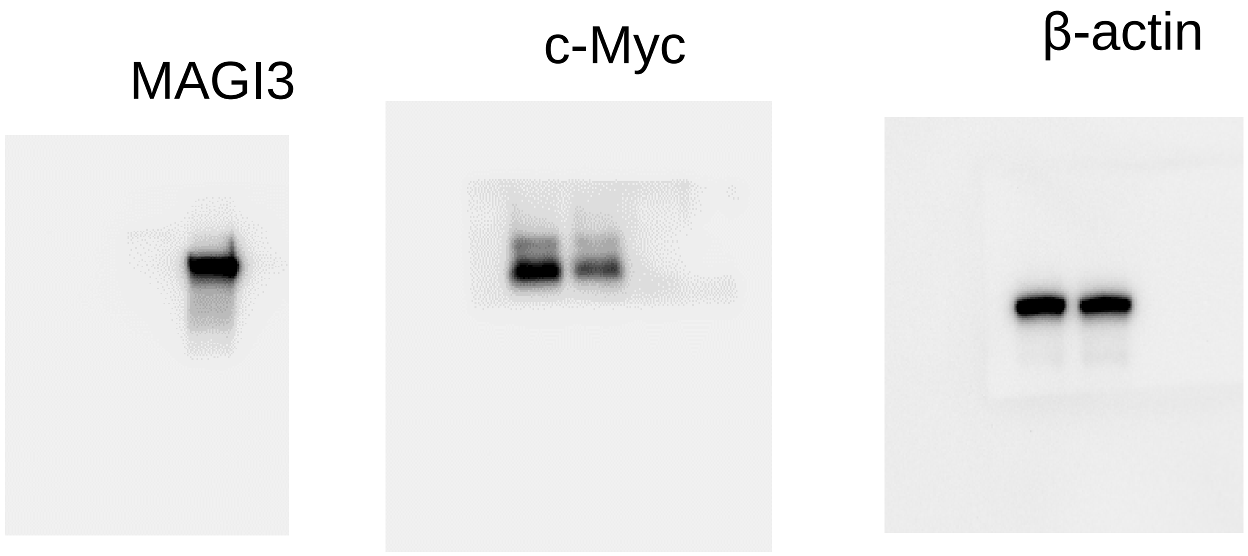

FigureS7D

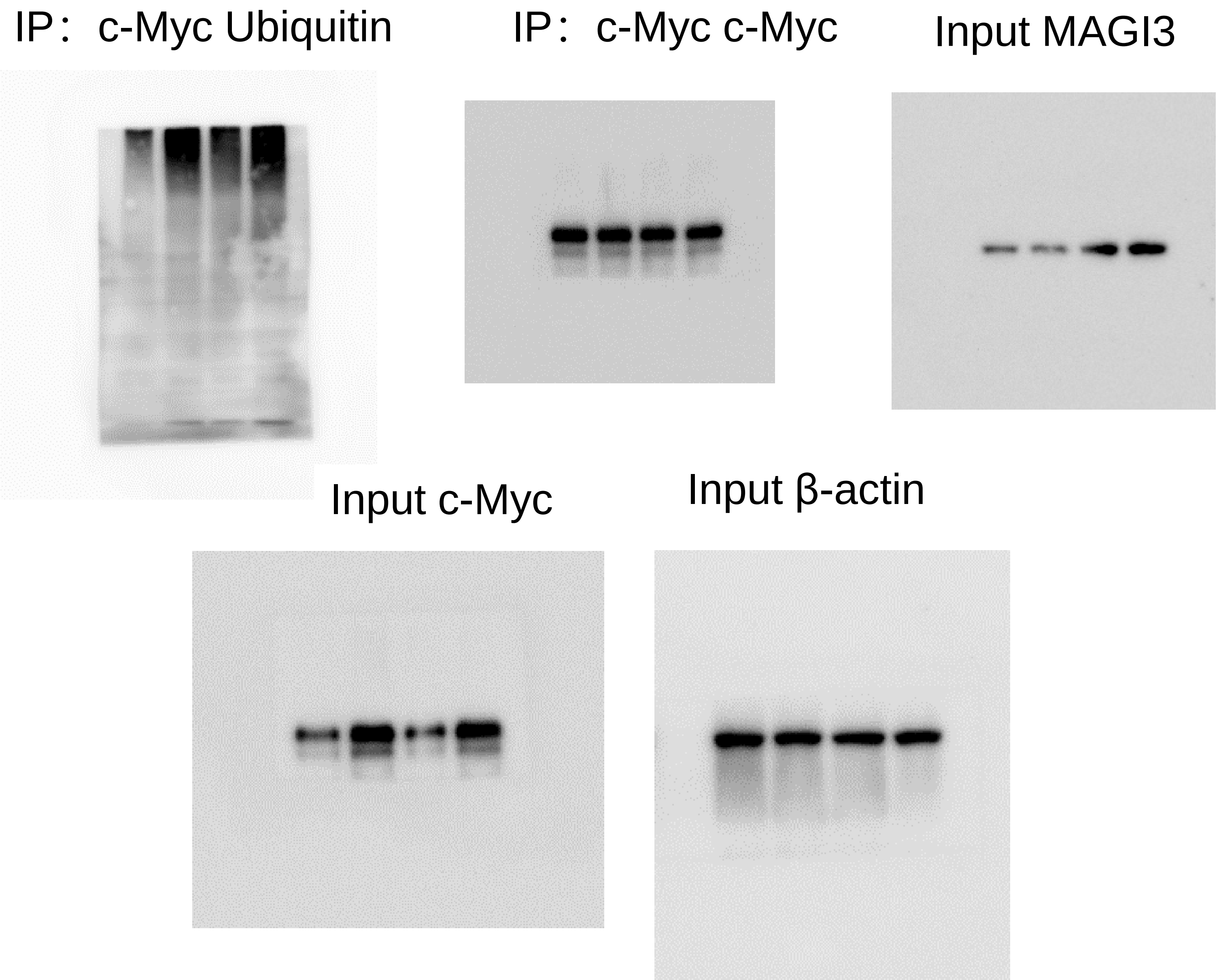
IP： c-Myc Ubiquitin
IP： c-Myc c-Myc
Input MAGI3
Input c-Myc
Input β-actin

FigureS7E

IP:HA Flag-c-Myc

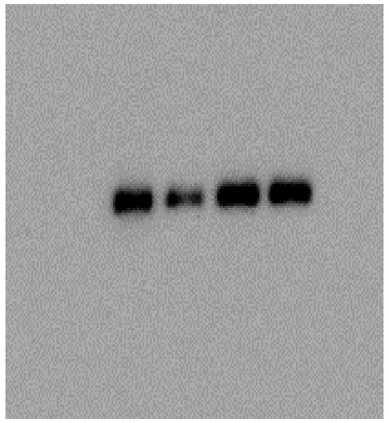

IP:HA HA-MAGI3

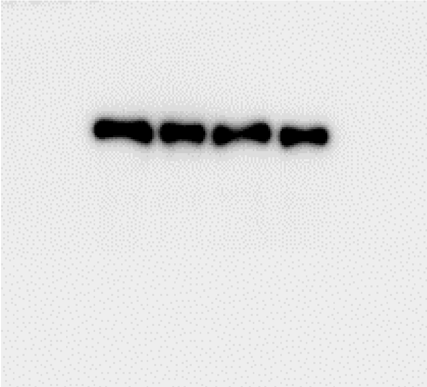

Input Myc-OGT

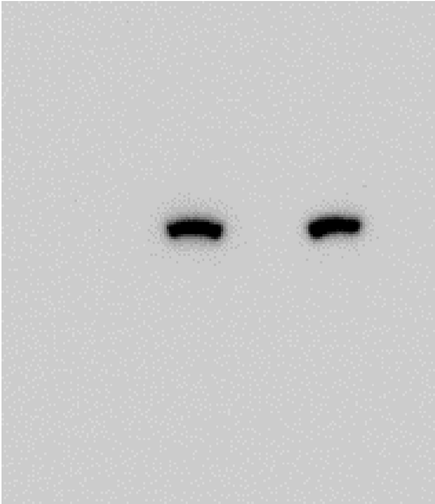

Input HA-MAGI3

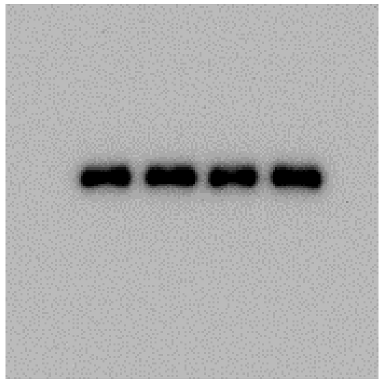

Input Flag-c-Myc

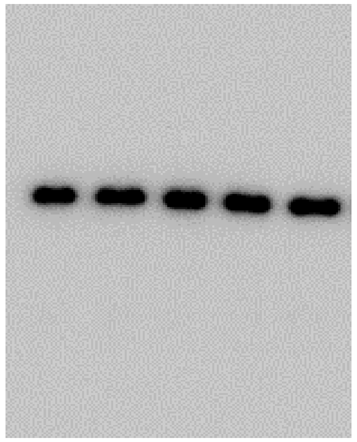

FigureS8A

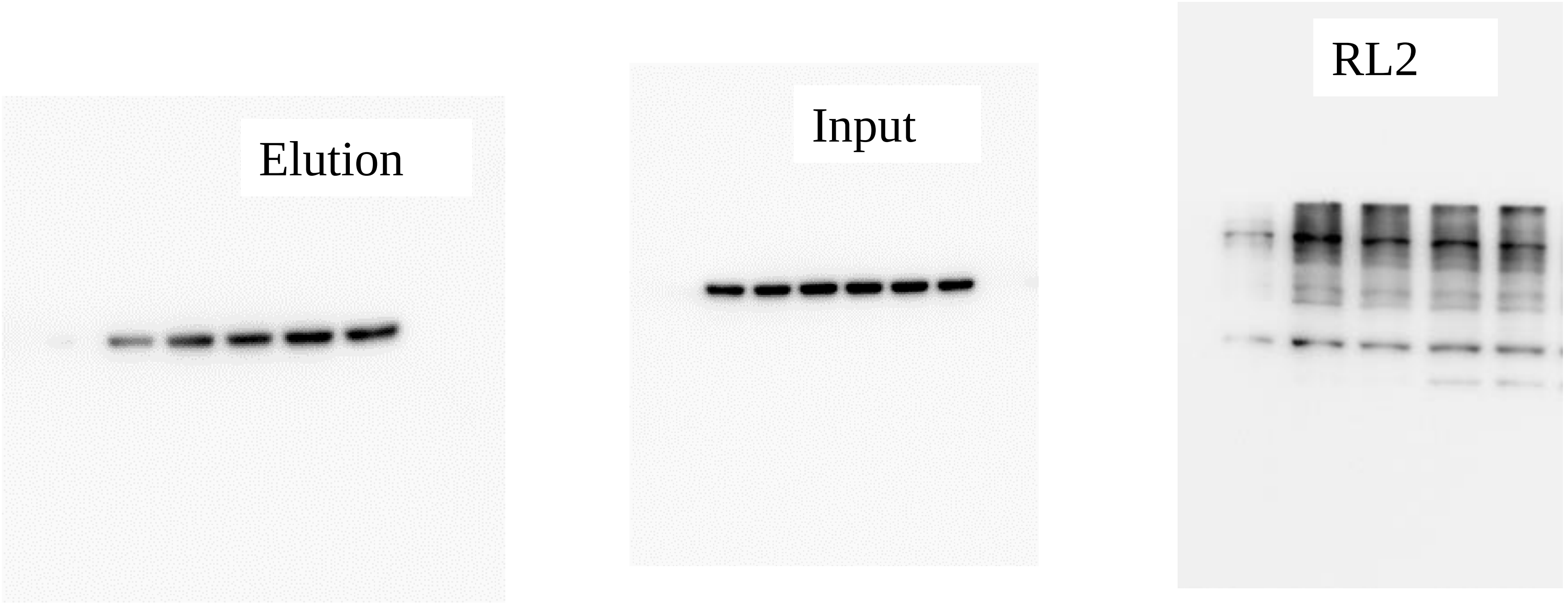

FigureS9D

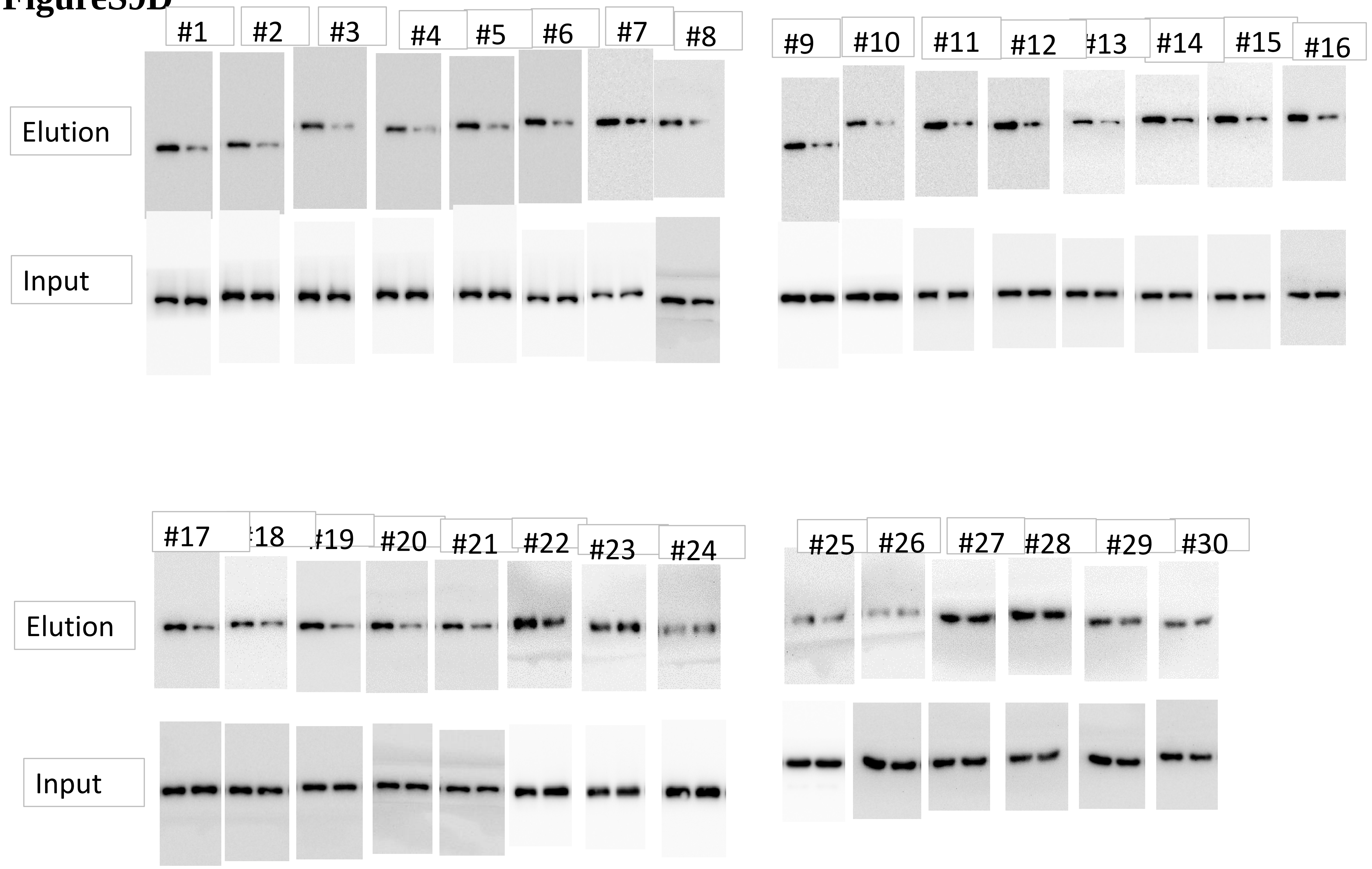
#1
#2
#3
#4
#5
#6
#7
#8
#9
#10
#11
#12
13
#14
#15
#16
Elution
Input
#17
18
19
#20
#21
#22
#23
#24
#25
#26
#27
#28
#29
#30
Elution
Input
